# Study Design, Protocol and Profile of the Maternal And Developmental Risks from Environmental and Social Stressors (MADRES) Pregnancy Cohort: a Prospective Cohort Study in Predominantly Low-Income Hispanic Women in Urban Los Angeles

**DOI:** 10.1186/s12884-019-2330-7

**Published:** 2019-05-30

**Authors:** Theresa M. Bastain, Thomas Chavez, Rima Habre, Mariam S. Girguis, Brendan Grubbs, Claudia Toledo-Corral, Milena Amadeus, Shohreh F. Farzan, Laila Al-Marayati, Deborah Lerner, David Noya, Alyssa Quimby, Sara Twogood, Melissa Wilson, Leda Chatzi, Michael Cousineau, Kiros Berhane, Sandrah P. Eckel, Fred Lurmann, Jill Johnston, Genevieve F. Dunton, Frank Gilliland, Carrie Breton

**Affiliations:** 1 Department of Preventive Medicine, USC Keck School of Medicine, University of Southern California, 2001 N. Soto Street, M/C 9237, Los Angeles, CA 90032 USA; 2 Department of Obstetrics and Gynecology, University of Southern California, Los Angeles, CA USA; 3 Department of Public Health, California State University Northridge, Los Angeles, CA USA; 4 Eisner Health, Los Angeles, CA USA; 5 South Central Family Health Center, Los Angeles, CA USA; 6 Sonoma Technology Inc, Petaluma, CA USA; 7 Department of Psychology, University of Southern California, Los Angeles, CA USA

**Keywords:** MADRES, Maternal health, Obesity, Childhood obesity, Health disparities, Stress, Environment, Birth outcomes

## Abstract

**Background:**

The burden of childhood and adult obesity disproportionally affects Hispanic and African-American populations in the US, and these groups as well as populations with lower income and education levels are disproportionately affected by environmental pollution. Pregnancy is a critical developmental period where maternal exposures may have significant impacts on infant and childhood growth as well as the future health of the mother. We initiated the “Maternal And Developmental Risks from Environmental and Social Stressors (MADRES)” cohort study to address critical gaps in understanding the increased risk for childhood obesity and maternal obesity outcomes among minority and low-income women in urban Los Angeles.

**Methods:**

The MADRES cohort is specifically examining whether pre- and postpartum environmental exposures, in addition to exposures to psychosocial and built environment stressors, lead to excessive gestational weight gain and postpartum weight retention in women and to perturbed infant growth trajectories and increased childhood obesity risk through altered psychological, behavioral and/or metabolic responses. The ongoing MADRES study is a prospective pregnancy cohort of 1000 predominantly lower-income, Hispanic women in Los Angeles, CA. Enrollment in the MADRES cohort is initiated prior to 30 weeks gestation from partner community health clinics in Los Angeles. Cohort participants are followed through their pregnancies, at birth, and during the infant’s first year of life through a series of in-person visits with interviewer-administered questionnaires, anthropometric measurements and biospecimen collection as well as telephone interviews conducted with the mother.

**Discussion:**

In this paper, we outline the study rationale and data collection protocol for the MADRES cohort, and we present a profile of demographic, health and exposure characteristics for 291 participants who have delivered their infants, out of 523 participants enrolled in the study from November 2015 to October 2018 from four community health clinics in Los Angeles. Results from the MADRES cohort could provide a powerful rationale for regulation of targeted chemical environmental components, better transportation and urban design policies, and clinical recommendations for stress-coping strategies and behavior to reduce lifelong obesity risk.

**Electronic supplementary material:**

The online version of this article (10.1186/s12884-019-2330-7) contains supplementary material, which is available to authorized users.

## Background

The prevalence of obesity has continued to increase in the United States, but not all populations are affected equally [1]. The burden of childhood obesity disproportionally affects Hispanic and African-American populations [2, 3]. Recent data from NHANES continue to show that these groups face the highest burden of childhood obesity [4]. Moreover, pregnancy-related obesity rates are disproportionately high in minority women [5, 6]. Eliminating racial/ethnic disparities in obesity is a critical priority, especially given the numerous adverse cardio-metabolic sequelae [7].

Not only do some racial and ethnic groups suffer from health disparities, but these groups, as well as populations with lower income and education levels, are disproportionately affected by environmental pollution [8, 9]. In California, the burden of exposures to multiple environmental chemicals is not evenly distributed, with Hispanic and African-American populations carrying the greatest cumulative burden of harmful environmental exposures [10].

Evidence is increasing that certain environmental chemicals have “obesogenic” properties and alter the metabolic profile of adipose tissue [11, 12]. Air pollution is one ubiquitous exposure that is increasingly associated with obesity risk [13]; however, there remains inadequate research linking prenatal air pollution exposures to obesity risk, especially among health disparate populations. Pregnancy is a critical developmental period where maternal exposures may have significant impacts on infant and childhood growth as well as the future health of the mother.

We initiated the “Maternal And Developmental Risks from Environmental and Social Stressors (MADRES)” cohort study to address critical gaps in understanding the increased risk for childhood obesity and maternal obesity outcomes among minority and low-income women in urban Los Angeles and to provide evidence and identify key targets for policy, clinical and programmatic intervention to reduce the burden born by these individuals. The MADRES cohort is specifically examining whether pre- and postpartum environmental exposures, coupled with exposures to psychosocial and built environment stressors, lead to excessive gestational weight gain and postpartum weight retention in women and to perturbed infant growth trajectories and increased childhood obesity risk through altered psychological, behavioral and/or metabolic responses.

In this paper, we describe the design, data collection protocol and cohort profile from participants who enrolled and delivered between November 2015 and October 2018 in the ongoing MADRES cohort.

## Methods/design

### Design overview

The MADRES cohort is an ongoing prospective pregnancy cohort of 1000 predominantly lower-income, Hispanic women in Los Angeles, California. Enrollment in the MADRES cohort is initiated prior to 30 weeks gestation through partnerships with four prenatal care providers in Los Angeles. Cohort participants are followed through their pregnancies, at birth, and through the infant’s first year of life through a series of in-person visits with interviewer-administered questionnaires, anthropometric measurements and biospecimen collection as well as through telephone interviews conducted with the mother (see Table 1).

**Table 1:** Data Collection for Expected 1000 Mother-Child Pairs in the Maternal And Developmental Risks from Environmental and Social Stressors (MADRES) Cohort

	Pregnancy Trimester Timepoints			Birth	Postnatal Timepoints					Data Source
	1st	2nd	3rd		7–14 days	1 month	3 months	6 months	12 months	
	< 20 wks	18–27 wks	30–34 wks							
Maternal variables										
Demographics	X	X	X							Questionnaire
Medical history			X							Questionnaire
Pregnancy and delivery outcomes	X	X	X	X	X					Questionnaire/MR
Lifestyle, including smoking	X	X	X			X	X	X	X	Questionnaire
24-h dietary recall(s): ASA24			X						X	Questionnaire
Physical activity: PPAQ	X		X				X		X	Questionnaire
Sleep duration and quality	X	X	X				X	X	X	Questionnaire
Stress and depression	X	X	X				X	X	X	Questionnaire
Household environmental exposures	X	X	X			X	X	X	X	Questionnaire
Residential and occupational history			X							Questionnaire
Pre-pregnancy weight	X									Questionnaire
Weight	X	X	X	X		X			X	Clinic Visit/MR
Height	X	X	X	X		X			X	Clinic Visit/MR
Waist circumference						X			X	Clinic Visit/MR
Gestational weight gain	X	X	X	X						Clinic Visit/MR
Postpartum weight retention						X			X	Clinic Visit/MR
Infant variables										
Birth weight				X	X					Questionnaire/MR
Birth length				X	X					Questionnaire/MR
Gestational age at birth				X						MR
Infant sex				X	X					Questionnaire/MR
Infant weight				X	X	X	X	X	X	Clinic Visit/MR
Infant length				X	X	X	X	X	X	Clinic Visit/MR
Head circumference				X	X	X	X	X	X	Clinic Visit/MR
Infant feeding and breastfeeding					X	X	X	X	X	Questionnaire
Developmental milestones						X	X	X	X	Questionnaire
Infant health and immunizations				X	X	X	X	X	X	Questionnaire/MR

MR: Medical Record; ASA24: Automated Self-Administered 24-h Recall; PPAQ: Pregnancy Physical Activity Questionnaire

Informed consent and HIPAA authorization to access medical records is obtained at study entry for each participant and her child. The University of Southern California’s Institutional Review Board approved the protocol. Study data are collected and managed using REDCap electronic data capture tools hosted at the University of Southern California.

### Eligibility, enrollment, and follow-up

Beginning in November 2015, participants have been recruited from prenatal care providers in Los Angeles serving predominantly medically-underserved populations. Participants are recruited from two non-profit community health clinics, one county hospital prenatal clinic, one private obstetrics and gynecology practice, and through self-referral from community meetings and local advertisements. Eligible women are (1) at less than 30 weeks gestation at the time of recruitment, (2) over 18 years of age, and (3) speak either English or Spanish fluently. Exclusion criteria include (1) HIV positive status, (2) physical, mental, or cognitive disability that prevents participation or providing informed consent, (3) current incarceration, or (4) multiple gestation.

At each partner clinic site, MADRES investigators have established relationships with a lead clinic physician or other healthcare provider to enable access to and assistance with identifying potential eligible participants who are able to enroll at less than 30 weeks gestation and meet basic inclusion criteria. MADRES staff maintain close relationships with the clinic nursing staff and providers and ensure they are familiar with the study protocols, exclusion criteria, and consent forms. MADRES staff approach potentially eligible women at each clinic site, explain the study procedures and goals to potential participants, and obtain informed consent for study procedures and HIPAA authorization to abstract medical records for mother and child through 12 months of age. Study visits for data collection occur at USC or by telephone after informed consent is obtained at the recruitment sites.

Advertisements placed in local papers or in community locations indicate selected criteria for self-referral into the study including (1) pregnant carrying only one baby less than 30 weeks gestation; (2) over 18 years of age, (3) living within 20 miles of downtown Los Angeles (to approximate the same distance from the community health clinic recruitment sites); and (4) eligible for WIC or Medi-Cal (to mirror the populations receiving prenatal care at the community health clinic sites). Women who contact the study (via phone or email) complete a brief questionnaire by phone to confirm study eligibility. Those meeting study eligibility are invited to an in-person visit with MADRES study staff to complete the informed consent and procedures for the study.

For women enrolling at less than 20 weeks gestation, the first study visit is scheduled within 2 weeks of recruitment to (1) complete an interviewer-administered questionnaire in English or Spanish, (2) measure height and weight, and (3) collect biospecimens (see Table 2 for a full list). Follow-up questionnaires are conducted by phone between 18 and 27 weeks, and then an in-person study visit is scheduled between 30 and 34 weeks where a study interview is administered, height and weight are measured, and biospecimens are again collected. For women enrolling between 20 and 30 weeks gestation, the first study visit occurs in person between 28 and 36 weeks to complete an interviewer-administered questionnaire, measure height and weight, and collect biospecimens.

**Table 2:** Biospecimen Collection Protocol for Mother-Child Pairs in the Maternal and Developmental Risks from Environmental and Social Stressors (MADRES) Pregnancy Cohort

	Pregnancy Trimester		Birth	Postnatal Timepoints	
	1st	3rd		1 month	12 months
Maternal Blood	X	X			X
Maternal Hair	X	X			
Maternal Nails	X	X			X
Maternal Urine	X	X			X
Maternal Stool	X				
Maternal Saliva		X			
Umbilical Cord Blood			X		
Placenta			X		
Newborn Blood Spot			X		
Infant Hair					X
Infant Nails					X
Infant Stool				X	X

At birth, selected biospecimens are collected at participants’ delivering hospitals according to procedures developed in conjunction with each delivering hospital.

Following birth, study staff maintain regularly scheduled contact with enrolled women by phone, email or text message. Women complete a brief phone interview between 7 and 14 days after birth; and more detailed phone interviews at 3 month intervals after birth. At 1 month and 12 months after birth, in-person study visits at USC are scheduled to conduct interviewer-administered questionnaires, measure height and weight for both mother and baby, and collect biospecimens from both mother and baby.

### Retention

We employ proactive and thorough participant-tracking techniques and document changes in residential status. We ask women at baseline (e.g. initial visit) to provide contact information including home address, telephone numbers (day and evening), e-mail address and contact information for next of kin and a close friend. Regular contact is maintained with participants recruited into the study population. To help with subject tracking and retention, participants are provided with small gifts timed to coincide with baby milestones (e.g., birth, birthdays, etc.) which serve both as an expression of gratitude for continued participation and to maintain subject contact. Quarterly newsletters are physically and electronically mailed to study participants and semi-annual participant appreciation events are held to maintain contact with study participants. When MADRES staff are unable to reach study participants, they review of change-of-address reports and attempt to contact participants through next-of-kin or participant-identified friends.

### Specific data collection procedures

An overview of the major data collection protocols and endpoints for MADRES participants from study enrollment in pregnancy through twelve months after birth is shown in Table 1. Broadly, MADRES data collection procedures combine questionnaire measures administered orally by trained bilingual staff, (see Additional files 1, 2, 3, 4, 5, 6, 7, 8, 9, 10, 11, 12, 13, 14, 15, 16, 17, 18, 19, 20, 21, 22, 23, 24, 25, 26, 27, and 28), electronic medical records data acquisition, and in- person measurements and biospecimen collection for both mother and child participants.

#### Primary outcome measures

##### Maternal gestational weight gain at delivery and postpartum weight retention at 12 months

Maternal height and weight are measured at the initial prenatal visit (if women are enrolled at prior to 20 weeks gestation), at the third trimester visit, and at the 1-month and 12-month postpartum visits. Maternal weight is measured on a digital scale (Tanita) accurate to the nearest 5 g. Two measurements are taken for each participant and a third measurement is taken if the first two measurements differ by more than 0.1 pounds (0.05 kg), following PhenX protocol. Standing height is measured using a Perspectives Enterprises Model PE-AIM-101 stadiometer, following PhenX protocol. Two measurements are taken for each participant and a third is taken if the first two measurements differ by more than 1 cm. For both height and weight, the average of the two measurements in closest agreement is calculated.

Maternal gestational weight gain is defined as the difference between a mother’s weight measured at specific points of time during pregnancy and her self-reported pre-pregnancy weight. Postpartum weight retention is defined as the difference in a mother’s weight measured postpartum and her pre-pregnancy weight. We will generate values for postpartum weight retention at 1- and 12-months post-delivery adjusted for number of weeks post-delivery.

##### Infant birth weight

Birth weight is abstracted from the electronic or paper medical record of the delivery. Birth weight less than 2500 g in infants is defined as low birth weight (LBW), while children in less than the 10th percentile of predicted birth weight based on gestational age and sex in term infants are considered as small for gestational age (SGA), as previously described [14].

##### Infant growth from birth to 12 months of age

Infant length and weight are measured at in-person visits at 1 month and 12 months. Infant weight is measured, while the mother holds the infant, on a digital scale (Seca 874) accurate to the nearest 5 g. Two measurements are taken by two alternating study staff. If the two measurements differ by more than 0.05 kg, a third measurement is taken by the first measuring study staff. Length is measured to the nearest 0.1 cm using a Seca 416 infantometer. Two measurements are taken by two alternating study staff while the mother is holding the infant’s head steady. If the two measurements differ by more than 0.1 cm, a third measurement is taken by the first measuring study staff. For both weight and length, the average of the two measurements in closest agreement is calculated. Infant lengths and weights are also abstracted from the pediatric medical records from all well-baby visits and growth curves are estimated for each child.

#### Participant-reported outcomes and exposures

##### Maternal stress and psychosocial functioning measures during pregnancy

During pregnancy study timepoints, maternal participants are verbally-administered several validated stress or psychosocial functioning instruments. In each pregnancy trimester, participants are administered Cohen’s 10-item Perceived Stress Scale (PSS) [15], the trimester-progressive Prenatal Distress Questionnaire [16], and the Center for Epidemiological Studies- Depression (CES-D) Scale [17]. In the second trimester, participants are administered the Financial Stress Scale [18], the Adverse Childhood Experiences (ACEs) questionnaire [19], and questions about perceived neighborhood violence, personal victimization and neighborhood social cohesion from Sampson et al. [20] In the third trimester, participants are administered the Modified Life Events Inventory regarding stressful life events over pregnancy in the last 6 months [21].

##### Maternal postpartum stress and psychosocial functioning

After birth, maternal participants are administered the PSS [15] in each postpartum questionnaire at 3, 6 and 12 months and the CES-D [17] at 12 months. Women are administered 9 items from the 10-item Postpartum Distress Measure [22] at 3, 6 and 12 months. At 6 months, women answer questions about neighborhood social cohesion [20] and are administered the Financial Stress Scale [18].

##### Maternal physical activity assessment

Maternal physical activity during pregnancy is assessed at the initial study visit (if enrolled prior to 20 weeks) and at the third trimester study visit using the Pregnancy Physical Activity Questionnaire (PPAQ), a validated measure of 32 physical activities including household/caregiving, occupational, sports/exercise, transportation, and inactivity in pregnancy [23]. The PPAQ has shown high reproducibility and has been validated, with modest correlation, against gold standard measures of physical activity, in an ethnically-diverse prenatal population [23]. Physical activity is assessed at the 3-month and 12-month postpartum timepoints using a modified PPAQ, replacing questions that refer to a trimester window with an estimate of activity within the previous 30 days.

##### Maternal dietary intake assessment

Maternal dietary intake is assessed two times (one weekday and one weekend day) in the 3rd trimester and two times (one weekday and one weekend day) at 12 months postpartum by adapting the validated Automated Self-Administered 24 h Dietary Assessment (ASA24®), a web-based instrument that enables multiple, automatically-coded, 24-h dietary recalls [24], for interviewer-assisted administration. Participants are asked by MADRES interviewers to recall all food and beverages consumed on the previous day, from midnight to midnight and data are entered into the ASA24 database by MADRES interviewers. Collected dietary data include time of consumption; where meals were eaten, whether meals were eaten alone or with others, and television and computer use during meals. The ASA24 allows the respondent to add or modify food and drink choices at multiple points during the session, asks detailed questions about food preparation, portion size, and additions. Nutrition data are automatically analyzed to produce individual-level nutrients and food group estimates based on the Food and Nutrient Database for Dietary Surveys version 4.1, the corresponding MyPyramid Equivalents Database from USDA.

Additionally, in the second and third trimester questionnaires, questions pertaining to frequency of consumption of foods known to be high in chemical exposures of interest (e.g. metals) including rice, fish, and shellfish, as well as frequency of consumption of sugary and high- caloric foods, prenatal vitamins and supplements are also administered.

##### Infant dietary assessment

Infant dietary assessment through 12 months of age is primarily focused on breastfeeding initiation and duration as well as infant feeding practices including introduction to solids, using questions from the Infant Feeding Practices Study II [25]. Relevant timepoint-specific questions are asked at the 7–14 postnatal day timepoint, as well as at 3, 6, and 12 months postbirth. In addition to specific dietary intake questions, at 6 and 12 months after birth a 2-item screener to assess risk for food insecurity is also administered to mothers [26].

##### Other covariates

Information on maternal race/ethnicity, age at enrollment, education, household income, parity, pre-pregnancy weight and physical activity, family health history and country of origin, as well as information on paternal race/ethnicity, age, education, estimated height and weight and country of origin are assessed in prenatal interviewer-administered questionnaires with the mother.

#### Medical records abstraction

Additional information on maternal prenatal health including gestational diabetes diagnoses, clinical glucose challenge assessments, and measurements of height, weight, blood pressure and other vital data from prenatal clinical visits are abstracted from maternal prenatal records from healthcare providers via signed HIPAA authorization at study entry. Infant birth weight and delivery outcomes, infant growth, immunization history, and history of infections are abstracted from hospital birth records as well as from sick- and well-child visit records obtained from healthcare providers via maternal-signed HIPAA authorization at study entry for the child participant.

#### Biospecimen collection overview

MADRES participants provide multiple biospecimens during pregnancy, at birth and during the child’s infancy and childhood. Some of these biospecimens are stored for future studies and some are assayed as part of ongoing protocols as biomarkers of health status (e.g., salivary cortisol as a biomarker of the stress response) or biomarkers of exposure to environmental contaminants (e.g. hair and urine metals exposure). Table 2 lists the schedule of biospecimen collection for pregnancy through the child’s first year of life.

#### Biospecimen collection during pregnancy

##### Maternal fasting blood

Up to 50 ml of blood is collected during the initial study visit (for those enrolling prior to 20 weeks) and again at the third trimester study visit. Maternal blood is collected by a trained study staff phlebotomist using standard venipuncture protocols. Samples are transported to the laboratory within one hour using a duo-temperature cooler and ice packs. Serum, plasma, whole blood, red blood cells, and extracted DNA and RNA are stored at − 80 °C.

##### Maternal hair and nail clippings

Maternal hair and fingernail and/or toenail samples are collected in the initial (for those enrolling prior to 20 weeks) and third trimester visit. Nail clippings are collected from all 10 toes and/or fingers. Hair (~ 50 strands) is clipped from the base of the skull. Hair and nails are stored in labeled paper envelopes at − 80 °C.

##### Maternal urine

Maternal urine samples are collected during the initial (for those enrolling prior to 20 weeks) and third trimester study visits. Participants urinate in a sterile 90-ml urine specimen container and 1.5 ml aliquots and 10 ml aliquots are stored at − 80 °C in 2 ml and 15 ml cryovials, respectively.

##### Maternal stool

Maternal stool sample smears are collected at home prior to the initial study visit on fecal occult blood test (FOBT) cards. Study staff collect the cards at the initial visit and samples are stored at − 80 °C.

##### Maternal saliva

All mothers self-collect four saliva samples during a single day at home within one week of their scheduled third trimester study visits with the Salivette device (Sarstedtf, Inc.)—a small, cotton dental roll that participants insert into their mouths for 2 min, using established methods [27–32]. Study staff mail a saliva collection kit and participants are instructed to collect samples (1) immediately upon waking, (2) 30 min after waking, (3) between 3:00–4:00 pm, and (4) immediately before bedtime. To avoid contamination, saliva collection is completed before meals. Participants record date and time on the saliva tubes and store collected saliva samples in their home refrigerator as soon as possible after collection. Study staff collect the samples at the third trimester study visit. Once received, the collected samples are stored at − 80 °C.

#### Biospecimen collection at birth

For a subset of participants, umbilical cord blood, a newborn blood spot and placenta tissue are collected at delivery at partnering hospitals, processed, and stored for future analysis.

##### Umbilical cord blood (*UCB*)

Up to 50 ml of umbilical cord blood is collected at delivery after any clinical samples have been drawn and the sample is transported by study staff or via courier to the lab for initial processing, where plasma, serum, red blood cells, RNA/miRNA and DNA are isolated according to standard protocols and stored at − 80 °C.

##### Newborn blood spot (*NBS*)

An additional NBS is collected by the hospital phlebotomist on a trace metals-free blood spot card at the same time that a NBS sample is collected for the California Newborn Screening Program (usually on neonatal day 1 or 2) at one participating hospital. The NBS is placed in a sample bag and stored at − 80 °C.

##### Placenta tissue

Placenta specimens are collected by labor and delivery hosptital staff and stored at 4 °C on the labor and delivery floor at one participating hospital. Samples are retrieved within 48 h of delivery and brought to the lab for dissection. Briefly, 12 dime-sized samples are biopsied from the maternal side of the placenta in a clockwise fashion in an approximately 2 cm radius from the umbilical cord site. Dissected samples are stablized in RNAlater and then frozen at − 80 °C until analysis. Another 36 dime-sized samples are biopsied from the maternal side of the placenta in a clockwise fashion and stored in 50 ml tubes at − 80 °C. The remaining specimen is discarded.

#### Biospecimen collection at postpartum/postnatal visits

##### Maternal and infant hair and nail clippings

Maternal hair and toenail and/or fingernail samples are collected at 12 months after birth and stored as described for prenatal timepoints.

##### Maternal fasting blood

Maternal fasting blood is collected and stored at 12 months as described for prenatal timepoints.

##### Maternal urine

Maternal urine samples are collected at the 1-month and 12-month study visits as described for prenatal timepoints.

##### Infant stool

Infant stool samples are collected from a soiled diaper by study staff at the 1-month and 12-month study visits (or at home prior to the visits by the parent) and, using a wooden stick, smears are transferred to the corresponding sampling windows on the FOBT cards. Each FOBT card is then allowed to dry inside a small plastic bag containing a desiccant pack for up to two hours. The desiccant pack is then discarded and FOBT card is stored at − 80 °C.

### Environmental exposure assessment

#### Participant time-activity patterns, residential and occupational history

We are using a micro- environmental approach to estimate individual exposures to air pollutants and other environmental exposures that accounts for variation in time-activity patterns. To inform such an approach, information on occupational history and location, including indoor vs. outdoor work, is recorded from one year prior to pregnancy and throughout the study period. We assess commuting patterns throughout pregnancy via questionnaire by asking about each subject’s typical commute times, time of day and route. Time spent at home, at work and in commute are being used to time-weight individual pollution exposure estimates.

Residential (primary and secondary) and occupational addresses and occupancy dates of the pregnant mothers and their children from one year prior to conception through the third trimester of pregnancy are completed at home prior to the third trimester visit and then subsequently discussed with study staff at the visit to ensure data are captured accurately. Beginning in the third trimester, address data are collected prospectively at each study timepoint. Addresses are geocoded using Google Maps or comparable geocoding services and daily residential timelines are assembled for each participant starting two years prior to birth until the latest follow-up contact point to capture residential mobility and enable assignment of time-varying environmental exposures, especially in the gestational period.

#### Participant-reported housing characteristics, exposures and lifestyle factors

Extensive questionnaire data on indoor and outdoor exposures, including mode of travel to work or recreation activities; housing characteristics, such as type of dwelling and building age, carpeting inside the home, air conditioning presence and use, and gas stove use; pesticide usage indoors and outdoors; exposure to indoor and outdoor allergens, including pets (dog, cat) and pests (cockroach, rodent); history of tobacco smoke exposure (lifetime, prenatal, trimester-specific, and second-hand both during and after pregnancy) are collected throughout the study. Housing characteristics are updated for each documented address change throughout the study.

#### Local traffic and geospatial exposures assignment

Geographic Information Systems (GIS) tools and dispersion models are used to assign local traffic pollutant and geospatial exposures at the timeline of geocoded addresses for each subject. The local geophysical characteristics surrounding each point location include the distance to major roads and freeways, and the lengths of roads, traffic densities, percent impervious land surface, and monthly normalized difference vegetation index (NDVI) within defined buffers around residences and work locations. Near-roadway exposure to traffic air pollutants are characterized using the CALINE4 air quality dispersion model that incorporates roadway geometry, vehicle counts, vehicle emission rates, and atmospheric transport and dispersion (using wind speed, wind direction, atmospheric stability, and height of the mixing layer) [33, 34]. The advantage of the traffic dispersion-modeled estimates is that they reflect only the near-roadway component of exposure (although they are strong predictors of *local* spatial variations in measured ambient concentrations of elemental carbon and NO2 in southern California). The weekly and monthly traffic pollutant estimates show strong seasonal differences that can also help identify any trimester-specific effects [35–37].

#### Regional air pollution

Daily regional pollutant estimates of NO_2_, PM_10_, PM_2.5_ and ozone (O_3_) are assigned to the locations and gestational and early life time periods relevant for each study subject, using inverse-distance weighted spatial interpolation from ambient air quality monitoring data (United States Environmental Protection Agency Air Quality System) and a time-weighting approach for subjects with multiple residences or occupations during a time period.

#### Spatiotemporal air pollution exposure models

We have developed spatiotemporal models that can reliably predict concentrations with a high spatiotemporal resolution over long time spans in California. Leveraging the spatially extensive highly-clustered exposure data from short-term measurement campaigns and long-term routine ambient monitoring data from the U.S. EPA’s Air Quality System, we developed ensemble-learning based spatiotemporal exposure models that output uncertainty estimates and incorporate non-linear and spatial effects to reduce bias. For the NO_x_ model [38], important predictors include temporal basis functions, meteorological parameters, CALINE4 NO_x_, other local covariates described above, population density, and sub-county-level mean pollutant concentrations. The PM_2.5_ model uses similar predictors along with the MERRA-2 GMI Replay Simulation data assimilation product [39] that incorporates a global chemical transport model (~ 50 km spatial and hourly temporal resolution) and the MAIAIC daily 1x1km aerosol optical depth [40] derived from MODIS satellite. Ensemble learning and constrained optimization were used to enhance reliability of estimation over California and over a long period of time. The ensemble predictions of biweekly concentrations resulted in an R^2^ of 0.85 (RMSE: 4.7 ppb) for NO_2_ and 0.86 (RMSE: 13.4 ppb) for NO_x_. Ensemble learning and constrained optimization generated stable time series, which notably improved the results compared with those from initial mixed-effects models.

#### CalEnviroScreen (CES) 3.0

The Office of Environmental Health Hazards Assessment (OEHHA) of the California Environmental Protection Agency has developed a tool for evaluating multiple pollutants and stressors in communities, called the CalEnviroScreen (CES) 3.0 [41]. The model incorporates seven exposure indicators, five environmental effect indicators, three sensitive population indicators, and five socioeconomic indicators into a composite pollution burden subscore, a population subscore, and an overall CES score to characterize the cumulative exposure and rank each census tract. Residential locations of study participants are linked to the individual and composite CES indicators.

#### Social, demographic and built environment geospatial exposures

The following neighborhood-level parameters are assigned to residential locations using geospatial data layers in GIS mainly at the census tract or block group level as available: Walkability (EPA Smart Locations Database), greenness (Normalized Difference Vegetation Index, United States Department of Agriculture, USDA), access to parks and open spaces (Los Angeles County GIS Data Portal), food deserts and access to healthy food supermarkets and grocery stores (USDA Food Atlas), crime rates (Los Angeles County GIS Data Portal) and census-based demographic and socioeconomics variables (American Community Survey, e.g., racial composition, percent below poverty, percent home ownership, etc.).

### Biological stress assessment

Biological stress response, or assessment of dysregulated hypothalamic-pituitary-adrenal (HPA) axis activity, is assessed by measuring cortisol concentrations in saliva throughout the day. Cortisol concentrations from each of four home-collected saliva samples across a single day prior to the third trimester study visit are assayed with commercial chemiluminescence immunoassay (CLIA; IBL International, Hamburg, Germany), which has a lower detection limit of .005 μg/dL and intra- and inter-assay coefficients in the range of 3.0–4.1%.

## Baseline characteristics of subject enrolled to date

Between November 2015 and October 2018, 523 women with a gestational age (GA) less than 30 weeks were recruited from partner clinics participating in the MADRES cohort and enrolled in the study. A total of 408 women were enrolled prior to 20 weeks gestation (mean GA at study entry = 11.1 ± 4.0 weeks) and an additional 115 were enrolled between 20 and 30 weeks gestation (mean GA at study entry = 24.5 ± 2.8 weeks). After study enrollment, 33 became ineligible due to pregnancy loss (*N* = 31) or subsequent determination of multiple gestation (*N* = 2), and 20 women dropped out of the study after enrollment.

**Fig. 1:**
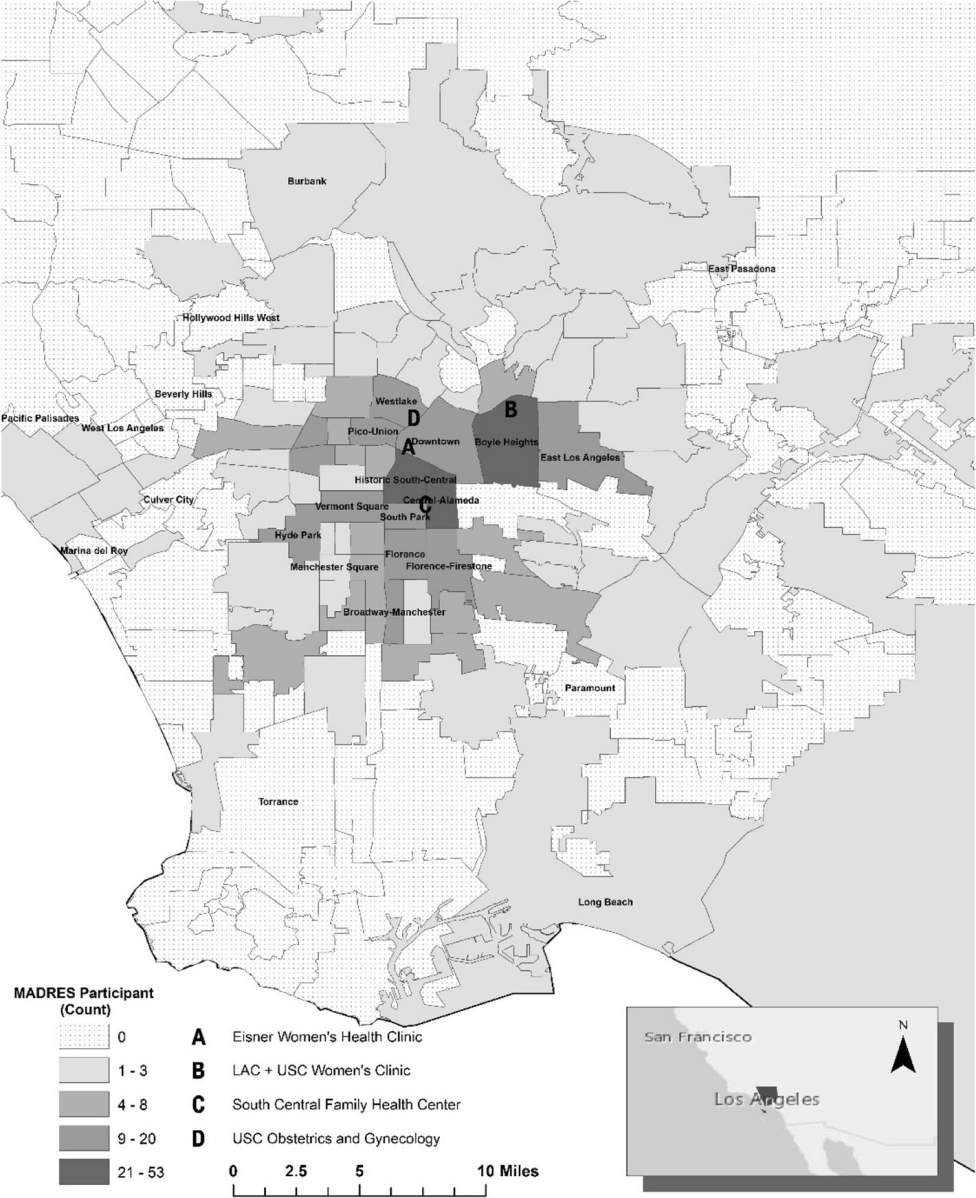
MADRES Participant Neighborhoods at Recruitment During Pregnancy

Figure 1 shows geographical residential neighborhoods of 462 MADRES participants with with geocoded recruitment addresses. The clinical recruitment sites are located in central, east and south Los Angeles where the majority of participant residences also were reported at study entry; however, there is a wide geographic distribution of participants’ residences in Southern California.

**Fig. 2:**
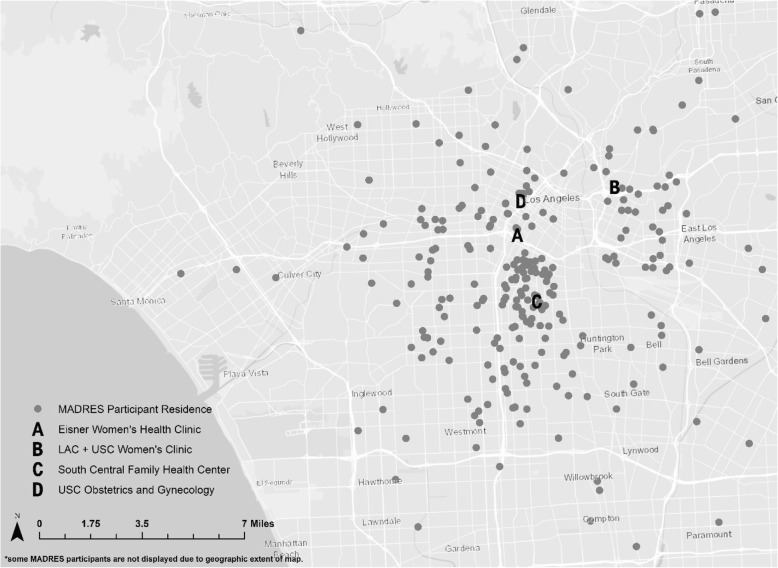
MADRES Recruitment Centers and Participants’ Birth Residences

As of October 1, 2018, 291 women had delivered. The geographical distribution of residential locations of women who resided in Los Angeles County at delivery is presented in Fig. 2. Participant demographic characteristics at study entry are shown in Table 3. Overall, the participants in the MADRES cohort who have already delivered are predominantly Hispanic (74.23%), with 7.56% non-Hispanic white, 1.72% non-Hispanic multi-race, 9.97% non-Hispanic black, and 3.44% non-Hispanic other races. Approximately 53% of participants reported that they were born outside of the US or Canada, with the most common non-US/Canada birthplaces being Mexico (26%) and Central America (11%). Over 65% reported that they were married or living with a partner at study entry. Fifty-two percent of participants reported an annual household income of less than $30,000 and 54% reported an education level at or below grade 12.

**Table 3:** Selected Characteristics at Study Entry of MADRES Study Participants Who Had Delivered As of October 1, 2018 (N=291)

	n(%) or Mean (SD)
Cohort Entry Point	
<20 weeks (Regular Entry)	220 (75.6)
20-30 weeks (Late Entry)	71 (24.4)
Maternal Age (years)	
<=20 years	30 (10.3)
21-25 years	63 (21.7)
26-30 years	83 (28.5)
31-35 years	85 (29.2)
36-40 years	23 (7.9)
> 40 years	7 (2.4)
Gestational Age (weeks)	
<20 weeks (Regular Entry)	11.26 (4.0)
20-30 weeks (Late Entry)	24.99 (2.9)
Race and Ethnicity	
Hispanic	216 (74.2)
Black, Non-Hispanic	29 (9.9)
Multi-racial, Non-Hispanic	5 (1.7)
Other, Non-Hispanic	10 (3.4)
White, Non-Hispanic	22 (7.6)
Missing / Refused to Answer	9 (3.1)
Place of Birth	
US or Canada	138 (47.4)
Mexico	76 (26.1)
Other Central America	32 (11.0)
South America	1 (0.3)
Asia	9 (3.1)
Europe	3 (1.0)
Australia	1 (0.3)
Missing / Refused to Answer	31 (10.7)
Education	
< 12th grade	74 (25.4)
Completed grade 12	83 (28.5)
Some college or tech school	75 (25.8)
Completed college	29 (10.0)
Some graduate training	20 (6.9)
Missing / Refused to Answer	10 (3.4)
Household Income	
Less than $15,000	69 (23.7)
$15,000 to $29,999	83 (28.5)
$30,000 to $49,999	36 (12.4)
$50,000 to $99,999	18 (6.2)
$100,000 or more	21 (7.2)
Don't know	55 (18.9)
Missing / Refused to Answer	9 (3.1)
Employment	
Homemaker	70 (24.1)
Student	14 (4.8)
Employed	119 (40.9)
Temporary Medical Leave	5 (1.7)
Unemployed	37 (12.7)
Missing / Refused to Answer	46 (15.8)
Marital Status	
Married	77 (26.5)
Living together	114 (39.2)
Never married, single	58 (19.9)
Divorced or separated	6 (2.1)
Missing / Refused to Answer	36 (12.4)

Table 4 shows health characteristics of MADRES participants during their pregnancies. Approximately 38% of women reported that the index pregnancy was their first child, while 28% reported 3 or more previous births. Twenty-four percent of participants reported pre-pregnancy weight and height meeting CDC body mass index (BMI) criteria for overweight (BMI > 25 and < 30 kg/m^2^) and 25% of participants reported pre-pregnancy weight and height meeting CDC BMI Class I-III criteria for obesity (BMI > 30 kg/m^2^). Over 40% of women reported pre-prenancy physical activity on 3 or fewer days per week. A diagnosis of gestational diabetes was made in 6% of women whose records were abstracted (14 of 231 records abstracted to date). In addition, over 15% of women reported a lifetime physician-diagnosis of asthma and over 26% reported a lifetime history of any allergies. Seventy percent of women reported never smoking and 90% reported no exposure to secondhand smoke during the index pregnancy.

**Table 4:** Health Characteristics of MADRES Study Participants Who Had Delivered As of October 1, 2018 (*n* = 291)

	n(%) or mean (SD)
Pre-Pregnancy BMI, kg/m^2^	
Underweight (BMI < 18.5)	6 (2.1)
Normal (BMI of 18.5 to < 25)	80 (27.5)
Overweight (BMI of 25 to < 30)	70 (24.1)
Class 1 obese (BMI of 30 to < 35)	46 (15.8)
Class 2 obese (BMI of 35 to < 40)	18 (6.2)
Class 3 obese (BMI > = 40)	9 (3.1)
Missing / Refused to Answer	62 (21.3)
Pre-Pregnancy Physical Activity	
0 days per week	30 (10.3)
1–3 days per week	87 (29.9)
4–7 days per week	142 (48.8)
Missing / Refused to Answer	32 (11.0)
Gestational Diabetes Diagnosis (abstracted)	
Yes	14 (4.8)
No	217 (74.6)
Missing	60 (20.6)
History of Physician Diagnosis of Asthma	
Yes	46 (15.8)
No	235 (80.8)
Missing / Refused to Answer	10 (3.4)
History of Allergies	
Yes	78 (26.8)
No	179 (61.5)
Missing / Refused to Answer	34 (11.7)
History of Personal Smoking	
Ever	76 (26.1)
Never	205 (70.4)
Missing / Refused to Answer	10 (3.4)
Second Hand Smoke Exposure in Pregnancy	
Yes	19 (6.5)
No	262 (90.0)
Missing / Refused to Answer	10 (3.4)

Table 5 shows characteristics of MADRES infant participants at birth. Infants on average were born full term at 38.9 weeks gestation with a mean birthweight of 3239 ± 567 g. Over 11% (*n* = 34) of infants were born pre-term (defined as < 37 weeks gestation). Eight percent of infants were defined as small for gestational age (SGA) and 4% of infants were defined as large for gestational age (LGA), based on Fenton growth charts [14].

**Table 5:** Characteristics of MADRES Infants Who Were Born as of October 1, 2018 (n = 291)

	n(%) or mean (SD)
Gestational Age at Birth, weeks	38.97 (2.2)
Infant Birthweight, grams	3250.98 (592.1)
Low Birthweight (< 2500 g)	14 (4.8)
Fenton Category	
Small for Gestational Age	22 (7.6)
Appropriate for Gestational Age	224 (77.0)
Large for Gestational Age	12 (4.1)
Missing	33 (11.3)
Sex	
Female	139 (47.8)
Male	143 (49.1)
Missing	9 (3.1)

## Discussion

Childhood obesity and its later life consequences have become major public health and clinical problems that urgently need effective prevention strategies. Eliminating the racial/ethnic disparities in obesity is also a national priority, given the numerous associated adverse cardio-metabolic outcomes [7]. We outlined the study rationale and data collection protocol for the MADRES pregnancy cohort, a prospective pregnancy cohort with a target of 1000 predominantly lower-income, Hispanic women in Los Angeles, CA. We presented a profile of 291 participants who have delivered their infants, out of 523 participants enrolled in the study from November 2015 to October 2018 from four community prenatal care providers in Los Angeles. We will prospectively examine how environmental and psychosocial determinants affect overall early childhood growth trajectories as well as maternal gestational weight gain and postpartum weight retention among minority and low-income children and mothers, respectively.

While some recent studies have suggested that childhood obesity rates have stabilized overall and even decreased among the youngest children [42–45], a recently-published paper using NHANES data for years 1999 to 2016 suggests a strong increasing trend in overweight and obesity among children age 2–19 and a sharp increase in obesity prevalence in children age 2–5 compared to previous cycles [4].

Childhood obesity disproportionally affects Hispanic and African-American populations [2, 3]. Prevalence rates of obesity among the Hispanic population exceed 21% nationally and 29% in California, compared to 15 and 21%, respectively, in non-Hispanic whites [3, 46, 47]. This obesity disparity is already present by preschool age, suggesting that it may have its origins in the earliest stages of life. Pregnancy-related obesity rates are also disproportionately high in minority women [5, 6]. Among U.S. Hispanic women of childbearing age, 40% are obese as compared with only 31% of non-Hispanic white women [43] and up to 51% gain excessive weight during pregnancy [6, 48]. In our MADRES population, the prevalence of pre-pregnancy maternal obesity is approximately 37% among Hispanic participants compared to 14% of non-Hispanic white participants (data not shown). The prevalence of pre-pregnancy maternal overweight plus obesity among Hispanic MADRES participants is over 70% compared to 36% among non-Hispanic white MADRES participants (data not shown).

Given the multiple adverse effects of excessive weight gain on maternal and child health outcomes [49, 50], the Institute of Medicine (IOM) has recommended limiting pregnancy-related weight gain in obese women to 4.99–9.07 kg; however, more than half of obese women gain weight in excess of this recommendation during pregnancy [51]. A recent study has suggested that obese women with excessive gestational weight gain show the greatest alterations in circadian cortisol and their children may be at greatest risk for adverse child health outcomes [52]. Data from the MADRES cohort will be able to address whether excessive gestational weight again among Hispanic and non-Hispanic women who are obese, overweight or normal weight contributes to altered cortisol patterns and psychosocial stress levels as well as leads to poorer health outcomes in children.

Social environmental stressors both at the neighborhood and individual level have also been associated with increased obesity risk. Lower perceived neighborhood crime safety was linked to higher BMI and greater risk of obesity in a study of low-income adults [53]. Exposure to individual-level social stressors such as interpersonal violence (e.g., physical/sexual abuse, domestic abuse) has been shown to increase the risk of obesity [54]. Experiencing physical and/or sexual violence has also been associated with abdominal obesity, low high-density lipoprotein cholesterol, and elevated triglycerides [55]. Other individual-level social stressors such as negative life events (e.g., death of a loved one, divorce, unemployment) were related to increased risk of overweight and obesity [56–59].

In addition to psychosocial and biological stress response markers, the relationship between physical environmental stressors and health outcomes are being assessed in the MADRES cohort. Ambient air pollution is likely an important environmental contributor to the rise in obesity prevalence [13]. Multiple direct mechanisms of air pollution effects have been investigated, including impacts on metabolic function through oxidative stress pathways and adipose tissue inflammation [60], or indirectly through decreased physical activity on higher air pollution days [61]. We have shown that prenatal and early-life exposures to traffic-related air pollution are associated with early childhood obesity, growth trajectory of body mass index (BMI), and attained BMI at age 18 years in the Children’s Health Study (CHS) [62, 63]. A longitudinal pregnancy cohort study in Northern Manhattan has also shown that higher personal prenatal polycyclic aromatic hydrocarbon (PAH) exposure during pregnancy was associated with higher BMI in children at age 5 [64]. Many of these associations may be modified by other known risk factors for obesity, such as genetics, maternal obesity, psychosocial stress, and the built environment [65–69]. The MADRES cohort provides an opportunity to address many of these potential effect modifiers.

Intervention strategies targeted at individuals who are already obese have met with mixed success [70–73], and therefore, alternative intervention strategies aimed at *preventing* modifiable risk factors such as environmental chemical exposures as well as understanding psychological, behavioral or biological mechanisms that underlie the observed disparities in disease risk and exposure are urgently needed. The MADRES cohort is poised to address many critical gaps in understanding the etiologic contributions of environmental pollution to childhood and maternal obesity. Its strengths include: (1) a prospective pregnancy cohort design with multiple in-person and telephone visits with repeated biospecimen collection in both mothers and infants; (2) cutting-edge approaches for environmental exposure assessment and health outcomes assessment; (3) strong clinical partnerships with community-based healthcare providers with high proportions of medically underserved and research underserved populations; (4) a dedicated, bilingual, highly-trained staff team drawn from the target population communities who are flexible, considerate, and well-prepared to address the concerns of vulnerable populations.

The MADRES cohort is one of five Centers of Excellence on Environmental Health Disparities Research, a joint effort funded by the National Institute of Environmental Health Sciences, the National Institute of Minority Health and Health Disparities, and the Environmental Protection Agency, that encourages broad multi-disciplinary efforts to investigate diseases that are known to have a significant burden on low socioeconomic and health disparate populations who also have a significant burden of exposure to environmental pollutants and toxicants. The program supports research, mentoring, capacity building, and community engagement [74].

The MADRES cohort is also one of 84 cohort studies selected for the Environmental Influences on Child Health Outcomes (ECHO) Program, a large consortium of pregnancy and early childhood cohorts that is focused on understanding the impacts of the physical, chemical, biological, social, behavioral, natural and built environments on child health and development [75]. The ECHO Program will provide numerous opportunities for addressing critical gaps in these areas beyond the child’s first year of life and in understanding and preventing adverse health outcomes that may have lasting consequences into later adulthood.

In summary, results from the MADRES cohort could provide a powerful rationale for regulation of targeted chemical environmental components, better transportation and urban design policies, and clinical recommendations for stress-coping strategies and behavior to reduce lifelong obesity risk.

## Additional files

Additional file 1: MADRES Intake Questionnaire. Questionnaire administered at the time of recruitment collecting contact and next of kin information. (DOCX 33 kb)

Additional file 2: MADRES Intake Questionnaire_Spanish. Spanish questionnaire administered at the time of recruitment collecting contact and next of kin information. (DOC 98 kb)

Additional file 3: V1 Essential Questions. Questionnaire administered at the time of recruitment asking about maternal race, ethnicity, education and total household income. (DOCX 27 kb)

Additional file 4: V1 Essential Questions_Spanish. Spanish questionnaire administered at the time of recruitment asking about maternal race, ethnicity, education and total household income. (DOCX 31 kb)

Additional file 5: First Trimester Questionnaire. Questionnaire administered during the first study visit for participants recruited before 20 weeks of pregnancy. (DOC 340 kb)

Additional file 6: First Trimester Questionnaire_Spanish. Spanish questionnaire administered during the first study visit for participants recruited before 20 weeks of pregnancy. (DOC 370 kb)

Additional file 7: MADRES_PPAQ. Pregnancy physical activity questionnaire conducted at the first study visit and the third trimester study visit. (DOCX 16 kb)

Additional file 8: MADRES_PPAQ_Spanish. Spanish pregnancy physical activity questionnaire conducted at the first study visit and the third trimester study visit. (DOCX 17 kb)

Additional file 9: MADRES Second Trimester Questionnaire. Questionnaire administer during the second trimester of pregnancy. (DOCX 130 kb)

Additional file 10: MADRES Second Trimester Questionnaire_Spanish. Spanish questionnaire administered during the second trimester of pregnancy. (DOCX 158 kb)

Additional file 11: MADRES Third Trimester Questionnaire. Questionnaire administered during the third trimester visit to participants recruited before 20 weeks of pregnancy. (DOCX 132 kb)

Additional file 12: MADRES Third Trimester Questionnaire_Spanish. Spanish questionnaire administered during the third trimester visit to participants recruited before 20 weeks of pregnancy. (DOCX 159 kb)

Additional file 13: MADRES Late Entry Questionnaire. Questionnaire administered during the third trimester for participant recruited between 20 and 30 weeks of pregnancy. (DOCX 144 kb)

Additional file 14: MADRES Late Entry Questionnaire_Spanish. Spanish questionnaire administered during the third trimester for participant recruited between 20 and 30 weeks of pregnancy. (DOCX 173 kb)

Additional file 15: Occupational History Form. Occupational history questionnaire mailed to participants to complete and bring to the third trimester visit. (DOCX 37 kb)

Additional file 16: Occupational History Form_Spanish. Spanish occupational history questionnaire mailed to participants to complete and bring to the third trimester visit. (DOCX 38 kb)

Additional file 17: Residential History Form. Residential history questionnaire mailed to participants to complete and bring to the third trimester visit. (DOC 72 kb)

Additional file 18: Residential History Form_Spanish. Spanish residential history questionnaire mailed to participants to complete and bring to the third trimester visit. (DOC 73 kb)

Additional file 19: 7-14 Day Questionnaire. Questionnaire administered 7-14 days after child participant is born. (DOCX 42 kb)

Additional file 20: 7-14 Day Questionnaire_Spanish. Spanish questionnaire administered 7-14 days after child participant is born. (DOCX 43 kb)

Additional file 21: MADRES 1-Month Questionnaire. Questionnaire administered during the 1-month study visit. (DOCX 132 kb)

Additional file 22: MADRES 1-Month Questionnaire_Spanish. Spanish questionnaire administered during the 1-month study visit. (DOCX 112 kb)

Additional file 23: Three Month Post Birth Questionnaire. Questionnaire administered 3 months after child participant is born. (DOCX 155 kb)

Additional file 24: Three Month Post Birth Questionnaire_Spanish. Spanish questionnaire administered 3 months after child participant is born. (DOCX 160 kb)

Additional file 25: Six Month Post Birth Questionnaire. Questionnaire administered 3 months after child participant is born. (DOCX 159 kb)

Additional file 26: Six Month Post Birth Questionnaire_Spanish. Spanish questionnaire administered 6 months after child participant is born. (DOCX 163 kb)

Additional file 27: Twelve Month Post Birth Questionnaire. Questionnaire administered during the 1-year study visit. (DOCX 206 kb)

Additional file 28: 12 Month Post Birth Questionnaire_Spanish. Spanish questionnaire administered during the 1-year study visit. (DOCX 214 kb)

## Abbreviations

ACEs: Adverse Childhood Experiences
ASA24: Automated, Self-Administered 24-h Diet Recall
BMI: Body mass index
CALINE4: California line source dispersion model, version 4
CDC: Centers for Disease Control and Prevention
CES: CalEnviroScreen 3.0
CES-D: Center for Epidemiological Studies-Depression Scale
CHS: Children’s Health Study
ECHO: Environmental Influences on Child Health Outcomes (ECHO) Program
FOBT: Fecal Occult Blood Test
GA: Gestational age
GIS: Geographic Information Systems
HIPAA: Health Information Portability and Accountability Act
HPA: Hypothalamic-pituitary-adrenal axis
LBW: Low birth weight
LGA: Large for gestational age
MADRES: Maternal And Developmental Risks from Environmental and Social Stressors cohort study
Medi-Cal: California Medicaid Program
MODIS: Moderate Resolution Imagine Spectroradiometer satellite
NBS: Newborn Blood Spot
NDVI: Normalized difference vegetation index
NHANES: National Health and Nutrition Examination Survey
NO_2_: Nitrogen Dioxide
NOx: Nitrogen oxides
O_3_: Ozone
OEHHA: Office of Environmental Health Hazards Assessment of California EPA
PhenX: Consensus measures for **Phen**otypes and e**X**posures
PM_10_: Particulate Matter less than 10 μm in diameter
PM_2.5_: Particulate Matter less than 2.5 μm in diameter
PPAQ: Pregnancy Physical Activity Questionnaire
PPB: parts per Billion
PSS: Perceived Stress Scale
SGA: Small for gestational age
UCB: Umbilical Cord Blood
USC: University of Southern California
WIC: Women, Infants and Children
