# Supplementary material for: Study Design, Protocol and Profile of the Maternal And Developmental Risks from Environmental and Social Stressors (MADRES) Pregnancy Cohort: a Prospective Cohort Study in Predominantly Low-Income Hispanic Women in Urban Los Angeles
Source: BMC Pregnancy Childbirth. 2019 May 30;19:189. doi: 10.1186/s12884-019-2330-7 (PMC6543670; doi:10.1186/s12884-019-2330-7)
Supplement: Supplementary file 1 — MADRES Intake Questionnaire. Questionnaire administered at the time of recruitment collecting contact and next of kin information. (DOCX 33 kb) [file 12884_2019_2330_MOESM1_ESM.docx]

**MADRES Intake Questionnaire**

**Date: ___/___/____ Time: ______:______ AM /PM**

**Subject ID#:________________ Interviewer: ___________________________**

**CONTACT INFORMATION**

**1. Name:** _________________ _______________ ____________________ ____________________

First Middle Last 1 Last 2

**2. Other names used** (e.g. Maiden name) ­­­­­­­­­­­­­­­­­­­­­­­­­:­___________________________

**3**. **Your Date of Birth:** **_______/_______/_______**

Month Day Year

**4**. **Estimated** **Due Date:** **_______/_______/_______**

Month Day Year

**5.** **Last Menstrual Period:** **_______/_______/_______**

Month Day Year

**6. What is your cell phone number?** ____________________________

□₀ Don’t have a cell phone **(Skip to question #8)**

**7.** **Is this a prepaid cell phone or a permanent phone number?**

□₀ prepaid

□₁ permanent number

**8.** **What is your HOME address (the address at which you spend the most time)?**

Address: ______________________________________________________________________

City: ___________________________State: ________________Zip: _____________________

**9A. Please tell me the names of other adults living with you:**

Adult#1 First: ______________________Last: ______________________Middle: ______________

Relation to you: ___________________ Cell Phone: ______________________

Adult#2 First: ______________________Last: ______________________Middle: ______________

Relation to you: ___________________ Cell Phone: ______________________

Adult#3 First: ______________________Last: ______________________Middle: ______________

Relation to you: ___________________ Cell Phone: ______________________

**10. What is the phone number for the HOME listed in Question 8?** ____________________________

□₀ Don’t have a home phone

**11. Do you live at more than one home?**

□₁ Yes... *Complete questions 12A, 12B and 12C* □₀No… *Go to Question #13*

**12A. What is your second HOME address?**

Address: ______________________________________________________________________

City: ___________________________State: ________________Zip: _____________________

**12B. What is the phone number for the HOME listed in Question 12A?** _______________________

□₀ Don’t have a home phone

**12C. How much time do you spend at the address listed in 12A?**

 1%-25% of the time

 26%-50% of the time

**13. A. What is your email address?** _________________________ 0 ❑Don’t have an email address

**B. What is your Facebook username?** ___________________________0 ❑Don’t have Facebook

**C. What is your Twitter handle?** @________________________________0 ❑Don’t have Twitter

**D. What is your Instagram contact name?** _______________________0 ❑Don’t have Instagram

**14.** **A. How do you prefer to be contacted?**

 Phone

 Email

 Text

 Other: ________________

**B. What are the best days to reach you?**

 Monday

 Tuesday

 Wednesday

 Thursday

 Friday

 Saturday

 Sunday

**C. What are the best times to reach you (Monday)?**

 Mornings (8am-12pm)

 Afternoons (12pm-5pm)

 Evenings (5pm-8pm)

 Other: ________________

**D. What are the best times to reach you (Tuesday)?**

 Mornings (8am-12pm)

 Afternoons (12pm-5pm)

 Evenings (5pm-8pm)

 Other: ________________

**E. What are the best times to reach you (Wednesday)?**

 Mornings (8am-12pm)

 Afternoons (12pm-5pm)

 Evenings (5pm-8pm)

 Other: ________________

**F. What are the best times to reach you (Thursday)?**

 Mornings (8am-12pm)

 Afternoons (12pm-5pm)

 Evenings (5pm-8pm)

 Other: ________________

**G. What are the best times to reach you (Friday)?**

 Mornings (8am-12pm)

 Afternoons (12pm-5pm)

 Evenings (5pm-8pm)

 Other: ________________

**H. What are the best times to reach you (Saturday)?**

 Mornings (8am-12pm)

 Afternoons (12pm-5pm)

 Evenings (5pm-8pm)

 Other: ________________

**I. What are the best times to reach you (Sunday)?**

 Mornings (8am-12pm)

 Afternoons (12pm-5pm)

 Evenings (5pm-8pm)

 Other: ________________

**15. What is the baby’s father’s name?** □ Don’t know

_________________ _______________ ____________________ ____________________

First Middle Last 1 Last 2

**16A. Do you have a spouse/partner?** 0 ❑ No…Go to Question17 1 ❑ Yes

**16B. What is the name of your spouse/partner**? □ Same as above □ No spouse/partner

_________________ _______________ ____________________ ____________________

First Middle Last 1 Last 2

**17.** **In order to help locate you in case you move and/or change your phone number can you provide us with contact information for your mother and three friends/family members not living with you who would be able to provide us with your new contact information?**

YOUR MOTHER’S INFORMATION

First: ______________________Last: ______________________Middle: ______________

Address: ______________________________________________________________________

City: ___________________________State: ________________Zip: _____________________

Cell Phone: ______________________ Home Phone: ______________________

NOK#1

First: ______________________Last: ______________________Middle: ______________

Relation to you: ___________________Email address: ____________________________

Cell Phone: ______________________ Home Phone: ______________________

NOK#2

First: ______________________Last: ______________________Middle: ______________

Relation to you: ___________________Email address: ____________________________

Cell Phone: ______________________ Home Phone: ______________________

NOK#3

First: ______________________Last: ______________________Middle: ______________

Relation to you: ___________________Email address: ____________________________

Cell Phone: ______________________ Home Phone: _______________________________

**MAILING ADDRESS**

**18. Do you have a P.O. Box or a mailing address that is different than your home address?**

0 ❑ No

1 ❑ Yes…what is your P.O. Box or mailing address?

Address: _________________________________________________________________

City: ___________________________State: ________________Zip: ________________
