# Supplementary material for: Study Design, Protocol and Profile of the Maternal And Developmental Risks from Environmental and Social Stressors (MADRES) Pregnancy Cohort: a Prospective Cohort Study in Predominantly Low-Income Hispanic Women in Urban Los Angeles
Source: BMC Pregnancy Childbirth. 2019 May 30;19:189. doi: 10.1186/s12884-019-2330-7 (PMC6543670; doi:10.1186/s12884-019-2330-7)
Supplement: Supplementary file 3 — V1 Essential Questions. Questionnaire administered at the time of recruitment asking about maternal race, ethnicity, education and total household income. (DOCX 27 kb) [file 12884_2019_2330_MOESM3_ESM.docx]

**MADRES V1 Essential Questions**

**DEMOGRAPHIC DATA**

**1**. Are you of Hispanic or Latino ethnicity?

0 ❑ No

1 ❑ Yes

**2.** What is your race? (**MARK ALL THAT APPLY**):

1 ❑ White

2 ❑ Asian

3 ❑ Black or African-American

4 ❑ Native Hawaiian or Pacific Islander

5 ❑ American Indian/Alaska Native

6 ❑ Other: Explain: _________________

**3.** What was the last grade in school **you** completed? **[MARK ONE]**

1 ❑ Less than 12th grade (did not finish high school)

2 ❑ Completed grade 12 (high school)

3 ❑ Some college or technical school

4 ❑ Completed 4 years of college

5 ❑ Some graduate training after college

**INCOME**

**4.** In which of the following categories did your TOTAL HOUSEHOLD FAMILY INCOME fall in last year? Include all incomes, before taxes and deductions, of all members of the family.

_1_ ❑ Less than $15,000 _4_ ❑ $50,000 to $99,999

_2_ ❑ $15,000 to $29,999 _5_ ❑ $100,000 or more

_3_ ❑ $30,000 to $49,999 _9_ ❑ Don’t know
