# Supplementary material for: Study Design, Protocol and Profile of the Maternal And Developmental Risks from Environmental and Social Stressors (MADRES) Pregnancy Cohort: a Prospective Cohort Study in Predominantly Low-Income Hispanic Women in Urban Los Angeles
Source: BMC Pregnancy Childbirth. 2019 May 30;19:189. doi: 10.1186/s12884-019-2330-7 (PMC6543670; doi:10.1186/s12884-019-2330-7)
Supplement: Supplementary file 4 — V1 Essential Questions_Spanish. Spanish questionnaire administered at the time of recruitment asking about maternal race, ethnicity, education and total household income. (DOCX 31 kb) [file 12884_2019_2330_MOESM4_ESM.docx]

**MADRES V1 Essential Questions**

**INFORMACION DEMOGRAPHICA**

**1**. ¿Es usted de descendencia hispana o latina?

0 ❑ No

1 ❑ Sí

**2.** ¿Cuál es su origen racial? (**MARK ALL THAT APPLY**):

1 ❑ Blanco

2 ❑ Asiático

3 ❑ Negro o Afroamericano

4 ❑ De origen nativo Hawaiano o de las Islas del Pacifico

5 ❑ Indio Americano o nativo de Alaska

6 ❑ Otro: Explique: _________________

**3.** ¿Cuál es el último grado escolar que **usted** completó? **[MARK ONE]**

1 ❑ Menos de grado 12 (no terminé la secundaria)

2 ❑ Terminé el grado 12 (terminé la secundaria)

3 ❑ Asistí a la universidad o a una escuela técnica

4 ❑ Completé cuatro años en la universidad

5 ❑ Tengo educación de posgrado después de completar la universidad

**INGRESOS**

**4.** ¿En cuál de las siguientes categorías se encuentra el TOTAL DE INGRESOS FAMILIARES durante el último año? Incluya todos los ingresos antes de impuestos y deducciones de todos los miembros de su familia.

_1_ ❑ Menos de $15,000

_2_ ❑ $15,000 to $29,999

_3_ ❑ $30,000 to $49,999

_4_ ❑ $50,000 to $99,999

_5_ ❑ $100,000 o más

_9_ ❑ No sé
