# Supplementary material for: Study Design, Protocol and Profile of the Maternal And Developmental Risks from Environmental and Social Stressors (MADRES) Pregnancy Cohort: a Prospective Cohort Study in Predominantly Low-Income Hispanic Women in Urban Los Angeles
Source: BMC Pregnancy Childbirth. 2019 May 30;19:189. doi: 10.1186/s12884-019-2330-7 (PMC6543670; doi:10.1186/s12884-019-2330-7)
Supplement: Supplementary file 5 — First Trimester Questionnaire. Questionnaire administered during the first study visit for participants recruited before 20 weeks of pregnancy. (DOC 340 kb) [file 12884_2019_2330_MOESM5_ESM.doc]

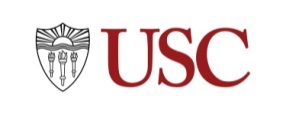
 **MADRES Study**

**Today’s Date:** _____________________ **Interviewer Name:** ____________________

**Instructions:** Thank you for agreeing to participate in this study. In this interview, I will be asking some questions about you and your health history. Please answer all questions as best as you can, even if you are not completely certain. Be assured that your answers are confidential. Please feel free to interrupt me and ask about anything that is not clear**.**

**CONTACT INFORMATION**

**1. Name:** _________________ _______________ ____________________ ____________________

First Middle Last 1 Last 2

**2. Other names used** (e.g. Maiden name) ­­­­­­­­­­­­­­­­­­­­­­­­­:­___________________________

### **3**. **Your Date of Birth:** **_______/_______/_______**

Month Day Year

### **4**. **Estimated** **Due Date:** **_______/_______/_______**

Month Day Year

### **5.** **Last Menstrual Period:** **_______/_______/_______**

Month Day Year

**6. What is your cell phone number?** ____________________________

□₀ Don’t have a cell phone **(Skip to question #8)**

**7.** **Is this a prepaid cell phone or a permanent phone number?**

□₀ prepaid

□₁ permanent number

**8.** **What is your HOME address (the address at which you spend the most time)?**

Address: ______________________________________________________________________

City: ___________________________State: ________________Zip: _____________________

**8A.** **If moved…When did you move into your new home address?** _______________________

**9A. Please tell me the names of other adults living with you:**

Adult#1 First: ______________________Last: ______________________Middle: ______________

Relation to you: ___________________ Cell Phone: ______________________

Adult#2 First: ______________________Last: ______________________Middle: ______________

Relation to you: ___________________ Cell Phone: ______________________

Adult#3 First: ______________________Last: ______________________Middle: ______________

Relation to you: ___________________ Cell Phone: ______________________

**10. What is the phone number for the HOME listed in Question 8?** ___________________________

□₀ Don’t have a home phone

**11. Do you live at more than one home?**

□₁ Yes... *Complete questions 12A, 12B and 12C* □₀No… *Go to Question #13*

**12A. What is your second HOME address?**

Address: ______________________________________________________________________

City: ___________________________State: ________________Zip: _____________________

**12A2.** **If moved…When did you move into your new second home address?**  ___________________

**12B. What is the phone number for the HOME listed in Question 12A?** _______________________

□₀ Don’t have a home phone

**12C. How much time do you spend at the address listed in 12A?**

 1%-25% of the time

 26%-50% of the time

**13. A. What is your email address?** _________________________ 0 ❑Don’t have an email address

**B. What is your Facebook username?** ___________________________0 ❑Don’t have Facebook

**C. What is your Twitter handle?** @________________________________0 ❑Don’t have Twitter

**D. What is your Instagram contact name?** _______________________0 ❑Don’t have Instagram

**14.** **A. How do you prefer to be contacted?**

 Phone

 Email

 Text

 Other: ________________

**B. What are the best days to reach you?**

 Monday

 Tuesday

 Wednesday

 Thursday

 Friday

 Saturday

 Sunday

**C. What are the best times to reach you (Monday)?**

 Mornings (8am-12pm)

 Afternoons (12pm-5pm)

 Evenings (5pm-8pm)

 Other: ________________

**D. What are the best times to reach you (Tuesday)?**

 Mornings (8am-12pm)

 Afternoons (12pm-5pm)

 Evenings (5pm-8pm)

 Other: ________________

**E. What are the best times to reach you (Wednesday)?**

 Mornings (8am-12pm)

 Afternoons (12pm-5pm)

 Evenings (5pm-8pm)

 Other: ________________

**F. What are the best times to reach you (Thursday)?**

 Mornings (8am-12pm)

 Afternoons (12pm-5pm)

 Evenings (5pm-8pm)

 Other: ________________

**G. What are the best times to reach you (Friday)?**

 Mornings (8am-12pm)

 Afternoons (12pm-5pm)

 Evenings (5pm-8pm)

 Other: ________________

**H. What are the best times to reach you (Saturday)?**

 Mornings (8am-12pm)

 Afternoons (12pm-5pm)

 Evenings (5pm-8pm)

 Other: ________________

**I. What are the best times to reach you (Sunday)?**

 Mornings (8am-12pm)

 Afternoons (12pm-5pm)

 Evenings (5pm-8pm)

 Other: ________________

**15.** **What is the baby’s father’s name?** □ Don’t know

_________________ _______________ ____________________ ____________________

First Middle Last 1 Last 2

**16A. Do you have a spouse/partner?** 0 ❑ No…Go to Question 17 1 ❑ Yes

**16B. What is the name of your spouse/partner**? □ Same as above □ No spouse/partner

_________________ _______________ ____________________ ____________________

First Middle Last 1 Last 2

**17.** **In order to help locate you in case you move and/or change your phone number can you provide us with contact information for your mother and three friends/family members not living with you who would be able to provide us with your new contact information?**

YOUR MOTHER’S INFORMATION

First: ______________________Last: ______________________Middle: ______________

Address: ______________________________________________________________________

City: ___________________________State: ________________Zip: _____________________

Cell Phone: ______________________ Home Phone: ______________________

NOK#1

First: ______________________Last: ______________________Middle: ______________

Relation to you: ___________________Email address: ____________________________

Cell Phone: ______________________ Home Phone: ______________________

NOK#2

First: ______________________Last: ______________________Middle: ______________

Relation to you: ___________________Email address: ____________________________

Cell Phone: ______________________ Home Phone: ______________________

NOK#3

First: ______________________Last: ______________________Middle: ______________

Relation to you: ___________________Email address: ____________________________

Cell Phone: ______________________ Home Phone: ______________________

**MAILING ADDRESS**

**18. Do you have a P.O. Box or a mailing address that is different than your home address?**

0 ❑ No

1 ❑ Yes…what is your P.O. Box or mailing address?

Address: _________________________________________________________________

City: ___________________________State: ________________Zip: ________________

**STRESS EVALUATION**

**Questions 19-28 Perceived Stress Scale**

Cohen S, Kamarck T, Mermelstein R: **A global measure of perceived stress**. *J Health Soc Behav* 1983, **24**(4):385-396.

**Questions 29-48 CES-D Scale**

Radloff LS: **The CES-D scale: A self report depression scale for research in the general population.** *Applied Psychological Measurements* 1977, **1**:385-401.

**Questions 49-57 The Prenatal Distress Questionnaire**

Yali AM, Lobel M: **Coping and distress in pregnancy: an investigation of medically high risk women**. *J Psychosom Obstet Gynaecol* 1999, **20**(1):39-52.

**DEMOGRAPHIC DATA**

**58**. Are you of Hispanic or Latino ethnicity?

0 ❑ No

1 ❑ Yes

**59.** Is the baby’s biological father of Hispanic or Latino ethnicity?

0 ❑ No

1 ❑ Yes

9 ❑ Don’t Know

**60**. What is your race? (**MARK ALL THAT APPLY**):

1 ❑ White

2 ❑ Asian

3 ❑ Black or African-American

4 ❑ Native Hawaiian or Pacific Islander

5 ❑ American Indian/Alaska Native

6 ❑ Other: Explain: _________________

**61.** What is your baby’s biological father’s race? (**MARK ALL THAT APPLY**)

1 ❑ White

2 ❑ Asian

3 ❑ Black or African-American

4 ❑ Native Hawaiian or Pacific Islander

5 ❑ American Indian/Alaska Native

6 ❑ Other: Explain:_________________

9 ❑ Don’t Know

**62.** Were you born in the USA?

1 ❑ Yes

0 ❑ No…

1. **Where were you born? ________________________________**
2. **How long have you lived in the USA? _____________**

**63.** Was your baby’s biological father born in the USA?

1 ❑ Yes

0 ❑ No…

1. **Where was he born? ____________________________________________**
2. **How long has he lived in the USA? _____________** ❑**Never lived in USA**

9 ❑ Don’t Know

**64.** What is your marital status? **[MARK ONE]**

1 ❑ Married

2 ❑ Living together

3 ❑ Never married, single

4 ❑ Divorced or separated

5 ❑ Widowed

6 ❑ Decline to answer

**65.** What was the last grade in school **you** completed? **[MARK ONE]**

1 ❑ Less than 12th grade (did not finish high school)

2 ❑ Completed grade 12 (high school)

3 ❑ Some college or technical school

4 ❑ Completed 4 years of college

5 ❑ Some graduate training after college

**66.** What was the last grade in school **the baby’s biological father** completed? **[MARK ONE]**

1 ❑ Less than 12th grade (did not finish high school)

2 ❑ Completed grade 12 (high school)

3 ❑ Some college or technical school

4 ❑ Completed 4 years of college

5 ❑ Some graduate training after college

9 ❑ Don’t know

**Occupational history**

**67**. What is your current employment status? (**MARK ALL THAT APPLY**)

1 ❑ Homemaker

2 ❑ Student

3 ❑ Employed

4 ❑ Temporary medical leave

5 ❑ Unemployed

6 ❑ Other: Explain: ____________________

**68.** Have you been working during this pregnancy?

0 ❑ No (**SKIP TO #72**)

1 ❑ Yes

**69.** How many hours do you work per week?

1 ❑ Less than 10 hours/week

2 ❑ 10-20 hours/week

3 ❑ 21-30 hours/week

4 ❑ 31-40 hours/week

5 ❑ More than 40 hours/week

**70**. During a regular work week, how many days do you commute to and from your work location?

0 ❑ 0 days

1 ❑ 1 day

2 ❑ 2 days

3 ❑ 3 days

4 ❑ 4 days

5 ❑ 5 days

6 ❑ 6 days

7 ❑ 7 days

**71.** Thinking about your typical commute **TO** your job, which forms of transportation do you use and for how long? (Mark all that apply.)

|  | **1-10**  **Minutes** | **11-20**  **Minutes** | **21-30**  **Minutes** | **31-59**  **Minutes** | **60-90**  **Minutes** | **91-120**  **Minutes** | **2 Hours or More** | **N/A** |
| --- | --- | --- | --- | --- | --- | --- | --- | --- |
| Car |  |  |  |  |  |  |  |  |
| Bus or  Tram |  |  |  |  |  |  |  |  |
| Train or Metro |  |  |  |  |  |  |  |  |
| By motorcycle |  |  |  |  |  |  |  |  |
| By bike |  |  |  |  |  |  |  |  |
| On foot |  |  |  |  |  |  |  |  |

**PRE-PREGNANCY PHYSICAL ACTIVITY AND WEIGHT**

**72.** What was your weight in pounds before this pregnancy? **__________________**

**73.** The next question will ask about how much physical activity per week you typically did before you were pregnant. (*Physical Activity is any activity that increases your heart rate and makes you get out of breath some of the time.* *Physical activity can be done through sports, exercise, classes, housework, yardwork, travelling to get somewhere. Some examples of physical activity are running, brisk walking, biking, dancing, swimming, yoga, aerobics classes, and mowing the lawn.)*

Please think back to a typical week **during the 6 months before you became pregnant**. On how many days per week were you physically active for a total of at least 30 minutes, not necessarily consecutive, per day? (Please check one)

1❑ 0 days per week 5❑ 4 days per week

2❑ 1 day per week 6❑ 5 days per week

3❑ 2 days per week 7❑ 6 days per week

4❑ 3 days per week 8❑ 7 days per week

**PREGNANCY INFORMATION**

**74.** **Prior to finding out you were pregnant**, did you take any multivitamins or prenatal vitamins?

0 ❑ No

1 ❑ Yes:

**A.** How long prior to finding out you were pregnant did you begin taking multivitamins or prenatal vitamins?

1 ❑ Less than 3 months before

2 ❑ 3 to 6 months before

3 ❑ 6 to 12 months before

4 ❑ More than 12 months before

**B.** How many vitamin tablets were you taking?

1 ❑ 1 to 3 per week

2 ❑ 4 to 6 per week

3 ❑ 1 per day

4 ❑ More than 1 per day

9❑ Don’t remember

**C.** Did your vitamin tablet contain folic acid?

0 ❑ No

1 ❑ Yes

9 ❑ Don’t know

**75.** **Prior to finding out you were pregnant**, were you taking an individual folic acid tablet?

0 ❑ No

1 ❑ Yes:

**A.** How long prior to finding out you were pregnant did you begin taking an individual folic acid tablet?

1 ❑ Less than 3 months before

2 ❑ 3 to 6 months before

3 ❑ 6 to 12 months before

4 ❑ More than 12 months before

**B.** How many tablets were you taking?

1 ❑ 1 to 3 per week

2 ❑ 4 to 6 per week

3 ❑ 1 per day

4 ❑ More than 1 per day

9❑ Don’t remember

9 ❑ Don’t know

**76.** **Prior to finding out you were pregnant**, were you taking iron?

0 ❑ No

1 ❑ Yes, an individual iron tablet:

**A.** How long prior to finding out you were pregnant did you begin taking iron?

1 ❑ Less than 3 months before

2 ❑ 3 to 6 months before

3 ❑ 6 to 12 months before

4 ❑ More than 12 months before

**B.** How many iron tablets were you taking?

1 ❑ 1 to 3 per week

2 ❑ 4 to 6 per week

3 ❑ 1 per day

4 ❑ More than 1 per day

9❑ Don’t remember

9 ❑ Don’t know

**77.** **During this pregnancy**, have you taken any multivitamins or prenatal vitamins?

0 ❑ No

1 ❑ Yes:

**A.** How many vitamin tablets do you take?

1 ❑ 1 to 3 per week

2 ❑ 4 to 6 per week

3 ❑ 1 per day

4 ❑ More than 1 per day

9 ❑ Don’t remember

**B.** Does your vitamin tablet contain folic acid?

0 ❑ No

1 ❑ Yes

9 ❑ Don’t know

**78. During this** **pregnancy**, have you taken an individual folic acid tablet?

0 ❑ No

1 ❑ Yes

**A.** How many folic acid tablets do you take?

1 ❑ 1 to 3 per week

2 ❑ 4 to 6 per week

3 ❑ 1 per day

4 ❑ More than 1 per day

5 ❑ Don’t remember

9❑ Don’t know

**79**. Were you using birth control when you became pregnant with this pregnancy?

0 ❑ No

1. Were you trying to become pregnant?

0 ❑ No

1 ❑ Yes

1 ❑ Yes:

**A.** What type of birth control were you using? (MARK ALL THAT APPLY)

1 ❑ Birth control pills

2 ❑ Depo “the shot”

3 ❑ Contraceptive implant

4 ❑ IUD (copper)

5 ❑ IUD (hormonal)

6 ❑ Patch

7 ❑ Condoms (male or female)

8 ❑ Diaphragm

9 ❑ Contraceptive sponge

10 ❑ Vaginal ring (e.g. NuvaRing)

11 ❑ Cervical cap

12 ❑ Morning-After pill

13 ❑ Spermicide

14 ❑ Tubal sterilization or Vasectomy

15 ❑ Behavioral methods (outercourse, pulling out, rhythm, breast feeding, etc.)

**B.** How long had you been using this type of birth control? (Repeat question for each answer given in 79YesA)

1 ❑ 0-3 months

2 ❑ 3 month – 1 year

3 ❑ More than 1 year

**80.** What is the birth order of this baby?

1 ❑ 1 (first-born)

2 ❑ 2 (second-born)

3 ❑ 3 (third-born)

4 ❑ 4 (fourth-born)

5 ❑ 5 (fifth-born)

6 ❑ 6 or more (sixth-born or later)

**81**. Has a doctor ever said you had asthma?

1 ❑ No (**SKIP TO #86**)

2 ❑ Yes:

**A.**  About how old were you when a doctor first said you had asthma?  Age: _______

**B.** Have you had problems with asthma DURING the time that you have been pregnant (even if you did not know that you were pregnant)?

1 ❑ No

2 ❑ Yes

**82. Since you have been pregnant,** have you required medication for asthma or wheezing?

1 ❑ No

2 ❑ Yes

**83.** **Since you have been pregnant**, how often have you used albuterol (or other short-acting or “rescue” medication) or inhaled bronchodilators for asthma symptoms? **(Check only one)**

|  |  |  |  |  |
| --- | --- | --- | --- | --- |

*(If further probing is needed, examples include albuterol inhaler, Proventil inhaler, Ventolin inhaler, ProAir inhaler, Atrovent inhaler).*

1 ❑ Never

2 ❑ Less than two days a week

3 ❑ Two or more days a week (but not every day)

4 ❑ Once every day

5 ❑ More than once every day

**84. Since you have been pregnant**, how often have you used inhaled corticosteroid medications or tablets (“controller” medications) to manage your asthma symptoms? *(Check only one).* *(If further probing is needed, examples include Advair, Beclovent Inhaler, Flovent Inhaler, Qvar Inhaler, Pulmicort Inhaler, Vanceril Inhaler, Intal Inhaler, Servent Inhaler, Singulair Tablets).*

1 ❑ Never

2 ❑ Less than two days a week

3 ❑ Two or more days a week (but not every day)

4 ❑ Once every day

5 ❑ More than once every day

**85. Since you have been pregnant,** have you taken a course of steroid pills or liquids (i.e. Prednisone, Deltasone, Orasone, Prednicen-M, Liquid Pred) for your asthma symptoms? *A course is defined as one to eight consecutive days of either daily* ***or*** *alternate-day treatment.*

0 ❑ No

1 ❑ Yes

9 ❑ Don’t know

**86.** **Since becoming pregnant**, have you taken any antibiotics?

0 ❑ No

1 ❑ Yes**… For each antibiotic you have taken while pregnant, please give me the name of the antibiotic, how long you took it, and for what illness it was taken.**

|  | **Antibiotic Name** | **Length of Time Taken during pregnancy** | **For what illness?** |
| --- | --- | --- | --- |
| **1** |  |  |  |
| **2** |  |  |  |
| **3** |  |  |  |
| **4** |  |  |  |

**87. Since becoming pregnant,** have you taken any other medications prescribed by a doctor (not including birth control)?

0 ❑ No

1 ❑ Yes… **What other prescription medications did/do you take?**

______________________________________________________________

______________________________________________________________

**88.** **Since becoming pregnant,** have you taken any over-the-counter medications (not including birth control) such as cold medicines, Tylenol, or Advil?

0 ❑ No

1 ❑ Yes… **What other over-the-counter medications did/do you take?**

1 ❑ Cold medicines

2 ❑ Tylenol/Acetaminophen

3 ❑ Advil/Ibuprofen

4 ❑ Other pain reliever: (Specify :________________)

6 ❑ Antacids (Tums, Rolaids, etc.)

5 ❑ Other over-the-counter-medication: (Specify :________________)

**89. Since becoming pregnant,** have you taken any other natural remedies or traditional medicines to help with nausea symptoms (or “morning sickness”) from your pregnancy?

0 ❑ No

1 ❑ Yes … **What other remedies did/do you take?**

______________________________________________________________

______________________________________________________________

**90.** Have you ever had hay fever also known as allergic rhinitis? (An allergic reaction causing nasal congestion, runny nose, sneezing, nose and eye itching, or excessive tear production when you did not have a cold or the “flu”.)

0 ❑ No

1 ❑ Yes

9 ❑ Don’t know

**91.** Do you have allergies?

0 ❑ No

2 ❑ Yes…what are you allergic to?  MARK ALL THAT APPLY.

1 ❑ Dogs

2 ❑ Cats

3 ❑ Other pets

4 ❑ Foods

5 ❑ Plants or pollen

6 ❑ Mold

7 ❑ Other (explain): ________________________________________________

**Questions 92-94** Pregnancy-Unique Quantification of Emesis and Nausea

Koren G, Boskovic R, Hard M, Maltepe C, Navioz Y, Einarson A. Motherisk-PUQE (pregnancy-unique quantification of emesis and nausea) scoring system for nausea and vomiting of pregnancy. Am J Obstet Gynecol. 2002;186: S228–231.

**SMOKING QUESTIONS**

**95.** Excluding e-cigarettes, have you ever smoked cigarettes, cigars or pipes?

0 ❑ No (**SKIP TO #98**)

1 ❑ Yes

**96.** **During this pregnancy,** excluding e-cigarettes, have you smoked cigarettes, cigars or pipes?

0 ❑ No (**SKIP TO #98**)

1 ❑ Yes

**97.** Have you smoked cigarettes, cigars, or pipes in the last 5 days?

0 ❑ No:

**A.** If you are NOT currently smoking, when did you stop smoking? [**MARK ONE**]

1 ❑ less than 2 weeks ago

2 ❑ 2 to 4 weeks ago

3 ❑ More than 4 weeks ago

4 ❑ Don't remember

**B.** If you are NOT currently smoking, how many cigarettes did you usually smoke *per day*?

1 ❑ 1- 5

2 ❑ 6-10

3 ❑ 11-20

4 ❑ More than 20

1 ❑ Yes:

**A.** How many cigarettes did you usually smoke *per day*?

1 ❑ 1- 5

2 ❑ 6-10

3 ❑ 11-20

4 ❑ More than 20

**98. During this pregnancy**, excluding e-cigarettes, has anyone else living in your home smoked cigarettes, cigars or pipes inside the house?

0 ❑ No (**SKIP TO #101)**

1 ❑ Yes

**99.** **During this pregnancy,** who else in your home has smoked cigarettes, cigars or pipes**? (MARK ALL THAT APPLY)**

1 ❑ Baby's Father

2 ❑ Others

**100. During this pregnancy,** not including yourself, how many people living in your home smoke cigarettes, cigars or pipes?

1 ❑ 1

2 ❑ 2

3 ❑ 3

4 ❑ 4 or more

**101.** **During this pregnancy,** *on average*, how many hours per day have you been *exposed* to cigarette, cigar or pipe smoke because of smoking by others?

1 ❑ 0-1 hour

2 ❑ 1-2 hours

3 ❑ 2-3 hours

4 ❑ 3-4 hours

5 ❑ More than 4 hours

**102.** Have you ever smoked electronic cigarettes/e-cigarettes or other electronic nicotine device (e-hookah, e-cigars, etc.)?

0 ❑ No (**SKIP TO #105**)

1 ❑ Yes

**103.** **During this pregnancy**, have you smoked electronic cigarettes/e-cigarettes or other electronic nicotine device (e-hookah, e-cigars, etc.)?

0 ❑ No **(SKIP TO #105)**

1 ❑ Yes

**104.** Have you smoked electronic cigarettes/e-cigarettes or other electronic nicotine device (e-hookah, e-cigars, etc.) in the last 5 days?

0 ❑ No:

**A.** If you are NOT currently smoking, when did you stop smoking? [**MARK ONE**]

1 ❑ less than 2 weeks ago

2 ❑ 2 to 4 weeks ago

3 ❑ More than 4 weeks ago

4 ❑ Don't remember

1. If you are NOT currently smoking, how often did you smoke electronic cigarettes/e-cigarettes or other electronic nicotine devices (e-hookah, e-cigars, etc.)?

1❑ Every day

2❑ Every few days

3❑ Once a week

4❑ About once a month

5❑ Every few months

1 ❑ Yes:

**A.** How often do you smoke electronic cigarettes/e-cigarettes or other electronic nicotine devices (e-hookah, e-cigars, etc.)?

1❑ Every day

2❑ Every few days

3❑ Once a week

4❑ About once a month

5❑ Every few months

**HOME CHARACTERISTICS**

**105**. **Which best describes the home in which you currently live most of the time**? *Mark one.*

1 ❑ A house (not connected to other homes)

2 ❑ A building with 2-4 attached apartments, town houses, condos, a duplex or a triplex

3 ❑ A building with 5-10 attached apartments, town houses, condos, etc.

4 ❑ A building with more than 10 attached apartments, town houses, condos, etc.

5 ❑ Mobile home or trailer

6 ❑ Other, please be specific: ___________________________________________________

**106**. **About when was this structure originally built**? (When it was first constructed, not when it may have been remodeled, added to, or converted.) *Mark one.*

1❑ 2000s or later

2❑ 1980s-1990s

3❑ 1960s-1970s

4❑ 1940s-1950s

5❑ Before 1940

**107.** **During this pregnancy,** which of the following pets have you kept inside your home?

**(MARK ALL THAT APPLY)**

1 ❑ No Pets

2 ❑ Dog(s)

3 ❑ Cat(s)

4 ❑ Other pets (Explain: ____________)

**108.** **During this pregnancy,** have you had any of the following pests in your home?  **(MARK ALL THAT APPLY)**

1 ❑ Rats

2 ❑ Mice

3 ❑ Cockroaches

4 ❑ Other pests (Specify: ________________)

5 ❑ Don’t know

6 ❑ No pests

**109**. Is there a cooking stove, range, or oven in your home that uses GAS**?**

0 ❑ No

1 ❑ Yes:

**A.** How often is the gas stove, range or oven used while you are at home? *Mark one.*

1 ❑ Never **(SKIP to 111)**

2 ❑ Less than once a week

3 ❑ 1-3 times per week

4 ❑ 4-7 times per week

5 ❑ 8-14 times per week

6 ❑ More than 14 times per week

**B.** About how long is the gas stove, range or oven used on an average day while you are at home?

1 ❑ Less than 15 minutes

2 ❑ 15 minutes to less than 30 minutes

3 ❑ 30 minutes to less than 1 hour

4 ❑ 1 hour or more

**110.** **During this pregnancy,** on average, how many times a week do you cook (using the stove/range/oven, not microwave)?

1 ❑ Never

2 ❑ 1 – 3 times a week

3 ❑ 4 – 5 times a week

4 ❑ Every day of the week

**111.** Does your home have heating?

0 ❑ No **(SKIP to 113)**

1 ❑ Yes:

**A.** What is the main fuel used to heat it? *Mark one.*

1 ❑ Gas (you may be able to see a blue flame or pilot light in the unit)

2 ❑ Electricity (you may be able to see a red-hot glowing wire in the unit)

3 ❑ Bottles, tank or L.P./liquefied petroleum gas (a tank outside that a truck may fill with gas)

4 ❑ Firewood

5 ❑ Other, please be specific: ________________________

9 ❑ Don’t know how it is heated

**112.** What is the one main heating system in your home? *Mark one.*

1 ❑ Forced air

2 ❑ Built-in electric unit

3 ❑ Wall heater

4 ❑ Floor heater

5 ❑ Portable space heater… **Which type?**

1 ❑ Gas

2 ❑ Electric

3 ❑ Don’t Know

6 ❑ Other, please be specific: ______________________________

9 ❑ Don’t know how it is heated

**113.** Do you use air conditioning in your home?

0 ❑ No **(SKIP to 116)**

1 ❑ Yes:

**A.** What is the main kind of air conditioning that is used? *Mark one.*

1 ❑ Wall or window unit (box that sticks out of window or wall)

**a.** How many wall/window units do you have in your home?

1 ❑ One

2 ❑ Two

3 ❑ Three

4 ❑ Four or more

5 ❑ Don’t know

2 ❑ Central (vents in the room)

3 ❑ Swamp/desert/evaporative cooler

9 ❑ Don’t know what kind it is

**114**. **During the last month,** about how often did you use air conditioning when you were at home?

1 ❑ Never

2 ❑ Less than 5 days

3 ❑ 5-15 days

4 ❑ 16-30 days

9 ❑ Don’t know

**115.** On any given day, how much of the time did you use the air conditioner at home?

1 ❑ None of the time

2 ❑ A couple of hours a day

3 ❑ Half of the time

4 ❑ Most of the time

5 ❑ All of the time

9 ❑ Don’t know

**116.** During the last month, did you use a window fan or other fan that you placed in the window or an attic fan to cool your home?

0 ❑ No

1 ❑ Yes

**117.** Has there been water damage OR flooding in your home since becoming pregnant?

0 ❑ No

1 ❑ Yes:

**A.** Did it flood carpeted areas?

0 ❑ No

1 ❑ Yes

9 ❑ Don’t know

**118.** Has there ever been mold or mildew on the walls, ceilings, or floors inside your home since becoming pregnant?

0 ❑ No

1 ❑ Yes:

**A.** Which rooms were affected? *Mark all that apply.*

1 ❑ The room where you sleep

2 ❑ Bathroom(s)

3 ❑ Basement

4 ❑ Other

9 ❑ Don’t know

**119.** Is a humidifier or vaporizer ever used in your home? (Include humidifier built into heating system.)

0 ❑ No

1 ❑ Yes:

**A.** What type is it? *Mark all that apply.*

1 ❑Built into heating system

2 ❑Free standing or portable unit

**B.** Have you used this unit for treating a respiratory illness?

0 ❑No

1 ❑Yes

**C.**  Does the humidifier or vaporizer heat the air?

0 ❑No

1 ❑Yes

9 ❑Don’t know

9 ❑ Don’t know

**120**. Is there carpeting in your home?

0 ❑ No

1 ❑ Yes:

**A.** In what rooms? *Mark all that apply.*

1 ❑ Whole house (excluding kitchen and bath)

2 ❑ Room where you sleep

3 ❑ Other bedroom(s)

4 ❑ Other room(s)

**121.** Thinking back to a typical *weekday* in this past **week**, approximately how many hours (out of 24 hours in total) did you spend…

**A.** Outdoors: ________________

**B.** Indoors at home (Include nighttime/sleeping):____________________

**122.** On average, how much of the time were the windows open in your home this past **week**?

1 ❑ None of the time

2 ❑ A couple of hours a day

3 ❑ Half of the time

4 ❑ Most of the time

5 ❑ All of the time

9 ❑ Don’t know

**SLEEP QUESTIONS**

**Next, we are going to ask you about your sleeping patterns and habits during the past month (30 days). Think about over the past month (30 days).**

**123. In the past month,** how many hours of sleep did you usually get on a typical weeknight (Sunday - Thursday)?

1 ❑ Less than 4 hours per night _5_ ❑ 8 hours per night

2 ❑ 5 hours per night _6_ ❑ 9 hours per night

3 ❑ 6 hours per night _7_ ❑ More than 10 hours per night

4 ❑ 7 hours per night

**124.** **In the past month,** how many hours of sleep did you usually get on a typical weekend night (Friday or Saturday)?

1 ❑ Less than 4 hours per night _5_ ❑ 8 hours per night

2 ❑ 5 hours per night _6_ ❑ 9 hours per night

3 ❑ 6 hours per night _7_ ❑ More than 10 hours per night

4 ❑ 7 hours per night

**Questions 125-128 Jenkins Sleep Questionnaire**

Jenkins CD, Stanton B-A, Niemcrym SJ, Rose RM. A scale for the estimation of sleep problems in clinical research. J Clin Epidemiol 1988;41:313-21.

**129.** **During the LAST YEAR, how often, on average, have you snored or been told you snore DURING SLEEP? (MARK ONE)**

1 ❑ Never

2 ❑ Rarely (Less than once a week)

3 ❑ Sometimes (1 to 2 times per week)

4 ❑ Frequently (3 to 4 times per week)

5 ❑ Always/Almost always (5 to 7 times per week)

9 ❑ Not sure

**HEALTH CARE ACCESS**

**130.** In the past 12 months, how many times did you visit a doctor?

______________ number of times

**131.** During the past year, have you ever received help in applying for health insurance for yourself?

0 ❑ No

1 ❑ Yes:

1. Are you currently receiving help in applying for health insurance for yourself?

0 ❑ No

1 ❑ Yes

**INCOME**

**132.** In which of the following categories did your TOTAL HOUSEHOLD FAMILY INCOME fall in last year? Include all incomes, before taxes and deductions, of all members of the family.

1 ❑ Less than $15,000

2 ❑ $15,000 to $29,999

3 ❑ $30,000 to $49,999

4 ❑ $50,000 to $99,999

5 ❑ $100,000 or more

9 ❑ Don’t know
