# Supplementary material for: Study Design, Protocol and Profile of the Maternal And Developmental Risks from Environmental and Social Stressors (MADRES) Pregnancy Cohort: a Prospective Cohort Study in Predominantly Low-Income Hispanic Women in Urban Los Angeles
Source: BMC Pregnancy Childbirth. 2019 May 30;19:189. doi: 10.1186/s12884-019-2330-7 (PMC6543670; doi:10.1186/s12884-019-2330-7)
Supplement: Supplementary file 7 — MADRES_PPAQ. Pregnancy physical activity questionnaire conducted at the first study visit and the third trimester study visit. (DOCX 16 kb) [file 12884_2019_2330_MOESM7_ESM.docx]

**MADRES: Pregnancy Physical Activity Questionnaire (PPAQ)**

**Questions 1-33**

Chasan-Taber L, Schmidt MD, Roberts DE, Hosmer D, Markenson G, Freedson PS: **Development and validation of a Pregnancy Physical Activity Questionnaire**. *Med Sci Sports Exerc* 2004, **36**(10):1750-1760.
