# Supplementary material for: Study Design, Protocol and Profile of the Maternal And Developmental Risks from Environmental and Social Stressors (MADRES) Pregnancy Cohort: a Prospective Cohort Study in Predominantly Low-Income Hispanic Women in Urban Los Angeles
Source: BMC Pregnancy Childbirth. 2019 May 30;19:189. doi: 10.1186/s12884-019-2330-7 (PMC6543670; doi:10.1186/s12884-019-2330-7)
Supplement: Supplementary file 12 — MADRES Third Trimester Questionnaire_Spanish. Spanish questionnaire administered during the third trimester visit to participants recruited before 20 weeks of pregnancy. (DOCX 159 kb) [file 12884_2019_2330_MOESM12_ESM.docx]

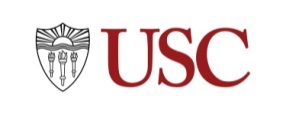
 **MADRES Study: Third Trimester Questionnaire**

**Today’s Date:** _____________________ **Interviewer Name:** ____________________

**Instrucciones:** Gracias por aceptar participar en este estudio. Durante esta entrevista le hare preguntas sobre su salud e historial médica. Por favor responda a todas las preguntas de este cuestionario, aún si no está completamente segura de la respuesta. Le aseguramos que sus respuestas serán confidenciales**.** Por favor, con toda confianza, siéntase libre de interrumpirme y hacerme cualquier pregunta que tenga.

**INFORMACION DE CONTACTO**

**1. Nombre:** ________________ _______________ ____________________ ____________________

Nombre 2do Nombre Apellido 1 Apellido 2

**2. Otros nombres que haya usado** (ej. nombre de soltera) ­­­­­­­­­­­­­­­­­­­­­­­­­:­___________________________

**3**. **Fecha de nacimiento:** _**______/_______/_______**

Mes Día Año

**4**. **Fecha estimada de nacimiento:** **_______/_______/_______**

Mes Día Año

**5.** **Fecha de su última menstruación:** **_______/_______/_______**

Mes Día Año

**6. ¿Cuál es su número de celular?** ____________________________

□₀ No tengo teléfono celular **(Skip to question #8)**

**7.** **¿Es un celular pre pagado o es un número fijo de celular?**

□₀ Pre pagado

□₁ Número fijo

**8.** **¿Cuál es su dirección? (la dirección donde usted pasa la mayoría del tiempo):**

Dirección: ________________________________________________________________________

Ciudad: _____________________Estado: ________________Código Postal: ___________________

**8A.** **If moved…¿Cuando se mudó a su nueva dirección?** _______________________

**9A. Por favor dígame los nombres de otros adultos que viven con usted:**

Adult#1 Nombre: ______________________Apellido: ______________________2do Nombre: ______________

Relación: ___________________ Número de Celular: ______________________

Adult#2 Nombre: ______________________Apellido: ______________________2do Nombre: ______________

Relación: ___________________ Número de Celular: ______________________

Adult#3 Nombre: ______________________Apellido: ______________________2do Nombre: ______________

Relación: ___________________ Número de Celular: ______________________

**10. ¿Cuál es el número de teléfono para el domicilio dado en la Pregunta 8?**______________________

□₀ No tengo teléfono de casa

**11. ¿Vive en más de una casa?**

□₁ Sí... *Complete questions 12A, 12B and 12C* □₀No…  *Go to question #13*

**12A. ¿Cuál es la dirección de su segundo domicilio?**

Dirección: _________________________________________________________________________

Ciudad: ______________________Estado: ________________ Código Postal: __________________

**12A2.** **If moved…¿Cuando se mudó a su nuevo segundo domicilio?** _______________________

**12B. ¿Cuál es el número de teléfono para el domicilio dado en la Pregunta 12A?**_______________________ □₀ No tengo teléfono de casa

**12C. ¿Cuánto tiempo pasa usted en la dirección dada en la pregunta 12A?**

 1%-25% del tiempo

 26%-50% del tiempo

**13. A. ¿Cuál es su correo electrónico?** _________________________ 0 ❑No tengo correo electrónico

**B. ¿Cuál es su nombre de usuario en Facebook?** ___________________________0 ❑No tengo Facebook

**C. ¿Cuál es su nombre de usuario en Twitter?** @___________________________0 ❑No tengo Twitter

**D. ¿Cuál es su nombre de contacto para Instagram?** ____________________0 ❑No tengo Instagram

**14. A. ¿Cómo prefiere ser contactada?**

 Teléfono

 Correo electrónico

 Texto

 Otro: ________________

**B. ¿Cuáles son los mejores días para contactarla?**

 lunes

 martes

 miércoles

 jueves

 viernes

 sábado

 domingo

**C. ¿Cuáles son las mejores horas para contactarla (lunes)?**

 Mañana (8am-12pm)

 Tarde (12pm-5pm)

 Noche (5pm-8pm)

 Otro: ______________

**D. ¿Cuáles son las mejores horas para contactarla (martes)?**

 Mañana (8am-12pm)

 Tarde (12pm-5pm)

 Noche (5pm-8pm)

 Otro: ______________

**E. ¿Cuáles son las mejores horas para contactarla (miércoles)?**

 Mañana (8am-12pm)

 Tarde (12pm-5pm)

 Noche (5pm-8pm)

 Otro: ______________

**F. ¿Cuáles son las mejores horas para contactarla (jueves)?**

 Mañana (8am-12pm)

 Tarde (12pm-5pm)

 Noche (5pm-8pm)

 Otro: ______________

**G. ¿Cuáles son las mejores horas para contactarla (viernes)?**

 Mañana (8am-12pm)

 Tarde (12pm-5pm)

 Noche (5pm-8pm)

 Otro: ______________

**H. ¿Cuáles son las mejores horas para contactarla (sábado)?**

 Mañana (8am-12pm)

 Tarde (12pm-5pm)

 Noche (5pm-8pm)

 Otro: ______________

**I . ¿Cuáles son las mejores horas para contactarla (domingo)?**

 Mañana (8am-12pm)

 Tarde (12pm-5pm)

 Noche (5pm-8pm)

 Otro: ______________

**15.** **¿Cómo se llama el papa del bebe?** □ No sé

_________________ _______________ ____________________ ____________________

Nombre 2do Nombre Apellido 1 Apellido 2

**16A.** **¿Tiene usted esposo o pareja?** 0 ❑ No…*Go to Question* 17 1 ❑ Sí

**16 B. ¿Cómo se llama su esposo/pareja?**

_________________ _______________ ____________________ ____________________

Nombre 2do Nombre Apellido 1 Apellido 2

**17.** **Para poder localizarla en caso de que se mude o cambie su número de teléfono, ¿nos puede dar la información de su madre y tres amigos o familiares que no vivan con usted que nos podrían dar su información nueva?**

INFORMACION DE SU MADRE

Nombre: ______________________Apellido: ______________________2do Nombre: ______________

Dirección: _________________________________________________________________________

Ciudad: ______________________Estado: ________________ Código Postal: __________________

Número de Celular: ______________________ Número de teléfono de Casa: ______________________

NOK#1

Nombre: ______________________Apellido: ______________________2do Nombre: ______________

Relación: ___________________Correo Electrónico: ____________________________

Número de Celular: ______________________Número de teléfono de Casa: ______________________

NOK#2

Nombre: ______________________Apellido: ______________________2do Nombre: ______________

Relación: ___________________Correo Electrónico: ____________________________

Número de Celular: ______________________Número de teléfono de Casa: ______________________

NOK#3

Nombre: ______________________Apellido: ______________________2do Nombre: ______________

Relación: ___________________Correo Electrónico: ____________________________

Número de Celular: ______________________Número de teléfono de Casa: ______________________

**DIRECCIÓN DE ENVIO**

**18. ¿Tienes una dirección postal o postal diferente a la dirección de su domicilio?**

0 ❑ No

1 ❑ Sí… ¿cuál es su dirección postal?

Dirección: _____________________________________________________________

Ciudad: ______________________Estado: ___________ Código Postal: _______________

**EVALUACION DEL ESTRES**

**Questions 19-28 Perceived Stress Scale**

Cohen S, Kamarck T, Mermelstein R: **A global measure of perceived stress**. *J Health Soc Behav* 1983, **24**(4):385-396.

**Questions 29-48 CES-D Scale**

Radloff LS: **The CES-D scale: A self report depression scale for research in the general population.** *Applied Psychological Measurements* 1977, **1**:385-401.

**Questions 49-65 The Prenatal Distress Questionnaire**

Yali AM, Lobel M: **Coping and distress in pregnancy: an investigation of medically high risk women**. *J Psychosom Obstet Gynaecol* 1999, **20**(1):39-52.

**EVENTOS ESTRESANTES DURANTE EL EMBARAZO**

**Questions 66 (66a-66m)**

Life course indicator: stressors during pregnancy (LC-56) Association of Maternal and Child Health Programs, <http://www.amchp.org/programsandtopics/data-assessment/LifeCourseIndicatorDocuments/LC-56_Stressors%20During%20Pregnancy_Final-12-16-2013.pdf>

**HISTORIAL OCUPACIONAL**

**67**. ¿Cuál es su actual estado de empleo? MARK ALL THAT APPLY]

1 ❑ Ama de casa

2 ❑ Estudiante

3 ❑ Empleada

4 ❑ En permiso médico temporal

5 ❑ Desempleada

6 ❑ Otro: Explique: ____________________

**68.** ¿Ha estado trabajando **desde la última vez que la vimos/hablamos durante su primer/segundo trimestre, el/en (fecha determinada)**?

0 ❑ No (**SKIP TO #72**)

1 ❑ Sí

**69.** ¿Cuántas horas a la semana trabaja?

1 ❑ Menos de 10 horas/semana

2 ❑ 10-20 horas/semana

3 ❑ 21-30 horas/semana

4 ❑ 31-40 horas/semana

5 ❑ Más de 40 horas/semana

**70**. Durante una semana de trabajo regular, ¿cuantos días a la semana le toca viajar de ida y de regreso del trabajo?

0 ❑ 0 día

1 ❑ 1 día

2 ❑ 2 días

3 ❑ 3 días

4 ❑ 4 días

5 ❑ 5 días

6 ❑ 6 días

7 ❑ 7 días

**71.** Piensa acerca de tu viaje típico de ida al trabajo, ¿qué formas de transporte utilizas y por cuánto tiempo? (Mark all that apply.)

|  | **1-10**  **Minutos** | **11-20**  **Minutos** | **21-30**  **Minutos** | **31-59**  **Minutos** | **60-90**  **Minutos** | **91-120**  **Minutos** | **2 Horas o mas** | **N/A** |
| --- | --- | --- | --- | --- | --- | --- | --- | --- |
| Coche/carro |  |  |  |  |  |  |  |  |
| Bus o  Tranvía |  |  |  |  |  |  |  |  |
| Tren o Metro |  |  |  |  |  |  |  |  |
| Motocicleta |  |  |  |  |  |  |  |  |
| Bicicleta |  |  |  |  |  |  |  |  |
| A pie |  |  |  |  |  |  |  |  |

**INFORMACION SOBRE EL EMBARAZO**

**72. Desde la última vez que la vimos/hablamos durante su primer/segundo trimestre**, **el/en (dar fecha),** ¿ha tomado vitaminas, multivitaminas, o vitaminas prenatales?

0 ❑ No

1 ❑ Sí:

**A.** ¿Cuantas tabletas de vitaminas está tomando?

1 ❑ 1 a 3 por semana

2 ❑ 4 a 6 por semana

3 ❑ 1 por día

4 ❑ Más de 1 por día

9 ❑ No me acuerdo

**B.** ¿Su tableta de vitamina contiene ácido fólico?

0 ❑ No

1 ❑ Sí

9 ❑ No sé

**73. Desde la última vez que la vimos/hablamos durante su primer/segundo trimestre**, **el/en (dar fecha),** ¿ha tomado usted una tableta individual de ácido fólico?

0 ❑ No

1 ❑ Sí:

**A.** ¿Cuantas tabletas de ácido fólico está tomando?

1 ❑ 1 a 3 por semana

2 ❑ 4 a 6 por semana

3 ❑ 1 por día

4 ❑ Más de 1 por día

9 ❑ No me acuerdo

9❑ No sé

**74.** ¿Ha comido alguno de los siguientes tipos de comida de mar **desde la última vez que la vimos/hablamos durante su primer/segundo trimestre**, **el/en (dar fecha)**?

|  | **¿Ha comido alguno de los siguientes tipos de comida de mar durante este embarazo?** | **Y si sí, ¿con qué frecuencia?** | **Y si sí, ¿comió esto en los últimos tres días?** |
| --- | --- | --- | --- |
| Palitos de pescado (cualquiera comercialmente preparado congelado, de pescado blanco sin espinas, desmenuzado y frito en aceite) | 0 ❑ No  1 ❑ Sí  9 ❑ No sé | 1 ❑ Diariamente  2 ❑ Semanal  3 ❑ Mensual  4 ❑ Raramente  9 ❑ No sé | 0 ❑ No  1 ❑ Sí |
| Pescado grasoso (más de un 2% de grasa: bacalao, plata dorada, gemfish, calderón, salmonete, reloj anaranjado, sardinas, gallineta, perca plateada, salmón atlántico, atún del sur, azul granadero, sastre, caballa azul, tarwhine, trucha arcoiris) | 0 ❑ No  1 ❑ Sí  9 ❑ No sé | 1 ❑ Diariamente  2 ❑ Semanal  3 ❑ Mensual  4 ❑ Raramente  9 ❑ No sé | 0 ❑ No  1 ❑ Sí |
| Otros pescados frescos aun no mencionado (pescado no grasoso: lenguado, tiburón) | 0 ❑ No  1 ❑ Sí  9 ❑ No sé | 1 ❑ Diariamente  2 ❑ Semanal  3 ❑ Mensual  4 ❑ Raramente  9 ❑ No sé | 0 ❑ No  1 ❑ Sí |
| Atún enlatada | 0 ❑ No  1 ❑ Sí  9 ❑ No sé | 1 ❑ Diariamente  2 ❑ Semanal  3 ❑ Mensual  4 ❑ Raramente  9 ❑ No sé | 0 ❑ No  1 ❑ Sí |
| Mariscos fritos (camarón, langosta, cangrejos, almejas, mejillones/choros, vieiras/conchas de abanico) | 0 ❑ No  1 ❑ Sí  9 ❑ No sé | 1 ❑ Diariamente  2 ❑ Semanal  3 ❑ Mensual  4 ❑ Raramente  9 ❑ No sé | 0 ❑ No  1 ❑ Sí |
| Mariscos (camarón, langosta, cangrejos, almejas, mejillones/choros, vieiras/conchas de abanico) | 0 ❑ No  1 ❑ Sí  9 ❑ No sé | 1 ❑ Diariamente  2 ❑ Semanal  3 ❑ Mensual  4 ❑ Raramente  9 ❑ No sé | 0 ❑ No  1 ❑ Sí |

**75.** ¿Bebió café o té cafeinado **desde la última vez que la vimos/hablamos durante su primer/segundo trimestre**, **el/en (dar fecha)**?

0 ❑ No

1 ❑ Sí:

**A.** ¿Con qué frecuencia bebió café o té cafeinado **desde la última vez que la vimos/hablamos durante su primer/segundo trimestre**, **el/en (dar fecha)?**

1 ❑ 1 a 3 bebidas por semana

2 ❑ 4 a 6 bebidas por semana

3 ❑ 1-2 bebidas por día

4 ❑ 3-4 bebidas por día

1. ❑ Más de 4 bebidas por día

9❑ No estoy segura

**76.** ¿Bebió alguna de las siguientes bebidas **desde la última vez que la vimos/hablamos durante su primer/segundo trimestre**, **el/en (dar fecha)**?

| **Bebidas** | **¿Bebió esto durante este embarazo?** | **Y si sí, ¿con qué frecuencia?** |
| --- | --- | --- |
| Aguas frescas (p.ej. Horchata, Tamarindo, Agua de sandía) | 0 ❑ No  1 ❑ Sí | 1 ❑ 1 a 3 bebidas por semana  2 ❑ 4 a 6 bebidas por semana  3 ❑ 1 a 2 bebidas por día  4 ❑ 3 a 4 bebidas por día  5 ❑ Más de 4 bebidas por día  9 ❑ No estoy segura |
| Gaseosas con cafeína (p.ej. Coca-Cola, Pepsi, Mountain Dew) | 0 ❑ No  1 ❑ Sí | 1 ❑ 1 a 3 bebidas por semana  2 ❑ 4 a 6 bebidas por semana  3 ❑ 1 a 2 bebidas por día  4 ❑ 3 a 4 bebidas por día  5 ❑ Más de 4 bebidas por día  9 ❑ No estoy segura |
| Gaseosas Descafeinada (p.ej. Sprite, 7-UP) | 0 ❑ No  1 ❑ Sí | 1 ❑ 1 a 3 bebidas por semana  2 ❑ 4 a 6 bebidas por semana  3 ❑ 1 a 2 bebidas por día  4 ❑ 3 a 4 bebidas por día  5 ❑ Más de 4 bebidas por día  9 ❑ No estoy segura |
| Champurrado | 0 ❑ No  1 ❑ Sí | 1 ❑ 1 a 3 bebidas por semana  2 ❑ 4 a 6 bebidas por semana  3 ❑ 1 a 2 bebidas por día  4 ❑ 3 a 4 bebidas por día  5 ❑ Más de 4 bebidas por día  9 ❑ No estoy segura |
| Chocolate Caliente / Chocolate Abuelita | 0 ❑ No  1 ❑ Sí | 1 ❑ 1 a 3 bebidas por semana  2 ❑ 4 a 6 bebidas por semana  3 ❑ 1 a 2 bebidas por día  4 ❑ 3 a 4 bebidas por día  5 ❑ Más de 4 bebidas por día  9 ❑ No estoy segura |
| Bebidas energéticas (p.ej. Red Bull, Rockstar, Monster) | 0 ❑ No  1 ❑ Sí | 1 ❑ 1 a 3 bebidas por semana  2 ❑ 4 a 6 bebidas por semana  3 ❑ 1-2 bebidas por día  4 ❑ 3-4 bebidas por día  5 ❑ Más de 4 bebidas por día  9 ❑ No estoy segura |
| Café con sabor | 0 ❑ No  1 ❑ Sí | 1 ❑ 1 a 3 bebidas por semana  2 ❑ 4 a 6 bebidas por semana  3 ❑ 1 a 2 bebidas por día  4 ❑ 3 a 4 bebidas por día  5 ❑ Más de 4 bebidas por día  9 ❑ No estoy segura |
| Leche con sabor (p.ej. leche de chocolate, leche de fresa) | 0 ❑ No  1 ❑ Sí | 1 ❑ 1 a 3 bebidas por semana  2 ❑ 4 a 6 bebidas por semana  3 ❑ 1 a 2 bebidas por día  4 ❑ 3 a 4 bebidas por día  5 ❑ Más de 4 bebidas por día  9 ❑ No estoy segura |
| Bebidas con sabor a frutas (p.ej. Snapple) | 0 ❑ No  1 ❑ Sí | 1 ❑ 1 a 3 bebidas por semana  2 ❑ 4 a 6 bebidas por semana  3 ❑ 1 a 2 bebidas por día  4 ❑ 3 a 4 bebidas por día  5 ❑ Más de 4 bebidas por día  9 ❑ No estoy segura |
| Jarritos | 0 ❑ No  1 ❑ Sí | 1 ❑ 1 a 3 bebidas por semana  2 ❑ 4 a 6 bebidas por semana  3 ❑ 1 a 2 bebidas por día  4 ❑ 3 a 4 bebidas por día  5 ❑ Más de 4 bebidas por día  9 ❑ No estoy segura |
| Jugo (p.ej. jugo de naranja, jugo de manzana, jugo de arándano rojo) | 0 ❑ No  1 ❑ Sí | 1 ❑ 1 a 3 bebidas por semana  2 ❑ 4 a 6 bebidas por semana  3 ❑ 1 a 2 bebidas por día  4 ❑ 3 a 4 bebidas por día  5 ❑ Más de 4 bebidas por día  9 ❑ No estoy segura |
| Bebidas en polvo (p.ej. Tang, Kool-Aid) | 0 ❑ No  1 ❑ Sí | 1 ❑ 1 a 3 bebidas por semana  2 ❑ 4 a 6 bebidas por semana  3 ❑ 1 a 2 bebidas por día  4 ❑ 3 a 4 bebidas por día  5 ❑ Más de 4 bebidas por día  9 ❑ No estoy segura |
| Bebidas deportivas (p.ej. Gatorade) | 0 ❑ No  1 ❑ Sí | 1 ❑ 1 a 3 bebidas por semana  2 ❑ 4 a 6 bebidas por semana  3 ❑ 1 a 2 bebidas por día  4 ❑ 3 a 4 bebidas por día  5 ❑ Más de 4 bebidas por día  9 ❑ No estoy segura |
| Té dulce (Caliente o Frío) | 0 ❑ No  1 ❑ Sí | 1 ❑ 1 a 3 bebidas por semana  2 ❑ 4 a 6 bebidas por semana  3 ❑ 1 a 2 bebidas por día  4 ❑ 3 a 4 bebidas por día  5 ❑ Más de 4 bebidas por día  9 ❑ No estoy segura |

**77.** ¿Comió **arroz desde la última vez que la vimos/hablamos durante su primer/segundo trimestre**, **el/en (dar fecha)**?

0 ❑ No

1 ❑ Sí:

1. ¿Con que frecuencia comió **arroz desde la última vez que la vimos/hablamos durante su primer/segundo trimestre**, **el/en (dar fecha)**?

1 ❑ 1-6 veces por año 6 ❑ 2 veces por semana

2 ❑ 7-11 veces por año 7 ❑ 3-4 veces por semana

3 ❑ 1 vez por mes 8 ❑ 5-6 veces por semana

4 ❑ 2-3 veces por mes 9 ❑ 1 vez al día

5 ❑ 1 vez por semana A ❑ 2 o más veces al día

1. Por cada vez que comió **arroz desde la última vez que la vimos/hablamos durante su primer/segundo trimestre**, **el/en (dar fecha)**, ¿Qué cantidad comió generalmente?

1 ❑ Menos de ½ taza

2 ❑ ½ a 1½ tazas

3 ❑ Más de 1½ taza

**78.** ¿Comió **arroz en los últimos tres días**?

0 ❑ No

1 ❑ Sí: A. ¿Con que frecuencia comió **arroz en los últimos tres días**?

1 ❑ 1 vez al día

2 ❑ 2 o más veces al día

1. Por cada vez que comió **arroz en los últimos tres días**, ¿qué cantidad comió generalmente?

1 ❑ Menos de ½ taza

2 ❑ ½ a 1½ tazas

3 ❑ Más de 1½ taza

**79**. ¿Le ha dicho su doctor alguna vez que ha tenido asma?

0 ❑ No (**SKIP TO #84**)

1 ❑ Sí:

**A.**  ¿Qué edad tenía la primera vez que el doctor le dijo que tenía asma?  Edad: _______

**B.** ¿Ha tenido usted problemas con asma DURANTE el tiempo que ha estado embarazada (aun no sabiendo que estaba embarazada)?

0 ❑ No

1 ❑ Sí

**80. desde la última vez que la vimos/hablamos durante su primer/segundo trimestre**, **el/en (dar fecha),** ¿ha requerido medicamentos para el asma o silbidos en el pecho?

0 ❑ No

1 ❑ Sí

|  |  |  |  |  |
| --- | --- | --- | --- | --- |

**81.** **Desde la última vez que la vimos/hablamos durante su primer/segundo trimestre**, **el/en (dar fecha),** ¿con qué frecuencia uso albuterol (o cualquier otro medicamento de acción corta o alivio rápido) o uso un broncodilatador inhalado para aliviar síntomas del asma? **(Check only one)**

*(If further probing is needed, examples include: inhaladores, de albuterol, Proventil, Ventolin, ProAir, Atrovent).*

1 ❑ Nunca

2 ❑ Menos de 2 veces a la semana

3 ❑ Dos días o más a la semana (pero no todos los días)

4 ❑ Una vez al día

5 ❑ Más de una vez al día

**82. Desde la última vez que la vimos/hablamos durante su primer/segundo trimestre**, **el/en (dar fecha),** ¿con que frecuencia ha tenido que usar inhaladores o tabletas corticosteroides para controlar su síntomas de asma?

*Check only one).* *(If further probing is needed, examples include Advair, Inhaladores como Beclovent, Flovent, Qvar, Pulmicort, Vanceril, Intal, Servent, tabletas de Singulair).*

1 ❑ Nunca

2 ❑ Menos de 2 veces a la semana

3 ❑ Dos días o más a la semana (pero no todos los días)

4 ❑ Una vez al día

5 ❑ Más de una vez al día

**83. Desde la última vez que la vimos/hablamos durante su primer/segundo trimestre**, **el/en (dar fecha),** ¿ha tenido que tomar un curso de tratamiento de tabletas o líquidos esteroides (e.d. Prednisona, Deltasone, Orasone, Prednicen-M, Liquid Pred) para sus síntomas de asma? *Un curso de tratamiento se considera como uno a ocho días consecutivos o tratamiento de cada otro día..*

0 ❑ No

1 ❑ Sí

9 ❑ No sé

**84.** **desde la última vez que la vimos/hablamos durante su primer/segundo trimestre**, **el/en (dar fecha),** ¿ha tomado antibióticos?

0 ❑ No

1 ❑ Sí**… Para cada antibiótico que ha tomado durante este embarazo, díganos por favor el nombre del antibiótico, por cuanto tiempo lo tomo y que enfermedad estaba tratando.**

|  | **Nombre del Antibiótico** | **Duración del tratamiento durante embarazo** | **La enfermedad siendo tratada** |
| --- | --- | --- | --- |
| **1** |  |  |  |
| **2** |  |  |  |
| **3** |  |  |  |
| **4** |  |  |  |

**85. Desde la última vez que la vimos/hablamos durante su primer/segundo trimestre**, **el/en (dar fecha),** ¿ha tenido que tomar algún otro medicamento prescrita por un doctor (no incluyendo anticonceptivos)?

0 ❑ No

1 ❑ Sí… **¿Que otro medicamente prescrito ha tenido o tiene que tomar?**

______________________________________________________________

______________________________________________________________

**86.** **Desde la última vez que la vimos/hablamos durante su primer/segundo trimestre**, **el/en (dar fecha),** ¿ha tenido que tomar medicamento no prescrito como el Tylenol, Advil o medicamento para la gripe (no incluyendo anticonceptivos)?

0 ❑ No

1 ❑ Sí… ¿**Que medicamento no prescrita ha tenido o tiene que tomar?**

1 ❑ Medicina para el resfrió/la gripe

2 ❑ Tylenol/Acetaminofén

3 ❑ Advil/Ibuprofeno

4 ❑ Otro medicamento para aliviar el dolor: (Especifique:________________)

6 ❑ Antiácidos o medicamento para la acidez (Tums, Rolaids, etc.)

5 ❑ Otro tipo de medicamento no prescrito: (Especifique:________________)

**87. Desde la última vez que la vimos/hablamos durante su primer/segundo trimestre**, **el/en (dar fecha),** ¿ha tomado suplementos naturales, remedios alternativos, o tratamientos tradicionales para ayudarle con los síntomas de nauseas causadas por el embarazo?

0 ❑ No

1 ❑ Sí… **¿Cuáles son los remedios que ha tomado o sigue tomando?**

______________________________________________________________

**88. Durante este embarazo,** ¿le ha dicho un doctor que tiene anemia o hemoglobina baja en la sangre?

0 ❑ No 1 ❑ Sí

**89.** **Durante este embarazo,** ¿le ha dicho un doctor que ha desarrollado diabetes gestacional?

0 ❑ No 1 ❑ Sí

**90. Durante este embarazo,** ¿le ha dicho un doctor que ha desarrollado preeclampsia o presión alta?

0 ❑ No 1 ❑ Sí

**91. Durante este embarazo,** ¿le ha dicho un doctor que ha desarrollado alguna otra condición médica?

0 ❑ No 1 ❑ Sí

**Questions 92-94** Pregnancy-Unique Quantification of Emesis and Nausea

Koren G, Boskovic R, Hard M, Maltepe C, Navioz Y, Einarson A. Motherisk-PUQE (pregnancy-unique quantification of emesis and nausea) scoring system for nausea and vomiting of pregnancy. Am J Obstet Gynecol. 2002;186: S228–231.

**PREGUNTAS SOBRE EL USO DEL CIGARRILLO**

**95.** Sin incluir cigarrillos eléctricos, ¿Ha fumado usted cigarrillos, cigarros o pipas alguna vez en su vida?

0 ❑ No (**SKIP TO #98**)

1 ❑ Sí

**96.** **Desde la última vez que la vimos/hablamos durante su primer/segundo trimestre**, **el/en (dar fecha),** sin incluir cigarrillos eléctricos, ¿ha fumado usted cigarrillos, cigarros o pipas?

0 ❑ No (**SKIP TO # 98**)

1 ❑ Sí

**97.** ¿Ha fumado en los últimos 5 días?

0 ❑ No:

**A.** Si NO fuma actualmente**,** ¿cuándo dejo de fumar? [**MARK ONE**]

1 ❑ Hace menos de 2 semanas

2 ❑ 2 a 4 semanas atrás

3 ❑ Más de 4 semanas atrás

4 ❑ No recuerdo

**B.** Si NO fuma actualmente**,** ¿Cuántos cigarrillos fumaba al día?

1 ❑ 1- 5 3 ❑ 11-20

2 ❑ 6-10 4 ❑ Más de 20

1 ❑ Sí:

**A.** ¿Cuántos cigarrillos fuma al día?

1 ❑ 1- 5 3 ❑ 11-20

2 ❑ 6-10 4 ❑ Más de 20

**98. Desde la última vez que la vimos/hablamos durante su primer/segundo trimestre**, **el/en (dar fecha),** sin incluir cigarrillos electrónicos, ¿Alguna otra persona que vive en su casa ha fumado cigarrillos, cigarros o pipas dentro de la casa?

0 ❑ No (**SKIP TO #101)**

1 ❑ Si

**99.** **Desde la última vez que la vimos/hablamos durante su primer/segundo trimestre**, **el/en (dar fecha),** ¿Quién más en su casa ha fumado cigarrillos, cigarros o pipas?  **(MARK ALL THAT APPLY)**

1 ❑ Padre del bebe

2 ❑ Otras personas

**100. Desde la última vez que la vimos/hablamos durante su primer/segundo trimestre**, **el/en (dar fecha),** ¿Cuántas personas viviendo en su casa fuman cigarrillos, cigarros o pipas?

1 ❑ 1

2 ❑ 2

3 ❑ 3

4 ❑ 4 o más

**101.** **Desde la última vez que la vimos/hablamos durante su primer/segundo trimestre**, **el/en (dar fecha),** *en un promedio*, ¿Cuántas horas al día está expuesta usted al humo de cigarrillos, cigarros o pipas fumados por otras personas?

1 ❑ 0-1 hora

2 ❑ 1-2 horas

4 ❑ 3-4 horas

5 ❑ Más de 4 horas

3 ❑ 2-3 horas

**102.** ¿Ha fumado usted cigarrillos electrónicos o algún otro sistema electrónico de administración de nicotina (e-hookah, e-cigars, etc.)?

0 ❑ No (**SKIP TO #105**)

1 ❑ Sí

**103.** **Desde la última vez que la vimos/hablamos durante su primer/segundo trimestre**, **el/en (dar fecha),** ¿Ha fumado usted cigarrillos electrónicos o algún otro sistema electrónico de administración de nicotina (e-hookah, e-cigars, etc.)?

0 ❑ No **(SKIP TO #105)**

1 ❑ Sí

**104.** ¿Ha fumado usted cigarrillos electrónicos o algún otro sistema electrónico de administración de nicotina (e-hookah, e-cigars, etc.) en los últimos 5 días?

0 ❑ No:

**A.** Si NO fuma actualmente**,** ¿cuándo dejo de fumar? [**MARK ONE**]

1 ❑ Hace menos de 2 semanas

2 ❑ 2 a 4 semanas atrás

3 ❑ Más de 4 semanas atrás

4 ❑ No recuerdo

1. Si NO fuma actualmente**,** ¿Qué tan seguido fumaba cigarrillos electrónicos o algún otro sistema electrónico de administración de nicotina (e-hookah, e-cigars, etc.)?

1❑ Todos los días

2❑ Cada dos o tres días

3❑ Una vez a la semana

4❑ Aproximadamente una vez al mes

5❑ Cada cuantos meses

1 ❑ Sí:

**A.** ¿Qué tan seguido fuma cigarrillos electrónicos o algún otro sistema electrónico de administración de nicotina (e-hookah, e-cigars, etc.)?

1❑ Todos los días

2❑ Cada dos o tres días

3❑ Una vez a la semana

4❑ Aproximadamente una vez al mes

5❑ Cada cuantos meses

**CARACTERISTICAS DEL HOGAR**

******For Administrator Only (Do not ask participant):**

Did participant move since last seen on 10-27-2015?

0 ❑ No… Ask 107, 108, 110, 113-119, 121-122

1 ❑ Yes…Ask 105-122

**105**. **¿Cuál opción describe mejor la casa en la cual reside actualmente la mayor parte del tiempo?** *Marque una sola respuesta.*

1 ❑ Una casa (que no está unida a otras casas)

2 ❑ Un edificio de 2-4 departamentos unidos, townhome, condominio, dúplex o triplex

3 ❑ Un edificio de 5-10 departamentos unidos, townhome, condominio, etc.

4 ❑ Un edificio de más de 10 departamentos unidos, townhome, condominio, etc.

5 ❑ Una casa móvil (“mobile home”) o en un tráiler

6 ❑ Otro, por favor explique: ___________________________________________________

**106**. **Aproximadamente, ¿cuándo fue esta vivienda originalmente construida? (cuando se construyó por primera vez, no cuando pudo haber sido remodelada o modificada). *[****Mark one]*

1❑ 2000s o más reciente

2❑ 1980s-1990s

3❑ 1960s-1970s

4❑ 1940s-1950s

5❑ Antes de 1940

**107.** **Desde la última vez que la vimos/hablamos durante su primer/segundo trimestre**, **el/en (dar fecha),** ¿Cuáles de las siguientes mascotas ha tenido/tiene dentro de su casa? **(MARK ALL THAT APPLY)**

1 ❑ No tengo mascotas

2 ❑ Perro(s)

3 ❑ Gato(s)

4 ❑ Otras mascotas (Explique: ____________)

**108.** **Desde la última vez que la vimos/hablamos durante su primer/segundo trimestre**, **el/en (dar fecha),** ¿Ha tenido Usted alguna de las siguientes infestaciones en su casa? **(MARK ALL THAT APPLY)**

1 ❑ Ratas

2 ❑ Ratones

3 ❑ Cucarachas

4 ❑ Otras infestaciones (Explique:____________)

5 ❑ No sé

6 ❑ Ningún problema con infestaciones

**109**. ¿Tiene en su casa una estufa u horno de GAS?

0 ❑ No

1 ❑ Sí:

**A.** ¿Con que frecuencia usa la estufa u horno mientras que usted está en casa? *Mark one.*

1 ❑ Nunca **(SKIP to 110)**

2 ❑ Menos de una vez por semana

3 ❑ 1-3 veces por semana

4 ❑ 4-7 veces por semana

5 ❑ 8-14 veces por semana

6 ❑ Más de 14 veces por semana

**B.** En promedio, ¿Por cuánto tiempo se usa la estufa u horno durante el día mientras que usted está en casa?

1 ❑ Menos de 15 minutos

2 ❑ 15 minutos a menos de 30 minutos

3 ❑ 30 minutos a menos de 1 hora

4 ❑ 1 hora o más

**110.** **Desde la última vez que la vimos/hablamos durante su primer/segundo trimestre**, **el/en (dar fecha),** en promedio, ¿cuántas veces a la semana cocina usted (usando la estufa /horno, no incluyendo el uso del microondas)?

_1_❑ Nunca _3_❑ 4 – 5 veces por semana

_2_❑ 1 – 3 veces por semana _4_❑ Todos los días

**111.** ¿Su casa tiene sistema de calefacción o calentón?

0 ❑ No **(SKIP to 113)**

1 ❑ Sí:

**A.** ¿Cuál es el principal combustible utilizado para calentar la casa? *Mark one.*

1 ❑ Gas (podrá ver una llama azul o el piloto encendido dentro de la unidad)

2 ❑ Eléctrico (podrá ver un alambre o metal ardiente dentro de la unidad)

3 ❑ Un tanque de gas (un tanque o cilindro fuera de la casa que se puede llenar de gas)

4 ❑ Leña

5 ❑ Otro, por favor explique: ________________________

9 ❑ No sé cómo se calienta

**112.** ¿Cuál es el principal sistema de calefacción en su casa? *Mark one.*

1 ❑ Aire forzado

2 ❑ Unidad eléctrica pegada a la pared

3 ❑ Calentón ubicado en la pared

4 ❑ Calentón ubicado en el piso

5 ❑ Calentador portátil…**¿Qué tipo?**

1 ❑ Gas

2 ❑ Eléctrico

3 ❑ No sé

6 ❑ Otro, por favor explique: ______________________________

9 ❑ No sé cómo se calienta

**113.** ¿Usa usted aire acondicionado en su casa?

0 ❑ No **(SKIP to 116)**

1 ❑ Sí: **A.** ¿Cuál es el tipo principal de aire acondicionado que se utiliza? *Mark one.*

1 ❑ Aire acondicionado de ventana o pared (caja que sale de la ventana o pared)

**a.** ¿Cuántas unidades de ventana/pared tiene usted en su casa?

_1_ ❑ Uno

_2_ ❑ Dos

_3_ ❑ Tres

_4_ ❑ Cuatro o más

_5_ ❑ No sé

2 ❑ Central (escape de aire en las recamaras)

3 ❑ Enfriador de vapor (“swamp cooler”)

9 ❑ No sé qué tipo sea

**114**. **Durante el último mes,** ¿con que frecuencia uso el aire acondicionado estando en casa?

1 ❑ Nunca

2 ❑ Menos de 5 días

3 ❑ 5-15 días

4 ❑ 16-30 días

9 ❑ No sé

**115.** En un día cualquiera, ¿cuánto tiempo uso usted el aire acondicionado en su casa?

_1_ ❑ Nunca

_2_ ❑ Un par de horas al día

_3_ ❑ La mitad del tiempo

_4_ ❑ La mayor parte del tiempo

_5_ ❑ Todo el tiempo

_9_ ❑ No sé

**116.** **Durante el último mes,** ¿usó usted un ventilador de ventana o algún otro ventilador que puso en la ventana o en el ático para enfriar su casa?

0 ❑ No

1 ❑ Sí

**117.** **Desde la última vez que la vimos/hablamos durante su primer/segundo trimestre**, **el/en (dar fecha),** ¿ha habido algún tipo de daño causado por agua o inundación en su casa?

0 ❑ No

1 ❑ Sí:

**A.** ¿Inundó áreas alfombradas?

0 ❑ No

1 ❑ Sí

9 ❑ No sé

**118.** **Desde la última vez que la vimos/hablamos durante su primer/segundo trimestre**, **el/en (dar fecha),** ¿se ha formado alguna vez moho en las paredes, techos, o pisos en su casa?

0 ❑ No

1 ❑ Sí:

**A.** ¿Qué cuartos quedaron afectados? *Mark all that apply.*

1 ❑ El cuarto donde duerme

2 ❑ Baño (s)

3 ❑ Sótano

4 ❑ Otro

9 ❑ No sé

**119.** ¿Se ha utilizado un humificador o vaporizador en su casa? (Incluyendo el humificador que puede tener dentro el sistema de calefacción.)

0 ❑ No

1 ❑ Sí:

**A.** ¿Qué tipo es? *Mark all that apply.*

1 ❑ Viene dentro el sistema de calefacción

2 ❑ Una unidad portátil

**B.** ¿Ha utilizado este aparato para tratar alguna enfermedad respiratoria?

0 ❑No

1 ❑Sí

**C.**  ¿Calienta el aire el humificador o vaporizador?

0 ❑No

1 ❑ Sí

9 ❑ No sé

**120**. ¿Hay alfombra en su casa?

0 ❑ No

1 ❑ Sí:

**A.** ¿En qué cuartos? *Mark all that apply.*

1 ❑ Toda la casa (excluyendo la cocina y baño)

2 ❑ Recamara donde duerme

3 ❑ Otras recamara(s)

4 ❑ Otros cuarto(s)

**121.** Recordando un día típico entresemana de la **semana pasada**, aproximadamente cuantas horas (de 24 horas en total) estuvo…

**A.** Afuera: ________________

**B.** Adentro de la casa (Incluyendo la noche/durmiendo):____________________

**122.** En promedio, ¿cuánto tiempo mantuvo las ventanas abiertas durante esta última **semana**?

_1_ ❑ Nunca

_2_ ❑ Un par de horas al día

_3_ ❑ La mitad del tiempo

_4_ ❑ La mayor parte del tiempo

_5_ ❑ Todo el tiempo

_9_ ❑ No sé

**PREGUNTAS SOBRE COMO DUERME**

**A continuación, vamos a preguntarle acerca de sus hábitos de dormir durante el mes pasado (30 días). Piense en el mes pasado (30 días).**

**123. En el mes pasado,** por lo general, ¿cuántas horas durmió durante una noche típica de entresemana (domingo – jueves)?

_1_ ❑ Menos de 4 horas por noche _5_ ❑ 8 horas por noche

_2_ ❑ 5 horas por noche _6_ ❑ 9 horas por noche

_3_ ❑ 6 horas por noche _7_ ❑ Más de 10 horas por noche

_4_ ❑ 7 horas por noche

**124.** **En el mes pasado,** por lo general, ¿cuántas horas durmió durante una noche típica de fin de semana (viernes o sábado)?

_1_ ❑ Menos de 4 horas por noche _5_ ❑ 8 horas por noche

_2_ ❑ 5 horas por noche _6_ ❑ 9 horas por noche

_3_ ❑ 6 horas por noche _7_ ❑ Más de 10 horas por noche

_4_ ❑ 7 horas por noche

**Questions 125-128 Jenkins Sleep Questionnaire**

Jenkins CD, Stanton B-A, Niemcrym SJ, Rose RM. A scale for the estimation of sleep problems in clinical research. J Clin Epidemiol 1988;41:313-21.

**129.** **Durante el AÑO PASADO, en promedio, ¿cuántas veces ha roncado o le han dicho que ronca cuando duerme? (MARK ONE)**

_1_ ❑ Nunca

_2_ ❑ Raramente (Menos de una vez por semana)

_3_ ❑ Abecés (1 to 2 veces por semana)

_4_ ❑ Frecuentemente (3 to 4 veces por semana)

_5_ ❑ Siempre/Casi siempre (5 to 7 veces por semana)

_9_ ❑ No sé

**LUGAR DE NACIMIENTO DE SUS PADRES**

**130.** ¿Nació el padre de usted en los EE.UU?

0 ❑ No…**A**. ¿En qué país nació el padre de usted?_____________________________

1 ❑ Sí…**B**. ¿En qué ciudad nació el padre de usted? _____________________________

**C**. ¿En qué estado nació el padre de usted? _____________________________

**131**. ¿Nació la madre de usted en los EE.UU?

0 ❑ No…**A.** ¿En qué país nació la madre de usted?_____________________________

1 ❑ Sí…**B**. ¿En qué ciudad nació la madre de usted? _____________________________

**C**. ¿En qué estado nació la madre de usted? _____________________________

**PLAN DE NACIMIENTO**

**132.** ¿Dónde planea dar a luz?

_1_ ❑ LAC+USC

_2_ ❑ White Memorial Medical Center

_3_ ❑ California Hospital Medical Center

_4_ ❑ Good Samaritan Hospital

_5_ ❑ En casa

_6_ ❑ Otro, por favor explique: ______________________________

_9_ ❑ No sé

**133**. ¿Tendrá un parto planeado/cesárea?

0 ❑ No

1 ❑ Sí:

A. ¿Para cuándo está programado/a el parto/cesaría?

Date:_____/_____/_____

Hora: ______________
