# Supplementary material for: Study Design, Protocol and Profile of the Maternal And Developmental Risks from Environmental and Social Stressors (MADRES) Pregnancy Cohort: a Prospective Cohort Study in Predominantly Low-Income Hispanic Women in Urban Los Angeles
Source: BMC Pregnancy Childbirth. 2019 May 30;19:189. doi: 10.1186/s12884-019-2330-7 (PMC6543670; doi:10.1186/s12884-019-2330-7)
Supplement: Supplementary file 13 — MADRES Late Entry Questionnaire. Questionnaire administered during the third trimester for participant recruited between 20 and 30 weeks of pregnancy. (DOCX 144 kb) [file 12884_2019_2330_MOESM13_ESM.docx]

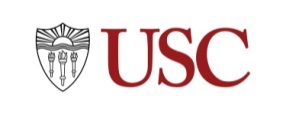
 **MADRES Study: Late Entry Questionnaire**

**Today’s Date:** _____________________ **Interviewer Name:** ____________________

**Instructions:** Thank you for agreeing to participate in this study. In this interview, I will be asking some questions about you and your health history. Please answer all questions as best as you can, even if you are not completely certain. Be assured that your answers are confidential. Please feel free to interrupt me and ask about anything that is not clear**.**

**CONTACT INFORMATION**

**1. Name:** _________________ _______________ ____________________ ____________________

First Middle Last 1 Last 2

**2. Other names used** (e.g. Maiden name) ­­­­­­­­­­­­­­­­­­­­­­­­­:­___________________________

### **3**. **Your Date of Birth:** **_______/_______/_______**

Month Day Year

### **4**. **Estimated** **Due Date:** **_______/_______/_______**

Month Day Year

### **5.** **Last Menstrual Period:** **_______/_______/_______**

Month Day Year

**6. What is your cell phone number?** ____________________________

□₀ Don’t have a cell phone **(Skip to question #8)**

**7.** **Is this a prepaid cell phone or a permanent phone number?**

□₀ prepaid

□₁ permanent number

**8.** **What is your HOME address (the address at which you spend the most time)?**

Address: ______________________________________________________________________

City: ___________________________State: ________________Zip: _____________________

**8A.** **If moved…When did you move into your new home address?** _______________________

**9A. Please tell me the names of other adults living with you:**

Adult#1 First: ______________________Last: ______________________Middle: ______________

Relation to you: ___________________ Cell Phone: ______________________

Adult#2 First: ______________________Last: ______________________Middle: ______________

Relation to you: ___________________ Cell Phone: ______________________

Adult#3 First: ______________________Last: ______________________Middle: ______________

Relation to you: ___________________ Cell Phone: ______________________

**10. What is the phone number for the HOME listed in Question 8?** ____________________________ □₀ Don’t have a home phone

**11. Do you live at more than one home?**

□₁ Yes... *Complete questions 12A, 12B and 12C* □₀No… *Go to Question #13*

**12A. What is your second HOME address?**

Address: ______________________________________________________________________

City: ___________________________State: ________________Zip: _____________________

**12A2.** **If moved…When did you move into your new second home address?**  ___________________

**12B. What is the phone number for the HOME listed in Question 12A?** _______________________

□₀ Don’t have a home phone

**12C. How much time do you spend at the address listed in 12A?**

 1%-25% of the time

 26%-50% of the time

**13. A. What is your email address?** _________________________ 0 ❑Don’t have an email address

**B. What is your Facebook username?** ___________________________0 ❑Don’t have Facebook

**C. What is your Twitter handle?** @________________________________0 ❑Don’t have Twitter

**D. What is your Instagram contact name?** _______________________0 ❑Don’t have Instagram

**14.** **A. How do you prefer to be contacted?**

 Phone

 Email

 Text

 Other: ________________

**B. What are the best days to reach you?**

 Monday

 Tuesday

 Wednesday

 Thursday

 Friday

 Saturday

 Sunday

**C. What are the best times to reach you (Monday)?**

 Mornings (8am-12pm)

 Afternoons (12pm-5pm)

 Evenings (5pm-8pm)

 Other: ________________

**D. What are the best times to reach you (Tuesday)?**

 Mornings (8am-12pm)

 Afternoons (12pm-5pm)

 Evenings (5pm-8pm)

 Other: ________________

**E. What are the best times to reach you (Wednesday)?**

 Mornings (8am-12pm)

 Afternoons (12pm-5pm)

 Evenings (5pm-8pm)

 Other: ________________

**F. What are the best times to reach you (Thursday)?**

 Mornings (8am-12pm)

 Afternoons (12pm-5pm)

 Evenings (5pm-8pm)

 Other: ________________

**G. What are the best times to reach you (Friday)?**

 Mornings (8am-12pm)

 Afternoons (12pm-5pm)

 Evenings (5pm-8pm)

 Other: ________________

**H. What are the best times to reach you (Saturday)?**

 Mornings (8am-12pm)

 Afternoons (12pm-5pm)

 Evenings (5pm-8pm)

 Other: ________________

**I. What are the best times to reach you (Sunday)?**

 Mornings (8am-12pm)

 Afternoons (12pm-5pm)

 Evenings (5pm-8pm)

 Other: ________________

**15.** **What is the baby’s father’s name?** □ Don’t know

_________________ _______________ ____________________ ____________________

First Middle Last 1 Last 2

**16A. Do you have a spouse/partner?** 0 ❑ No…Go to Question 17 1 ❑ Yes

**16B. What is the name of your spouse/partner**? □ Same as above □ No spouse/partner

_________________ _______________ ____________________ ____________________

First Middle Last 1 Last 2

**17.** **In order to help locate you in case you move and/or change your phone number can you provide us with contact information for your mother and three friends/family members not living with you who would be able to provide us with your new contact information?**

YOUR MOTHER’S INFORMATION

First: ______________________Last: ______________________Middle: ______________

Address: ______________________________________________________________________

City: ___________________________State: ________________Zip: _____________________

Cell Phone: ______________________ Home Phone: ______________________

NOK#1

First: ______________________Last: ______________________Middle: ______________

Relation to you: ___________________Email address: ____________________________

Cell Phone: ______________________ Home Phone: ______________________

NOK#2

First: ______________________Last: ______________________Middle: ______________

Relation to you: ___________________Email address: ____________________________

Cell Phone: ______________________ Home Phone: ______________________

NOK#3

First: ______________________Last: ______________________Middle: ______________

Relation to you: ___________________Email address: ____________________________

Cell Phone: ______________________ Home Phone: ______________________

**MAILING ADDRESS**

**18. Do you have a P.O. Box or a mailing address that is different than your home address?**

0 ❑ No

1 ❑ Yes…what is your P.O. Box or mailing address?

Address: _________________________________________________________________

City: ___________________________State: ________________Zip: ________________

**DEMOGRAPHIC DATA**

**19**. Are you of Hispanic or Latino ethnicity?

0 ❑ No

1 ❑ Yes

**20.** Is the baby’s biological father of Hispanic or Latino ethnicity?

0 ❑ No

1 ❑ Yes

9 ❑ Don’t Know

**21**. What is your race? (**MARK ALL THAT APPLY**):

1 ❑ White

2 ❑ Asian

3 ❑ Black or African-American

4 ❑ Native Hawaiian or Pacific Islander

5 ❑ American Indian/Alaska Native

6 ❑ Other: Explain: _________________

**22.** What is your baby’s biological father’s race? (**MARK ALL THAT APPLY**)

1 ❑ White 4 ❑ Native Hawaiian or Pacific Islander

2 ❑ Asian 5 ❑ American Indian/Alaska Native

3 ❑ Black or African-American 6 ❑ Other: Explain: _________________

9 ❑ Don’t Know

**23.** Were you born in the USA?

1 ❑ Yes

0 ❑ No… **A**. **Where were you born? ________________________________**

**B. How long have you lived in the USA? _____________**

**24.** Was your baby’s biological father born in the USA?

1 ❑ Yes

0 ❑ No…

1. **Where was he born? ____________________________________________**
2. **How long has he lived in the USA? _____________** ❑**Never lived in USA**

9 ❑ Don’t Know

**25.** What is your marital status? **[MARK ONE]**

1 ❑ Married

2 ❑ Living together

3 ❑ Never married, single

4 ❑ Divorced or separated

5 ❑ Widowed

6 ❑ Decline to answer

**26.** What was the last grade in school **you** completed? **[MARK ONE]**

1 ❑ Less than 12th grade (did not finish high school)

2 ❑ Completed grade 12 (high school)

3 ❑ Some college or technical school

4 ❑ Completed 4 years of college

5 ❑ Some graduate training after college

**27.** What was the last grade in school **the baby’s biological father** completed? **[MARK ONE]**

1 ❑ Less than 12th grade (did not finish high school)

2 ❑ Completed grade 12 (high school)

3 ❑ Some college or technical school

4 ❑ Completed 4 years of college

5 ❑ Some graduate training after college

9 ❑ Don’t know

**PRE-PREGNANCY PHYSICAL ACTIVITY AND WEIGHT**

**28.** What was your weight in pounds before this pregnancy? **__________________**

**29.** The next question will ask about how much physical activity per week you typically did before you were pregnant. (*Physical Activity is any activity that increases your heart rate and makes you get out of breath some of the time.* *Physical activity can be done through sports, exercise, classes, housework, yardwork, travelling to get somewhere. Some examples of physical activity are running, brisk walking, biking, dancing, swimming, yoga, aerobics classes, and mowing the lawn.)*

Please think back to a typical week **during the 6 months before you became pregnant**. On how many days per week were you physically active for a total of at least 30 minutes, not necessarily consecutive, per day? (Please check one)

1❑ 0 days per week 5❑ 4 days per week

2❑ 1 day per week 6❑ 5 days per week

3❑ 2 days per week 7❑ 6 days per week

4❑ 3 days per week 8❑ 7 days per week

**PREGNANCY INFORMATION**

**30.** What is the birth order of this baby?

1 ❑ 1 (first-born)

2 ❑ 2 (second-born)

3 ❑ 3 (third-born)

4 ❑ 4 (fourth-born)

5 ❑ 5 (fifth-born)

6 ❑ 6 or more (sixth-born or later)

**31.** Have you ever had hay fever also known as allergic rhinitis? (An allergic reaction causing nasal congestion, runny nose, sneezing, nose and eye itching, or excessive tear production when you did not have a cold or the “flu”.)

0 ❑ No

1 ❑ Yes

9 ❑ Don’t know

**32.** Do you have allergies?

0 ❑ No

1 ❑ Yes…what are you allergic to?  MARK ALL THAT APPLY.

1 ❑ Dogs

2 ❑ Cats

3 ❑ Other pets

4 ❑ Foods

5 ❑ Plants or pollen

6 ❑ Mold

7 ❑ Other (explain): ____________________

**INCOME**

**33.** In which of the following categories did your TOTAL HOUSEHOLD FAMILY INCOME fall in last year? Include all incomes, before taxes and deductions, of all members of the family.

_1_ ❑ Less than $15,000 _4_ ❑ $50,000 to $99,999

_2_ ❑ $15,000 to $29,999 _5_ ❑ $100,000 or more

_3_ ❑ $30,000 to $49,999 _9_ ❑ Don’t know

**FATHER’S INFORMATION**

**34.** Do you know your baby’s father’s DOB?

0 ❑ No

1 ❑ Yes… A. What is your baby’s father’s DOB? _______/_______/_______

Month Day Year

**35.** Is the baby’s biological father still alive?

1❑ Yes

9❑ Don’t know

0❑ No…

1. **Do you know approximately how old your baby’s biological father was when he died?**

0 ❑ No

1 ❑ Yes… **B. Approximately how old was your baby’s biologic father when he died?**

_______ years

**C. What was the cause of death?**

1❑ Accident

2❑ Cancer

3❑ Heart attack

4❑ Stroke

5❑ Other specify:______________

9❑ Don’t know

**36.** Does/did your baby’s father ever have diabetes?

0 ❑ No

1 ❑ Yes, Type I

2 ❑ Yes, Type II

3 ❑ Yes but don’t know type

9 ❑ Don’t know

**37.** Does/did your baby’s father ever take medication to lower cholesterol or lipids?

0 ❑ No

1 ❑ Yes

9 ❑ Don’t know

**38.** Is/was your baby’s father very overweight?

0 ❑ No

1 ❑ Yes

9 ❑ Don’t know

**39.** Do you know what your baby’s father height is/was?

0 ❑ No

1 ❑ Yes… **A.** What is/was your baby’s father current height? _____ft ______in

**40.** Do you know what your baby’s father weight is/was?

0 ❑ No

1 ❑ Yes… **A.** What is/was your baby’s father current weight? _________pounds

**STRESS EVALUATION**

**Questions 41-50 Perceived Stress Scale**

Cohen S, Kamarck T, Mermelstein R: **A global measure of perceived stress**. *J Health Soc Behav* 1983, **24**(4):385-396.

**Questions 51-70 CES-D Scale**

Radloff LS: **The CES-D scale: A self report depression scale for research in the general population.** *Applied Psychological Measurements* 1977, **1**:385-401.

**Questions 71-87 The Prenatal Distress Questionnaire**

Yali AM, Lobel M: **Coping and distress in pregnancy: an investigation of medically high risk women**. *J Psychosom Obstet Gynaecol* 1999, **20**(1):39-52.

**STRESSFUL LIFE EVENTS OVER PREGNANCY**

**Questions 88 (88a-88m)**

Life course indicator: stressors during pregnancy (LC-56) Association of Maternal and Child Health Programs, <http://www.amchp.org/programsandtopics/data-assessment/LifeCourseIndicatorDocuments/LC-56_Stressors%20During%20Pregnancy_Final-12-16-2013.pdf>

**OCCUPATIONAL HISTORY**

**89**. What is your current employment status? (**MARK ALL THAT APPLY**)

1 ❑ Homemaker

2 ❑ Student

3 ❑ Employed

4 ❑ Temporary medical leave

5 ❑ Unemployed

6 ❑ Other: Explain: ____________________

**90.** Have you been working **in the last month**?

0 ❑ No (**SKIP TO #94**)

1 ❑ Yes

**91.** How many hours do you work per week?

1 ❑ Less than 10 hours/week

2 ❑ 10-20 hours/week

3 ❑ 21-30 hours/week

4 ❑ 31-40 hours/week

5 ❑ More than 40 hours/week

**92**. During a regular work week, how many days do you commute to and from your work location?

0 ❑ 0 days

1 ❑ 1 days

2 ❑ 2 days

3 ❑ 3 days

4 ❑ 4 days

5 ❑ 5 days

6 ❑ 6 days

7 ❑ 7 days

**93.** Thinking about your typical commute **TO** your job, which forms of transportation do you use and for how long? (Mark all that apply.)

|  | **1-10**  **Minutes** | **11-20**  **Minutes** | **21-30**  **Minutes** | **31-59**  **Minutes** | **60-90**  **Minutes** | **91-120**  **Minutes** | **2 Hours or More** | **N/A** |
| --- | --- | --- | --- | --- | --- | --- | --- | --- |
| Car |  |  |  |  |  |  |  |  |
| Bus or  Tram |  |  |  |  |  |  |  |  |
| Train or Metro |  |  |  |  |  |  |  |  |
| By motorcycle |  |  |  |  |  |  |  |  |
| By bike |  |  |  |  |  |  |  |  |
| On foot |  |  |  |  |  |  |  |  |

**PREGNANCY INFORMATION**

**94.** **In the last month,** have you taken any multivitamins or prenatal vitamins?

0 ❑ No

1 ❑ Yes:

**A.** How many vitamin tablets do you take?

1 ❑ 1 to 3 per week

2 ❑ 4 to 6 per week

3 ❑ 1 per day

4 ❑ More than 1 per day

9 ❑ Don’t remember

**B.** Does your vitamin tablet contain folic acid?

0 ❑ No

1 ❑ Yes

9 ❑ Don’t know

**95. In the last month,** have you taken an individual folic acid tablet?

0 ❑ No

1 ❑ Yes

**A.** How many folic acid tablets do you take?

1 ❑ 1 to 3 per week

2 ❑ 4 to 6 per week

3 ❑ 1 per day

4 ❑ More than 1 per day

9 ❑ Don’t remember

9❑ Don’t know

**96.** Did you eat any of the following types of seafood **in the last month**?

|  | **Did you eat the following types of seafood in the last month?** | **If yes, how often?** | **If yes, did you eat this in the past three days?** |
| --- | --- | --- | --- |
| Fish sticks (any commercially prepared frozen, boneless white fish, crumbled and fried in oil) | 0 ❑ No  1 ❑ Yes  9 ❑ Don’t know | 1 ❑ Daily  2 ❑ Weekly  3 ❑ Monthly  4 ❑ Rarely  9 ❑ Don’t know | 0 ❑ No  1 ❑ Yes |
| Fresh oily fish (containing more than 2% fat: blue-eye cod, silver bream, gemfish, blackfish, mullet, orange roughy, pilchards, redfish, yellowtail, Atlantic salmon, southern bluefin tuna, blue grenadier, tailor, blue mackerel, tarwhine, rainbow trout) | 0 ❑ No  1 ❑ Yes  9 ❑ Don’t know | 1 ❑ Daily  2 ❑ Weekly  3 ❑ Monthly  4 ❑ Rarely  9 ❑ Don’t know | 0 ❑ No  1 ❑ Yes |
| Other fresh fish not listed above (non-oily fish: flounder, shark) | 0 ❑ No  1 ❑ Yes  9 ❑ Don’t know | 1 ❑ Daily  2 ❑ Weekly  3 ❑ Monthly  4 ❑ Rarely  9 ❑ Don’t know | 0 ❑ No  1 ❑ Yes |
| Canned tuna | 0 ❑ No  1 ❑ Yes  9 ❑ Don’t know | 1 ❑ Daily  2 ❑ Weekly  3 ❑ Monthly  4 ❑ Rarely  9 ❑ Don’t know | 0 ❑ No  1 ❑ Yes |
| Fried shellfish (shrimp, lobster, crabs, crayfish, clams, mussels, scallops) | 0 ❑ No  1 ❑ Yes  9 ❑ Don’t know | 1 ❑ Daily  2 ❑ Weekly  3 ❑ Monthly  4 ❑ Rarely  9 ❑ Don’t know | 0 ❑ No  1 ❑ Yes |
| Shellfish (shrimp, lobster, crabs, crayfish, clams, mussels, scallops) | 0 ❑ No  1 ❑ Yes  9 ❑ Don’t know | 1 ❑ Daily  2 ❑ Weekly  3 ❑ Monthly  4 ❑ Rarely  9 ❑ Don’t know | 0 ❑ No  1 ❑ Yes |

**97.** Did you drink caffeinated coffee or caffeinated tea **in the last month**?

0 ❑ No

1 ❑ Yes:

**A.** How often did you drink caffeinated coffee or caffeinated tea **in the last month**?

1 ❑ 1 to 3 drinks per week

2 ❑ 4 to 6 drinks per week

3 ❑ 1-2 drinks per day

4 ❑ 3-4 drinks per day

1. ❑ More than 4 drinks per day

9❑ Not sure

**98.** Did you drink any of the following beverages **in the last month**?

| **Beverage** | **Did you drink it in the last month?** | **If yes, how often?** |
| --- | --- | --- |
| Aguas frescas (ex: Horchata, Tamarindo, Watermelon water) | 0 ❑ No  1 ❑ Yes | 1 ❑ 1 to 3 drinks per week  2 ❑ 4 to 6 drinks per week  3 ❑ 1-2 drinks per day  4 ❑ 3-4 drinks per day  5 ❑ More than 4 drinks per day  9 ❑ Not sure |
| Caffeinated Soda (Ex: Coca-Cola, Pepsi, Mountain Dew) | 0 ❑ No  1 ❑ Yes | 1 ❑ 1 to 3 drinks per week  2 ❑ 4 to 6 drinks per week  3 ❑ 1-2 drinks per day  4 ❑ 3-4 drinks per day  5 ❑ More than 4 drinks per day  9 ❑ Not sure |
| Decaffeinated Soda (ex: Sprite, 7-UP) | 0 ❑ No  1 ❑ Yes | 1 ❑ 1 to 3 drinks per week  2 ❑ 4 to 6 drinks per week  3 ❑ 1-2 drinks per day  4 ❑ 3-4 drinks per day  5 ❑ More than 4 drinks per day  9 ❑ Not sure |
| Champurrado | 0 ❑ No  1 ❑ Yes | 1 ❑ 1 to 3 drinks per week  2 ❑ 4 to 6 drinks per week  3 ❑ 1-2 drinks per day  4 ❑ 3-4 drinks per day  5 ❑ More than 4 drinks per day  9 ❑ Not sure |
| Hot Chocolate / Chocolate Abuelita | 0 ❑ No  1 ❑ Yes | 1 ❑ 1 to 3 drinks per week  2 ❑ 4 to 6 drinks per week  3 ❑ 1-2 drinks per day  4 ❑ 3-4 drinks per day  5 ❑ More than 4 drinks per day  9 ❑ Not sure |
| Energy Drinks (ex: Red Bull, Rockstar, Monster) | 0 ❑ No  1 ❑ Yes | 1 ❑ 1 to 3 drinks per week  2 ❑ 4 to 6 drinks per week  3 ❑ 1-2 drinks per day  4 ❑ 3-4 drinks per day  5 ❑ More than 4 drinks per day  9 ❑ Not sure |
| Flavored Coffee | 0 ❑ No  1 ❑ Yes | 1 ❑ 1 to 3 drinks per week  2 ❑ 4 to 6 drinks per week  3 ❑ 1-2 drinks per day  4 ❑ 3-4 drinks per day  5 ❑ More than 4 drinks per day  9 ❑ Not sure |
| Flavored Milk (ex: chocolate milk, strawberry milk) | 0 ❑ No  1 ❑ Yes | 1 ❑ 1 to 3 drinks per week  2 ❑ 4 to 6 drinks per week  3 ❑ 1-2 drinks per day  4 ❑ 3-4 drinks per day  5 ❑ More than 4 drinks per day  9 ❑ Not sure |
| Fruit-flavored Drinks (ex: Snapple) | 0 ❑ No  1 ❑ Yes | 1 ❑ 1 to 3 drinks per week  2 ❑ 4 to 6 drinks per week  3 ❑ 1-2 drinks per day  4 ❑ 3-4 drinks per day  5 ❑ More than 4 drinks per day  9 ❑ Not sure |
| Jarritos | 0 ❑ No  1 ❑ Yes | 1 ❑ 1 to 3 drinks per week  2 ❑ 4 to 6 drinks per week  3 ❑ 1-2 drinks per day  4 ❑ 3-4 drinks per day  5 ❑ More than 4 drinks per day  9 ❑ Not sure |
| Juice (ex: orange juice, apple juice, cranberry juice) | 0 ❑ No  1 ❑ Yes | 1 ❑ 1 to 3 drinks per week  2 ❑ 4 to 6 drinks per week  3 ❑ 1-2 drinks per day  4 ❑ 3-4 drinks per day  5 ❑ More than 4 drinks per day  9 ❑ Not sure |
| Powdered-drinks (ex: Tang, Kool-Aid) | 0 ❑ No  1 ❑ Yes | 1 ❑ 1 to 3 drinks per week  2 ❑ 4 to 6 drinks per week  3 ❑ 1-2 drinks per day  4 ❑ 3-4 drinks per day  5 ❑ More than 4 drinks per day  9 ❑ Not sure |
| Sports Drinks (ex: Gatorade) | 0 ❑ No  1 ❑ Yes | 1 ❑ 1 to 3 drinks per week  2 ❑ 4 to 6 drinks per week  3 ❑ 1-2 drinks per day  4 ❑ 3-4 drinks per day  5 ❑ More than 4 drinks per day  9 ❑ Not sure |
| Sweeten Tea (hot or cold) | 0 ❑ No  1 ❑ Yes | 1 ❑ 1 to 3 drinks per week  2 ❑ 4 to 6 drinks per week  3 ❑ 1-2 drinks per day  4 ❑ 3-4 drinks per day  5 ❑ More than 4 drinks per day  9 ❑ Not sure |

**99.** Did you eat **rice** **in the last month**?

0 ❑ No

1 ❑ Yes: A. How often did you eat **in the last month**?

1 ❑ 1-6 times per year 6 ❑ 2 times per week

2 ❑ 7-11 times per year 7 ❑ 3-4 times per week

3 ❑ 1 time per month 8 ❑ 5-6 times per week

4 ❑ 2-3 times per month 9 ❑ 1 time per day

5 ❑ 1 time per week A ❑ 2 or more times per day

1. Each time you ate **rice** **in the last month**, how much did you usually eat?

1 ❑ Less than ½ cup 3 ❑ More than 1½ cups

2 ❑ ½ to 1½ cups

**100.** Did you eat **rice** **in the past three days**?

0 ❑ No

1 ❑ Yes: A. How often did you eat **rice** **in the past three days**?

1 ❑ 1 time per day 2 ❑2 or more times per day

B. Each time you ate **rice** **during the past three days**, how much did you usually eat?

1 ❑ Less than ½ cup

2 ❑ ½ to 1½ cups

3 ❑ More than 1½ cups

**101**. Has a doctor ever said you had asthma?

0 ❑ No (**SKIP TO #106**)

1 ❑ Yes:

**A.**  About how old were you when a doctor first said you had asthma?  Age: _______

**B.** Have you had problems with asthma DURING the time that you have been pregnant (even if you did not know that you were pregnant)?

0 ❑ No

1 ❑ Yes

**102. In the last month,** have you required medication for asthma or wheezing?

0 ❑ No

1 ❑ Yes

|  |  |  |  |  |
| --- | --- | --- | --- | --- |

**103.** **In the last month,** how often did you use albuterol (or other short-acting or “rescue” medication) or inhaled bronchodilators for asthma symptoms? **(Check only one)** *(If further probing is needed, examples include albuterol inhaler, Proventil inhaler, Ventolin inhaler, ProAir inhaler, Atrovent inhaler).*

1 ❑ Never

2 ❑ Less than two days a week

3 ❑ Two or more days a week (but not every day)

4 ❑ Once every day

5 ❑ More than once every day

**104. In the last month,** how often did you use inhaled corticosteroid medications or tablets (“controller” medications) to manage your asthma symptoms? *(Check only one).* *(If further probing is needed, examples include Advair, Beclovent Inhaler, Flovent Inhaler, Qvar Inhaler, Pulmicort Inhaler, Vanceril Inhaler, Intal Inhaler, Servent Inhaler, Singulair Tablets).*

1 ❑ Never

2 ❑ Less than two days a week

3 ❑ Two or more days a week (but not every day)

4 ❑ Once every day

5 ❑ More than once every day

**105. In the last month,** have you taken a course of steroid pills or liquids (i.e. Prednisone, Deltasone, Orasone, Prednicen-M, Liquid Pred) for your asthma symptoms? *A course is defined as one to eight consecutive days of either daily* ***or*** *alternate-day treatment.*

0 ❑ No

1 ❑ Yes

9 ❑ Don’t know

**106.** **In the last month,** have you taken any antibiotics?

0 ❑ No

1 ❑ Yes**… For each antibiotic you have taken while pregnant, please give me the name of the antibiotic, how long you took it, and for what illness it what taken.**

|  | **Antibiotic Name** | **Length of Time Taken during pregnancy** | **For what illness?** |
| --- | --- | --- | --- |
| **1** |  |  |  |
| **2** |  |  |  |
| **3** |  |  |  |
| **4** |  |  |  |

**107. In the last month,** have you taken any other medications prescribed by a doctor (not including birth control)?

0 ❑ No

1 ❑ Yes… **What other prescription medications did/do you take?**

______________________________________________________________

______________________________________________________________

**108.** **In the last month,** have you taken any over-the-counter medications (not including birth control) such as cold medicines, Tylenol, or Advil?

0 ❑ No

1 ❑ Yes… **What other over-the-counter medications did/do you take?**

1 ❑ Cold medicines

2 ❑ Tylenol/Acetaminophen

3 ❑ Advil/Ibuprofen

4 ❑ Other pain reliever: (Specify :________________)

6 ❑ Antacids (Tums, Rolaids, etc.)

5 ❑ Other over-the-counter-medication: (Specify :________________)

**109. In the last month,** have you taken any other natural remedies or traditional medicines to help with nausea symptoms (or “morning sickness”) from your pregnancy?

0 ❑ No

1 ❑ Yes … **What other remedies did/do you take?**

______________________________________________________________

______________________________________________________________

**110.** During this pregnancy, has a doctor told you that you have anemia or low hemoglobin in blood?

0 ❑ No 1 ❑ Yes

**111.** During this pregnancy, has a doctor told you that you have developed gestational diabetes?

0 ❑ No 1 ❑ Yes

**112.** During this pregnancy, has a doctor told you that you have developed preeclampsia or high blood pressure?

0 ❑ No 1 ❑ Yes

**113.** During this pregnancy, has a doctor told you about any other health conditions that you have developed?

0 ❑ No 1 ❑ Yes…***Please specify***: __________________________________

**Questions 114-116** Pregnancy-Unique Quantification of Emesis and Nausea

Koren G, Boskovic R, Hard M, Maltepe C, Navioz Y, Einarson A. Motherisk-PUQE (pregnancy-unique quantification of emesis and nausea) scoring system for nausea and vomiting of pregnancy. Am J Obstet Gynecol. 2002;186: S228–231.

**SMOKING QUESTIONS**

**117.** Excluding e-cigarettes, have you ever smoked cigarettes, cigars or pipes?

0 ❑ No (**SKIP TO #120**)

1 ❑ Yes

**118.** **In the last month,** excluding e-cigarettes, have you smoked cigarettes, cigars or pipes?

0 ❑ No (**SKIP TO #120**)

1 ❑ Yes

**119.** Have you smoked cigarettes, cigars, or pipes in the last 5 days?

0 ❑ No:

**A.** If you are NOT currently smoking, when did you stop smoking? [**MARK ONE**]

1 ❑ less than 2 weeks ago

2 ❑ 2 to 4 weeks ago

3 ❑ More than 4 weeks ago

4 ❑ Don't remember

**B.** If you are NOT currently smoking, how many cigarettes did you usually smoke *per day*?

1 ❑ 1- 5

2 ❑ 6-10

3 ❑ 11-20

4 ❑ More than 20

1 ❑ Yes:

**A.** How many cigarettes did you usually smoke *per day*?

1 ❑ 1- 5

2 ❑ 6-10

3 ❑ 11-20

4 ❑ More than 20

**120. In the last month,** excluding e-cigarettes, did anyone else living in your home smoke cigarettes, cigars or pipes inside the house?

0 ❑ No (**SKIP TO #123)**

1 ❑ Yes

**121.** **In the last month,** who else in your home smoked cigarettes, cigars or pipes**? (MARK ALL THAT APPLY)**

1 ❑ Baby's Father

2 ❑ Others

**122. In the last month,** not including yourself, how many people living in your home smoked cigarettes, cigars or pipes?

1 ❑ 1

2 ❑ 2

3 ❑ 3

4 ❑ 4 or more

**123.** **In the last month,** *on average*, how many hours per day were you *exposed* to cigarette, cigar or pipe smoke because of smoking by others?

1 ❑ 0-1 hour

2 ❑ 1-2 hours

4 ❑ 3-4 hours

5 ❑ More than 4 hours

3 ❑ 2-3 hours

**124.** Have you ever smoked electronic cigarettes/e-cigarettes or other electronic nicotine device (e-hookah, e-cigars, etc.)?

0 ❑ No (**SKIP TO #127**)

1 ❑ Yes

**125.** **In the last month,** have you smoked electronic cigarettes/e-cigarettes or other electronic nicotine device (e-hookah, e-cigars, etc.)?

0 ❑ No **(SKIP TO #127)**

1 ❑ Yes

**126.** Have you smoked electronic cigarettes/e-cigarettes or other electronic nicotine device (e-hookah, e-cigars, etc.) in the last 5 days?

0 ❑ No:

**A.** If you are NOT currently smoking, when did you stop smoking? [**MARK ONE**]

1 ❑ Less than 2 weeks ago 3 ❑ More than 4 weeks ago

2 ❑ 2 to 4 weeks ago 9 ❑ Don't remember

1. If you are NOT currently smoking, how often did you smoke electronic cigarettes/e-cigarettes or other electronic nicotine devices (e-hookah, e-cigars, etc.)?

1❑ Every day 4❑ About once a month

2❑ Every few days 5❑ Every few months

3❑ Once a week

1 ❑ Yes:

**A.** How often do you smoke electronic cigarettes/e-cigarettes or other electronic nicotine devices (e-hookah, e-cigars, etc.)?

1❑ Every day

2❑ Every few days

3❑ Once a week

4❑ About once a month

5❑ Every few months

**HOME CHARACTERISTICS**

**127**. **Which best describes the home in which you currently live most of the time**? *Mark one.*

1 ❑ A house (not connected to other homes)

2 ❑ A building with 2-4 attached apartments, town houses, condos, a duplex or a triplex

3 ❑ A building with 5-10 attached apartments, town houses, condos, etc.

4 ❑ A building with more than 10 attached apartments, town houses, condos, etc.

5 ❑ Mobile home or trailer

6 ❑ Other, please be specific: ___________________________________________________

**128**. **About when was this structure originally built**? (When it was first constructed, not when it may have been remodeled, added to, or converted.) *Mark one.*

1❑ 2000s or later 4❑ 1940s-1950s

2❑ 1980s-1990s 5❑ Before 1940

3❑ 1960s-1970s

**129.** **In the last month,** which of the following pets do you keep inside your home? **(MARK ALL THAT APPLY)**

1 ❑ No Pets

2 ❑ Dog(s)

3 ❑ Cat(s)

4 ❑ Other pets (Explain: ____________)

**130.** **In the last month,** have you had any of the following pests in your home? **(MARK ALL THAT APPLY)**

1 ❑ Rats

2 ❑ Mice

3 ❑ Cockroaches

4 ❑ Other pests (Specify: ________________)

5 ❑ Don’t know

6 ❑ No pests

**131**. Is there a cooking stove, range, or oven in your home that uses GAS**?**

0 ❑ No

1 ❑ Yes:

**A.** How often is the gas stove, range or oven used while you are at home? *Mark one.*

1 ❑ Never **(SKIP to 132)**

2 ❑ Less than once a week

3 ❑ 1-3 times per week

4 ❑ 4-7 times per week

5 ❑ 8-14 times per week

6 ❑ More than 14 times per week

**B.** About how long is the gas stove, range or oven used on an average day while you are at home?

1 ❑ Less than 15 minutes

2 ❑ 15 minutes to less than 30 minutes

3 ❑ 30 minutes to less than 1 hour

4 ❑ 1 hour or more

**132.** **In the last month,** on average, how many times a week do you cook (using the stove/range/oven, not microwave)?

_1_❑ Never

_2_❑ 1 – 3 times a week

_3_❑ 4 – 5 times a week

_4_❑ Every day of the week

**133.** Does your home have heating?

0 ❑ No **(SKIP to #135)**

1 ❑ Yes:

**A.** What is the main fuel used to heat it? *Mark one.*

1 ❑ Gas (you may be able to see a blue flame or pilot light in the unit)

2 ❑ Electricity (you may be able to see a red-hot glowing wire in the unit)

3 ❑ Bottles, tank or L.P./liquefied petroleum gas (a tank outside that a truck may fill with gas)

4 ❑ Firewood

5 ❑ Other, please be specific: ________________________

9 ❑ Don’t know how it is heated

**134.** What is the one main heating system in your home? *Mark one.*

1 ❑ Forced air

2 ❑ Built-in electric unit

3 ❑ Wall heater

4 ❑ Floor heater

5 ❑ Portable space heater… **Which type?**

1 ❑ Gas

2 ❑ Electric

3 ❑ Don’t Know

6 ❑ Other, please be specific: ______________________________

9 ❑ Don’t know how it is heated

**135.** Do you use air conditioning in your home?

0 ❑ No **(SKIP to #138)**

1 ❑ Yes: **A.** What is the main kind of air conditioning that is used? *Mark one.*

1 ❑ Wall or window unit (box that sticks out of window or wall)

**a.** How many wall/window units do you have in your home?

_1_ ❑ One

_2_ ❑ Two

_3_ ❑ Three

_4_ ❑ Four or more

_5_ ❑ Don’t know

2 ❑ Central (vents in the room)

3 ❑ Swamp/desert/evaporative cooler

9 ❑ Don’t know what kind it is

**136**. **During the last month,** about how often did you use air conditioning when you were at home?

1 ❑ Never

2 ❑ Less than 5 days

3 ❑ 5-15 days

4 ❑ 16-30 days

9 ❑ Don’t know

**137.** On any given day, how much of the time did you use the air conditioner at home?

_1_ ❑ None of the time

_2_ ❑ A couple of hours a day

_3_ ❑ Half of the time

_4_ ❑ Most of the time

_5_ ❑ All of the time

_9_ ❑ Don’t know

**138.** During the last month, did you use a window fan or other fan that you placed in the window or an attic fan to cool your home?

0 ❑ No

1 ❑ Yes

**139.** Has there been water damage OR flooding in your home **in the last month**?

0 ❑ No

1 ❑ Yes: **A.** Did it flood carpeted areas?

0 ❑ No

1 ❑ Yes

9 ❑ Don’t know

**140.** Has there ever been mold or mildew on the walls, ceilings, or floors inside your home **in the last month**?

0 ❑ No

1 ❑ Yes: **A.** Which rooms were affected? *Mark all that apply.*

1 ❑ The room where you sleep

2 ❑ Bathroom(s)

3 ❑ Basement

4 ❑ Other

9 ❑ Don’t know

**141.** Is a humidifier or vaporizer ever used in your home? (Include humidifier built into heating system.)

0 ❑ No

1 ❑ Yes: **A.** What type is it? *Mark all that apply.*

1 ❑Built into heating system

2 ❑Free standing or portable unit

**B.** Have you used this unit for treating a respiratory illness?

0 ❑No 1 ❑Yes

**C.**  Does the humidifier or vaporizer heat the air?

0 ❑No 1 ❑Yes 9 ❑Don’t know

9 ❑ Don’t know

**142**. Is there carpeting in your home?

0 ❑ No

1 ❑ Yes:

**A.** In what rooms? *Mark all that apply.*

1 ❑ Whole house (excluding kitchen and bath)

2 ❑ Room where you sleep

3 ❑ Other bedroom(s)

4 ❑ Other room(s)

**143.** Thinking back to a typical *weekday* in this past **week**, approximately how many hours (out of 24 hours in total) did you spend…

**A.** Outdoors: ________________

**B.** Indoors at home (Include nighttime/sleeping):____________________

**144.** On average, how much of the time were the windows open in your home this past **week**?

_1_ ❑ None of the time

_2_ ❑ A couple of hours a day

_3_ ❑ Half of the time

_4_ ❑ Most of the time

_5_ ❑ All of the time

_9_ ❑ Don’t know

**SLEEP QUESTIONS**

**Next, we are going to ask you about your sleeping patterns and habits during the past month (30 days). Think about over the past month (30 days).**

**145. In the past month,** how many hours of sleep did you usually get on a typical weeknight (Sunday - Thursday)?

_1_ ❑ Less than 4 hours per night _5_ ❑ 8 hours per night

_2_ ❑ 5 hours per night _6_ ❑ 9 hours per night

_3_ ❑ 6 hours per night _7_ ❑ More than 10 hours per night

_4_ ❑ 7 hours per night

**146.** **In the past month,** how many hours of sleep did you usually get on a typical weekend night (Friday or Saturday)?

_1_ ❑ Less than 4 hours per night _5_ ❑ 8 hours per night

_2_ ❑ 5 hours per night _6_ ❑ 9 hours per night

_3_ ❑ 6 hours per night _7_ ❑ More than 10 hours per night

_4_ ❑ 7 hours per night

**Questions 147-150 Jenkins Sleep Questionnaire**

Jenkins CD, Stanton B-A, Niemcrym SJ, Rose RM. A scale for the estimation of sleep problems in clinical research. J Clin Epidemiol 1988;41:313-21.

**151.** **During the LAST YEAR, how often, on average, have you snored or been told you snore DURING SLEEP? (MARK ONE)**

_1_ ❑ Never _4_ ❑ Frequently (3 to 4 times per week)

_2_ ❑ Rarely (Less than once a week) _5_ ❑ Always/Almost always (5 to 7 times per week)

_3_ ❑ Sometimes (1 to 2 times per week) _9_ ❑ Not sure

**PARENTS’ PLACE OF BIRTH**

**152.** **Was your father born in the USA?**

0 ❑ No…… A. What country was your father born in? ___________________________

1 ❑ Yes……B. What city was your father born in? ______________________________

C. What state was your father born in? _____________________________

**153.** **Was your mother born in the USA?**

0 ❑ No…… A. What country was your mother born in? ___________________________

1 ❑ Yes……B. What city was your mother born in? ______________________________

C. What state was your mother born in? _____________________________

**BIRTH PLAN**

**154.** **Where do you plan on delivering your baby?**

_1_ ❑ LAC+USC

_2_ ❑ White Memorial Medical Center

_3_ ❑ California Hospital Medical Center

_4_ ❑ Good Samaritan Hospital

_5_ ❑ Home

_6_ ❑ Other…Please specify: ________________________

_9_ ❑ Not sure

**155.** **Will you be having a scheduled delivery/planned Cesarean birth?**

0 ❑ No

1 ❑ Yes:

A. When is the delivery/Cesarean section scheduled for?

Date: ______/_____/_______

Time: _____________
