# Supplementary material for: Study Design, Protocol and Profile of the Maternal And Developmental Risks from Environmental and Social Stressors (MADRES) Pregnancy Cohort: a Prospective Cohort Study in Predominantly Low-Income Hispanic Women in Urban Los Angeles
Source: BMC Pregnancy Childbirth. 2019 May 30;19:189. doi: 10.1186/s12884-019-2330-7 (PMC6543670; doi:10.1186/s12884-019-2330-7)
Supplement: Supplementary file 15 — Occupational History Form. Occupational history questionnaire mailed to participants to complete and bring to the third trimester visit. (DOCX 37 kb) [file 12884_2019_2330_MOESM15_ESM.docx]

MADRES STUDY ID#__________

**OCCUPATIONAL HISTORY FORM**

**Please answer the following questions for each job you have worked at, so that we can learn more about where you have worked for the last two years (since ____/____/______). Start with the most recent job and work your way back in time.**

| **Job #1** | | | |
| --- | --- | --- | --- |
| 1. **What is/was the name and address of the company where you work/worked? (If you don’t remember the address, please provide the name of the nearest cross streets.)**   company name    street address  town/city and state zip code (if known) | | | |
| 1. **When did you start and stop (if applicable) working at this job?**   START: __________ / _________ END: ________ / _______ **□** Still Employed Here  Month Year Month Year | | | |
| 1. **What percentage of your work day do/did you spend working at the above location?**   □ All of the time □ Most of the time □Some of the time □None of the time | | | |
| 1. **What was/is the job title you held/hold at this company?** | | 1. **What shift(s) do you work? (Check all that apply)**   □ Morning/Day □ Swing/Evening □ Graveyard/Night | |
| 1. **Was/is this a full-time or part-time job?**   □ Full-Time(year-round) □ Part-Time(year-round)  □ Full-Time(seasonal) □ Part-Time(seasonal) | | 1. **Does/Did this job require you to work outdoors?**   □ No □ Yes…How often? □All of the time  □Most of the time  □Some of the time  □None of the time | |
| 1. **What type of business was/is it?** | | | |
| □ Automobile  □ Construction  □ Education  □ Farming | □ Health Care  □ Hotel  □ Janitorial  □ Manufacturer | | □ Office work  □ Restaurant  □ Retail  □ Other, please describe: _________________ |
| 1. **What were/are your main activities or duties for this job?** | | | |
| 1. **What kinds of chemicals or materials did/do you handle in this job?**   □ None | | | |
| 1. **What kind of tools and equipment did/do you use?**   □ None | | | |

MADRES STUDY

**OCCUPATIONAL HISTORY FORM-Page 2**

| **Job #2** | | | |
| --- | --- | --- | --- |
| 1. **What was/is the name and address of the company where you worked/work? (If you don’t remember the address, please provide the name of the nearest cross streets.)**   company name    street address  town/city and state zip code (if known) | | | |
| 1. **In what year did you start and stop (if applicable) working at this job?**   START: __________ / _________ END: ________ / _______ **□** Still Employed Here  Month Year Month Year | | | |
| 1. **What percentage of your work day do/did you spend working at the above location?**   □ All of the time □ Most of the time □Some of the time □None of the time | | | |
| 1. **What was/is the job title you held/hold at this company?** | | 1. **What shift(s) do you work? (Check all that apply)**   □ Morning/Day □ Swing/Evening □ Graveyard/Night | |
| 1. **Was/is this a full-time or part-time job?**   □ Full-Time(year-round) □ Part-Time(year-round)  □ Full-Time(seasonal) □ Part-Time(seasonal) | | 1. **Does/Did this job require you to work outdoors?**   □ No □ Yes…How often? □All of the time  □Most of the time  □Some of the time  □None of the time | |
| 1. **What type of business was/is it?** | | | |
| □ Automobile  □ Construction  □ Education  □ Farming | □ Health Care  □ Hotel  □ Janitorial  □ Manufacturer | | □ Office work  □ Restaurant  □ Retail  □ Other, please describe: _________________ |
| 1. **What were/are your main activities or duties for this job?** | | | |
| 1. **What kinds of chemicals or materials did/do you handle in this job?**   □ None | | | |
| 1. **What kind of tools and equipment did/do you use?**   □ None | | | |
