# Supplementary material for: Study Design, Protocol and Profile of the Maternal And Developmental Risks from Environmental and Social Stressors (MADRES) Pregnancy Cohort: a Prospective Cohort Study in Predominantly Low-Income Hispanic Women in Urban Los Angeles
Source: BMC Pregnancy Childbirth. 2019 May 30;19:189. doi: 10.1186/s12884-019-2330-7 (PMC6543670; doi:10.1186/s12884-019-2330-7)
Supplement: Supplementary file 16 — Occupational History Form_Spanish. Spanish occupational history questionnaire mailed to participants to complete and bring to the third trimester visit. (DOCX 38 kb) [file 12884_2019_2330_MOESM16_ESM.docx]

ESTUDIO MADRES ID#__________

**HISTORIAL OCCUPACIONAL**

**Por favor, conteste las siguientes preguntas para cada trabajo en que usted ha trabajado, para poder aprender más acerca de donde usted ha trabajado durante los últimos dos años (desde ____ / ____ / ______). Comience con el trabajo más reciente.**

| **Trabajo #1** | | | |
| --- | --- | --- | --- |
| 1. **¿Cuál es el nombre y dirección de la compañía donde trabaja/trabajo? (Si no se acuerda la dirección, por favor escriba el nombre de las calles de cruce más cercanas.)**   nombre de la compañía    calle y número  pueblo/ciudad y estado código postal (si lo sabe) | | | |
| 1. **¿Cuándo comenzó y paró (si aplicable) de trabajar en este trabajo?**   COMENZO: __________ / _________ PARÓ: ________ / _______ **□** Todavía empleada aquí  Mes Año Mes Año | | | |
| 1. **¿Qué porcentaje de su día de trabajo pasa/paso trabajando en la ubicación de arriba?**   □ Todo el tiempo □ Mayor parte del tiempo □Parte del tiempo □Ninguna parte del tiempo | | | |
| 1. **¿Cuál es/era su título de trabajo que tiene/tuvo en esta compañía?** | | 1. **¿Qué turno(s) trabaja? (Marque todo que aplique)**   □ Mañana/Día □ Tarde □ Noche | |
| 1. **¿Era/es este trabajo a tiempo complete o tiempo parcial?**   □ Tiempo completo (todo el año)  □ Tiempo completo (estacional/temporal)  □ Tiempo parcial (todo el año)  □ Tiempo parcial (estacional/temporal) | | 1. **¿Este trabajo requiere/requirió que trabajara afuera al aire libre?**   □ No □ Si…¿Que tan seguido? □Todo el tiempo  □Mayor parte del tiempo  □Parte del tiempo  □Ninguna parte del tiempo | |
| 1. **¿Qué tipo de negocio era/es?** | | | |
| □ Automovilista  □ Construcción  □ Educación  □ Agricultura | □ Cuidado de Salud  □ Hotel  □ Limpieza  □ Fabricante | | □ Trabajo de Oficina  □ Restaurante  □ Venta al por menor  □ Otro, por favor describa: _________________ |
| 1. **¿Cuáles son/eran sus actividades o funciones principales de este trabajo?** | | | |
| 1. **¿Qué tipo de químicos o materiales maneja/manejo en este trabajo?**   □ None | | | |
| 1. **¿Qué tipo de herramientas y equipos utiliza/utilizo?**   □ None | | | |

ESTUDIO MADRES

**HISTORIAL OCCUPACIONAL-Pagina 2**

| **Trabajo #2** | | | |
| --- | --- | --- | --- |
| 1. **¿Cuál es el nombre y dirección de la compañía donde trabaja/trabajo? (Si no se acuerda la dirección, por favor escriba el nombre de las calles de cruce más cercanas.)**   nombre de la compañía    calle y número  pueblo/ciudad y estado código postal (si lo sabe) | | | |
| 1. **¿Cuándo comenzó y paró (si aplicable) de trabajar en este trabajo?**   COMENZO: __________ / _________ PARÓ: ________ / _______ **□** Todavía empleada aquí  Mes Año Mes Año | | | |
| 1. **¿Qué porcentaje de su día de trabajo pasa/paso trabajando en la ubicación de arriba?**   □ Todo el tiempo □ Mayor parte del tiempo □Parte del tiempo □Ninguna parte del tiempo | | | |
| 1. **¿Cuál es/era su título de trabajo que tiene/tuvo en esta compañía?** | | 1. **¿Qué turno(s) trabaja? (Marque todo que aplique)**   □ Mañana/Día □ Tarde □ Noche | |
| 1. **¿Era/es este trabajo a tiempo complete o tiempo parcial?**   □ Tiempo completo (todo el año)  □ Tiempo completo (estacional/temporal)  □ Tiempo parcial (todo el año)  □ Tiempo parcial (estacional/temporal) | | 1. **¿Este trabajo requiere/requirió que trabajara afuera al aire libre?**   □ No □ Si…¿Que tan seguido? □Todo el tiempo  □Mayor parte del tiempo  □Parte del tiempo  □Ninguna parte del tiempo | |
| 1. **¿Qué tipo de negocio era/es?** | | | |
| □ Automovilista  □ Construcción  □ Educación  □ Agricultura | □ Cuidado de Salud  □ Hotel  □ Limpieza  □ Fabricante | | □ Trabajo de Oficina  □ Restaurante  □ Venta al por menor  □ Otro, por favor describa: _________________ |
| 1. **¿Cuáles son/eran sus actividades o funciones principales de este trabajo?** | | | |
| 1. **¿Qué tipo de químicos o materiales maneja/manejo en este trabajo?**   □ None | | | |
| 1. **¿Qué tipo de herramientas y equipos utiliza/utilizo?**   □ None | | | |
