# Supplementary material for: Study Design, Protocol and Profile of the Maternal And Developmental Risks from Environmental and Social Stressors (MADRES) Pregnancy Cohort: a Prospective Cohort Study in Predominantly Low-Income Hispanic Women in Urban Los Angeles
Source: BMC Pregnancy Childbirth. 2019 May 30;19:189. doi: 10.1186/s12884-019-2330-7 (PMC6543670; doi:10.1186/s12884-019-2330-7)
Supplement: Supplementary file 17 — Residential History Form. Residential history questionnaire mailed to participants to complete and bring to the third trimester visit. (DOC 72 kb) [file 12884_2019_2330_MOESM17_ESM.doc]

MADRES STUDY ID#__________

**RESIDENTIAL HISTORY FORM**

Please answer the following questions, so that we can learn more about where you have lived for the last two years (since ____/____/______). Answer as many questions as you can.

In the questions below, ‘residence’ means the home, apartment, dormitory or other building that you live/lived in. If you “lived” in two different places at the same time (for example, living parts of the week with different parents in different locations), please complete a section for each of the residences and tell us how much time you spend at each residence.

Start with the most recent residence (where you are living right now) and work your way back in time, as far as you can go.

|  | **ADDRESS** (If you don’t remember the address, please provide the name of the nearest cross streets.) | **WHEN DID YOU LIVE HERE?** |
| --- | --- | --- |
| 1  2  3  4 | **R**  **E**  **S**  **I**  **D**  **E**  **N**  **C**  **E**    **#1**  street address  town/city and state  zip code (if known) | MOVED IN: __________ / _________  Month Year  MOVED OUT:________ / _______  Month Year |
|  | **R**  **E**  **S**  **I**  **D**  **E**  **N**  **C**  **E**    **#2**  street address  town/city and state  zip code (if known)  **R**  **E**  **S**  **I**  **D**  **E**  **N**  **C**  **E**    **#3** | MOVED IN: __________ / _________  Month Year  MOVED OUT:________ / _______  Month Year |
|  | street address  town/city and state  zip code (if known)  **3** | MOVED IN: __________ / _________  Month Year  MOVED OUT:________ / _______  Month Year |
|  | **R**  **E**  **S**  **I**  **D**  **E**  **N**  **C**  **E**    **#4**  street address  town/city and state  zip code (if known) | MOVED IN: __________ / _________  Month Year  MOVED OUT:________ / _______  Month Year |

MADRES STUDY ID#________

**RESIDENTIAL HISTORY FORM-PAGE 2**

| **ADDRESS** (If you don’t remember the address, please provide the name of the nearest cross streets. **R**  **E**  **S**  **I**  **D**  **E**  **N**  **C**  **E**    **#5** | | **WHEN DID YOU LIVE HERE?** | |  |
| --- | --- | --- | --- | --- |
| 5  6  7  8 | street address  town/city and state  zip code (if known)  **5** | | MOVED IN: __________ / _________  Month Year  MOVED OUT:________ / _______  Month Year | |
|  | **R**  **E**  **S**  **I**  **D**  **E**  **N**  **C**  **E**    **#6**  street address  town/city and state  zip code (if known) | | MOVED IN: __________ / _________  Month Year  MOVED OUT:________ / _______  Month Year | |
|  | **R**  **E**  **S**  **I**  **D**  **E**  **N**  **C**  **E**    **#7**  street address  town/city and state  zip code (if known) | | MOVED IN: __________ / _________  Month Year  MOVED OUT:________ / _______  Month Year | |
|  | **R**  **E**  **S**  **I**  **D**  **E**  **N**  **C**  **E**    **#8**  street address  town/city and state  zip code (if known) | | MOVED IN: __________ / _________  Month Year  MOVED OUT:________ / _______  Month Year | |
