# Supplementary material for: Study Design, Protocol and Profile of the Maternal And Developmental Risks from Environmental and Social Stressors (MADRES) Pregnancy Cohort: a Prospective Cohort Study in Predominantly Low-Income Hispanic Women in Urban Los Angeles
Source: BMC Pregnancy Childbirth. 2019 May 30;19:189. doi: 10.1186/s12884-019-2330-7 (PMC6543670; doi:10.1186/s12884-019-2330-7)
Supplement: Supplementary file 18 — Residential History Form_Spanish. Spanish residential history questionnaire mailed to participants to complete and bring to the third trimester visit. (DOC 73 kb) [file 12884_2019_2330_MOESM18_ESM.doc]

ESTUDIO MADRES ID#__________

**HISTORIAL RESIDENCIAL**

Por favor, conteste las siguientes preguntas, para poder aprender más acerca de donde ha vivido durante los últimos dos años (desde ____ / ____ / ______). Responda a tantas preguntas como sea posible.

En las preguntas de abajo, ‘residencia’ significa la casa, apartamento, residencia u otro edificio que vive/vivía. Si "vivió" en dos lugares diferentes al mismo tiempo (por ejemplo, parte de la semana con diferentes padres en diferentes lugares), por favor complete una sección para cada una de las residencias y díganos la cantidad de tiempo que pasa en cada residencia.

Comience con su residencia más reciente (donde vive ahora).

|  | **DIRECCIÓN** (Si no recuerda la dirección, proporcione el nombre de las calles de cruce más cercanas.) | **¿CUÁNDO VIVIO AQUÍ?** |
| --- | --- | --- |
| 1  2  3  4 | **RESIDENCIA**  **#1**  calle y número  pueblo/ciudad y estado  código postal (si lo sabe) | DESDE: __________ / _________  Mes Año  HASTA:________ / _______  Mes Año |
|  | **R**  **E**  **SIDENCIA**  **#2**  calle y número  pueblo/ciudad y estado  código postal (si lo sabe)  **R**  **E**  **S**  **I**  **D**  **ENCIA**  **#3** | DESDE: __________ / _________  Mes Año  HASTA:________ / _______  Mes Año |
|  | calle y número  pueblo/ciudad y estado  código postal (si lo sabe))  **3** | DESDE: __________ / _________  Mes Año  HASTA:________ / _______  Mes Año |
|  | **R**  **E**  **S**  **I**  **D**  **E**  **N**  **C**  **IA**  **#4**  calle y número  pueblo/ciudad y estado  código postal (si lo sabe) | DESDE: __________ / _________  Mes Año  HASTA:________ / _______  Mes Año |

ESTUDIO MADRES ID#________

**HISTORIAL RESIDENCIAL-PAGINA 2**

| **DIRECCIÓN** (Si no recuerda la dirección, proporcione el nombre de las calles de cruce más cercanas.) **R**  **E**  **S**  **I**  **D**  **E**  **N**  **C**  **IA**  **#5** | | **¿CUÁNDO VIVIO AQUÍ?** | |  |
| --- | --- | --- | --- | --- |
| 5  6  7  8 | calle y número  pueblo/ciudad y estado  código postal (si lo sabe)  **5** | | DESDE: __________ / _________  Mes Año  HASTA:________ / _______  Mes Año | |
|  | **R**  **E**  **S**  **I**  **D**  **E**  **N**  **C**  **IA**  **#6**  calle y número  pueblo/ciudad y estado  código postal (si lo sabe) | | DESDE: __________ / _________  Mes Año  HASTA:________ / _______  Mes Año | |
|  | **R**  **E**  **S**  **I**  **D**  **E**  **N**  **C**  **IA**  **#7**  calle y número  pueblo/ciudad y estado  código postal (si lo sabe) | | DESDE: __________ / _________  Mes Año  HASTA:________ / _______  Mes Año | |
|  | **R**  **E**  **S**  **I**  **D**  **E**  **N**  **C**  **IA**  **#8**  calle y número  pueblo/ciudad y estado  código postal (si lo sabe) | | DESDE: __________ / _________  Mes Año  HASTA:________ / _______  Mes Año | |
