# Supplementary material for: Study Design, Protocol and Profile of the Maternal And Developmental Risks from Environmental and Social Stressors (MADRES) Pregnancy Cohort: a Prospective Cohort Study in Predominantly Low-Income Hispanic Women in Urban Los Angeles
Source: BMC Pregnancy Childbirth. 2019 May 30;19:189. doi: 10.1186/s12884-019-2330-7 (PMC6543670; doi:10.1186/s12884-019-2330-7)
Supplement: Supplementary file 19 — 7-14 Day Questionnaire. Questionnaire administered 7-14 days after child participant is born. (DOCX 42 kb) [file 12884_2019_2330_MOESM19_ESM.docx]

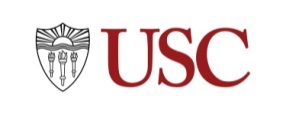
**MADRES Study: 7-14 Day Questionnaire**

**Today’s Date :** _____________________ **Interviewer Name:** ____________________

**Instructions:**  Please see 7-14 Day Questionnaire Phone Script.

**CONTACT INFORMATION**

**1. Name:** _________________ _______________ ____________________ ____________________

First Middle Last 1 Last 2

**2. Your Date of Birth:** **_______/_______/_______**

Month Day Year

**3. What is your cell phone number?** ____________________________

□₀ Don’t have a cell phone **(Skip to question #5)**

**4.** **Is this a prepaid cell phone or a permanent phone number?**

□₀ prepaid

□₁ permanent number

**5.** **What is your HOME address (the address at which you spend the most time)?**

Address: ______________________________________________________________________

City: ___________________________State: ________________Zip: _____________________

**6.** **What is the phone number for the HOME listed in Question 5?** ____________________________

□₀ Don’t have a home phone

**7. Do you live at more than one home?**

□₀No… *Skip to 9* □₁ Yes... *Complete 8A-C*

**8A.** **What is your second HOME address?**

Address: ______________________________________________________________________

City: ___________________________State: ________________Zip: _____________________

**8B. What is the phone number for the HOME listed in Question 8A?** _______________________

□₀ Don’t have a home phone

**8C. How much time do you spend at the address listed in 8A?**

₁ 1%-25% of the time

_2_ 26%-50% of the time

**9.** **What is your baby’s HOME address?**

₁ Same as the HOME address listed in Question 5 (*Skip to Question 12*)

_2_ Same as the Second Home address listed in Question 8 (*Skip to Question 12*)

_3_ Same as both addresses listed in Questions 5 & 8 (*Skip to Question 12*)

_4_ Other (Continue to 9A)

**9A.** **What is your baby’s home address?**

Address: ______________________________________________________________________

City: ___________________________State: ________________Zip: _____________________

**10. Does your baby live at more than one home?**

□₀No… *Skip to 12* □₁ Yes... *Complete 11A-B*

**11A.** **What is your baby’s second HOME address?**

Address: ______________________________________________________________________

City: ___________________________State: ________________Zip: _____________________

**11B. How much time does your baby spend at the address listed in 9A?**

₁ 1%-25% of the time

_2_ 26%-50% of the time

**Baby’s Birth**

**12**. **Baby’s Name:** _________________ ______________ ____________________ ____________________

First Middle Last 1 Last 2

### **13**. **Baby’s DOB:** **_______/_______/_______**

Month Day Year

**14**. **Baby’s Gender:** □₁ Female □₂ Male

**15**. **What was your baby’s weight at birth?** Pounds ____________ AND Ounces____________

**16**. **What was your baby’s length at birth?** ____________ INCHES

**17. Where was your baby born?**

1 ❑ LAC+USC County Hospital

2 ❑ California Hospital Medical Center

3 ❑ Other Hospital: __________________________________________

4 ❑ Other (please explain): __________________________________________

**18. How was your baby delivered?**

1 ❑ Vaginally and not induced

2 ❑ Vaginally and induced

3 ❑ A planned cesarean

4 ❑ An unplanned cesarean

**19. Which of the following medications did you have during labor or delivery?** (Mark all that apply)

1 ❑ General anesthesia (you were put to sleep)

2 ❑ A spinal or epidural

3 ❑ Demerol or Stadol

4 ❑ Nitrous oxide (gas breathed through a gas mask or mouthpiece while remaining conscious)

5 ❑ Pudendal block or other local blocks (injection into the vagina or cervix before the birth)

6 ❑ Other pain medication or don’t know which pain medication

7 ❑ No pain medication

**20. How many nights were you in the hospital or birth center after your baby was born?**

0 ❑ None

1 ❑ 1 night

2 ❑ 2 nights

3 ❑ 3 nights

4 ❑ 4 to 7 nights

5 ❑ More than 7 nights

**21. Did your baby have to stay in an intensive care unit?**

0 ❑ No (*Skip to Question 22*)

1 ❑ Yes, 3 days or less

2 ❑ Yes, more than 3 days

**21A. Why did your baby have to stay in an intensive care unit? (select all that apply)**

1 ❑ Premature birth

2 ❑ Complications during delivery

3 ❑ Low birth weight

4 ❑ Low Apgar score

5 ❑ Birth defects

6 ❑ Anemia

7 ❑ Jaundice

8 ❑ Respiratory problems

9 ❑ Heart problems

A ❑ Surgery

B ❑ Infection

C ❑ Other (Please explain):_________________________________________________________

D ❑ Do not know

**Infant Feeding**

**Questions 22-26 Infant Feeding Practices**

Fein SB, Labiner-Wolfe J, Shealy KR, Li R, Chen J, Grummer-Strawn LM: **Infant Feeding Practices Study II: study methods**. *Pediatrics* 2008, **122 Suppl 2**:S28-35.

**27. Does your baby have any special needs or medical problems?**

0 ❑ No

1 ❑ Yes (*Complete 27A*)

**27A. Please explain briefly: ______________________________________________________________________________________________________________________________________________________________________________________________________________________________________________________________________________**

**THANK YOU FOR COMPLETING THIS QUESTIONNAIRE! CONGRATULATIONS ON YOUR NEW BABY.**
