# Supplementary material for: Study Design, Protocol and Profile of the Maternal And Developmental Risks from Environmental and Social Stressors (MADRES) Pregnancy Cohort: a Prospective Cohort Study in Predominantly Low-Income Hispanic Women in Urban Los Angeles
Source: BMC Pregnancy Childbirth. 2019 May 30;19:189. doi: 10.1186/s12884-019-2330-7 (PMC6543670; doi:10.1186/s12884-019-2330-7)
Supplement: Supplementary file 20 — 7-14 Day Questionnaire_Spanish. Spanish questionnaire administered 7-14 days after child participant is born. (DOCX 43 kb) [file 12884_2019_2330_MOESM20_ESM.docx]

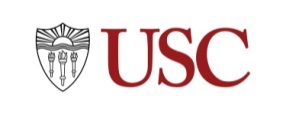
**MADRES Study: 7-14 Day Questionnaire**

**Today’s Date:** _____________________ **Interviewer Name:** ____________________

**Instructions:**  Please see 7-14 Day Questionnaire Phone Script.

**INFORMACIÓN PERSONAL**

**1. Nombre:** ________________ _______________ ____________________ ____________________

Nombre 2do Nombre Apellido 1 Apellido 2

**2. Fecha de nacimiento:** _**______/_______/_______**

Mes Día Año

**3. ¿Cuál es su número de celular?** ____________________________

□₀ No tengo teléfono celular **(Skip to question #5)**

**4.** **¿Es un celular pre pagado o es un número fijo de celular?**

□₀ Pre pagado

□₁ Número fijo

**5.** **¿Cuál es su dirección? (la dirección donde usted pasa la mayoría del tiempo):**

Dirección: ________________________________________________________________________

Ciudad: _____________________Estado: ________________Código Postal: ___________________

**6.** **¿Cuál es el número de teléfono para el domicilio dado en la Pregunta 5?**______________________

□₀ No tengo teléfono de casa

**7. ¿Vive en más de una casa?**

□₀No… *Skip to 9* □₁ Sí... *Complete 8A-C*

**8A.** **¿Cuál es la dirección de su segundo domicilio?**

Dirección: _________________________________________________________________________

Ciudad: ______________________Estado: ________________ Código Postal: __________________

**8B. ¿Cuál es el número de teléfono para el domicilio dado en la Pregunta 8A?** _______________________

□₀ No tengo teléfono de casa

**8C. ¿Cuánto tiempo pasa usted en la dirección dada en la Pregunta 8A?**

 1%-25% del tiempo

 26%-50% del tiempo

**9.** **¿Cuál es la dirección del domicilio de su bebe?**

₁ Igual que la dirección de CASA mencionada en la pregunta 5 (*Skip to Question 12*)

_2_ Igual que la segunda dirección de CASA mencionada en la pregunta 8 (*Skip to Question 12*)

_3_ Igual que las dos direcciones mencionadas en las Preguntas 5 y 8 (*Skip to Question 12*)

_4_ Otra (*Continue to* 9A)

**9A. ¿Cuál es la dirección del domicilio de su bebé?**

Dirección: _________________________________________________________________________

Ciudad: ______________________Estado: ________________ Código Postal: __________________

**10. ¿Vive su bebé en más de un domicilio?**

□₀No… *Skip to 12* □₁ Sí... *Complete 11A-B*

**11A.** **. ¿Cuál es la segunda dirección de domicilio de su bebé?**

Dirección: _________________________________________________________________________

Ciudad: ______________________Estado: ________________ Código Postal: __________________

**11B. ¿Cuánto tiempo pasa su bebé en la dirección dada en la Pregunta 9A?**

 1%-25% del tiempo

 26%-50% del tiempo

**NACIMIENTO DEL BEBÉ**

**12**. **Nombre del Bebé:** ________________ _______________ _________________ _________________

Nombre 2do Nombre Apellido 1 Apellido 2

**13**. **Fecha de nacimiento del Bebé:** _**______/_______/_______**

Mes Día Año

**14**. **Género del Bebé:** □₁ Femenino □₂ Masculino

**15**. **¿Cuál fue el peso del bebé al nacer?** Libras ____________ Y Onzas____________

**16**. **¿Cuánto midió su bebé al nacer?**____________ Pulgadas

**17. ¿En qué hospital nació su bebé?**

1 ❑ Hospital del Condado LAC+USC

2 ❑ Centro Médico del Hospital California

3 ❑ Otro Hospital: __________________________________________

4 ❑ Otro (por favor explique): __________________________________________

**18. ¿De qué modo dio usted a luz a su bebé?**

1 ❑ Por vía vaginal y sin inducción

2 ❑ Por vía vaginal y con inducción

3 ❑ Una cesárea planificada

4 ❑ Una cesárea no planificada

**19. ¿Cuál de los siguientes medicamentos tuvo usted durante el parto?** (Marque todos lo que correspondan)

1 ❑ Bajo anestesia general (la pusieron a dormir)

2 ❑ Un espinal o epidural

3 ❑ Demerol o Stadol

4 ❑ Óxido nitroso (gas respirado a través de una máscara de gas o por una boquilla mientras permaneció consciente)

5 ❑ Bloqueo pudendo u otros bloqueos locales (inyección en la vagina o el cuello uterino antes del nacimiento

6 ❑ Otros medicamentos para el dolor o no sabe qué medicamentos para el dolor

7 ❑ Ningún medicamento para el dolor

**20. ¿Cuántas noches estuvo en el hospital o centro de nacimiento después del nacimiento de su bebé?**

0 ❑ Ninguna

1 ❑ 1 noche

2 ❑ 2 noches

3 ❑ 3 noches

4 ❑ 4 a 7 noches

5 ❑ Más de 7 noches

**21. ¿Su bebé tuvo que permanecer en una unidad de cuidados intensivos?**

0 ❑ No (*Skip to Question 22*)

1 ❑ Sí, 3 días o menos

2 ❑ Sí, más de 3 días

**21A. ¿Por qué tuvo que permanecer su bebé en una unidad de cuidados intensivos?** (Marque todos lo que correspondan)

1 ❑ Nacimiento prematuro

2 ❑ Complicaciones durante el parto

3 ❑ Bajo peso al nacer

4 ❑ Índice de Apgar bajo

5 ❑ Defectos de nacimiento

6 ❑ Anemia

7 ❑ Ictericia

8 ❑ Problemas respiratorios

9 ❑ Problemas del corazón

A ❑ Cirugía

B ❑ Infección

C ❑ Otro (Por favor explique):_________________________________________________________

D ❑ No sé

**ALIMENTACIÓN INFANTIL**

**Questions 22-26 Infant Feeding Practices**

Fein SB, Labiner-Wolfe J, Shealy KR, Li R, Chen J, Grummer-Strawn LM: **Infant Feeding Practices Study II: study methods**. *Pediatrics* 2008, **122 Suppl 2**:S28-35.

**27. ¿Su bebé tiene necesidades especiales o problemas médicos?**

0 ❑ No

1 ❑ Sí (*Complete 27A*)

**27A. Por favor explique brevemente: ______________________________________________________________________________________________________________________________________________________________________________________________________________________________________________________________________________**

**¡GRACIAS POR COMPLETAR ESTA ENCUESTA!**

**FELICIDADES POR SU NUEVO BEBÉ.**
