# Supplementary material for: Study Design, Protocol and Profile of the Maternal And Developmental Risks from Environmental and Social Stressors (MADRES) Pregnancy Cohort: a Prospective Cohort Study in Predominantly Low-Income Hispanic Women in Urban Los Angeles
Source: BMC Pregnancy Childbirth. 2019 May 30;19:189. doi: 10.1186/s12884-019-2330-7 (PMC6543670; doi:10.1186/s12884-019-2330-7)
Supplement: Supplementary file 21 — MADRES 1-Month Questionnaire. Questionnaire administered during the 1-month study visit. (DOCX 132 kb) [file 12884_2019_2330_MOESM21_ESM.docx]

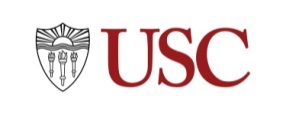
**MADRES Study: One Month Questionnaire**

**Today’s Date:** ________________ **Interviewer Name:** ____________________

**Instructions:** Thank you for participating in the MADRES study. In this interview, I will be asking some questions about you and your baby. Please answer all questions as best as you can, even if you are not completely certain. Be assured that your answers are confidential. Please feel free to interrupt me and ask about anything that is not clear**.**

**CONTACT INFORMATION**

**1. Name:** _________________ _______________ ____________________ ____________________

First Middle Last 1 Last 2

**2. Other names used** (e.g. Maiden name) ­­­­­­­­­­­­­­­­­­­­­­­­­:­___________________________

### **3**. **Your Date of Birth:** **_______/_______/_______**

Month Day Year

**4**. **Baby’s Name:** _________________ ______________ ____________________ _________________

First Middle Last 1 Last 2

### **5**. **Baby’s DOB:** **_______/_______/_______**

Month Day Year

**6**. **Baby’s Gender:** □₁ Female □₂ Male

**7. What is your cell phone number?** ____________________________

□₀ Don’t have a cell phone **(Skip to question #9)**

**8.** **Is this a prepaid cell phone or a permanent phone number?**

□₀ prepaid

□₁ permanent number

**9.** **What is your HOME address (the address at which you spend the most time)?**

Address: ______________________________________________________________________

City: ___________________________State: ________________Zip: _____________________

**9A.** **If moved…When did you move into your new home address?** _______________________

**10. Please tell me the names of other adults living with you:**

Adult#1 First: ______________________Last: ______________________Middle: ______________

Relation to you: ___________________ Cell Phone: ______________________

Adult#2 First: ______________________Last: ______________________Middle: ______________

Relation to you: ___________________ Cell Phone: ______________________

Adult#3 First: ______________________Last: ______________________Middle: ______________

Relation to you: ___________________ Cell Phone: ______________________

**11. What is the phone number for the HOME listed in Question 9?**____________________________

□₀ Don’t have a home phone

**12. Do you live at more than one home?**

□₁ Yes... *Complete questions 13A, 13B and 13C* □₀No… *Go to Question #14*

**13A. What is your second HOME address?**

Address: ______________________________________________________________________

City: ___________________________State: ________________Zip: _____________________

**13A2.** **If moved…When did you move into your new second home address?**  ___________________

**13B. What is the phone number for the HOME listed in Question 13A?** _______________________

□₀ Don’t have a home phone

**13C. How much time do you spend at the address listed in 13A?**

 1%-25% of the time

 26%-50% of the time

**14. A. What is your email address?** _________________________ 0 ❑Don’t have an email address

**B. What is your Facebook username?** ___________________________0 ❑Don’t have Facebook

**C. What is your Twitter handle?** @________________________________0 ❑Don’t have Twitter

**D. What is your Instagram contact name?** _______________________0 ❑Don’t have Instagram

**15.** **A. How do you prefer to be contacted?**

 Phone

 Email
  Text
  Other: ________________

**B. What are the best days to reach you?**

 Monday

 Tuesday
  Wednesday
  Thursday
  Friday
  Saturday
  Sunday

**C. What are the best times to reach you (Monday)?**

 Mornings (8am-12pm)

 Afternoons (12pm-5pm)
  Evenings (5pm-8pm)
  Other: ________________

**D. What are the best times to reach you (Tuesday)?**

 Mornings (8am-12pm)

 Afternoons (12pm-5pm)
  Evenings (5pm-8pm)
  Other: ________________

**E. What are the best times to reach you (Wednesday)?**

 Mornings (8am-12pm)

 Afternoons (12pm-5pm)
  Evenings (5pm-8pm)
  Other: ________________

**F. What are the best times to reach you (Thursday)?**

 Mornings (8am-12pm)

 Afternoons (12pm-5pm)
  Evenings (5pm-8pm)
  Other: ________________

**G. What are the best times to reach you (Friday)?**

 Mornings (8am-12pm)

 Afternoons (12pm-5pm)
  Evenings (5pm-8pm)
  Other: ________________

**H. What are the best times to reach you (Saturday)?**

 Mornings (8am-12pm)

 Afternoons (12pm-5pm)
  Evenings (5pm-8pm)
  Other: ________________

**I. What are the best times to reach you (Sunday)?**

 Mornings (8am-12pm)

 Afternoons (12pm-5pm)
  Evenings (5pm-8pm)
  Other: ________________

**16.** **What is the baby’s father’s name?** □ Don’t know

_________________ _______________ ____________________ ____________________

First Middle Last 1 Last 2

**17A. Do you have a spouse/partner?** 0 ❑ No…*Go to Question 18* 1 ❑ Yes

**17B. What is the name of your spouse/partner**? □ Same as above □ No spouse/partner

_________________ _______________ ____________________ ____________________

First Middle Last 1 Last 2

**18.** **In order to help locate you in case you move and/or change your phone number can you provide us with contact information for your mother and three friends/family members not living with you who would be able to provide us with your new contact information?**

MOTHER’S INFORMATION

First: ______________________Last: ______________________Middle: ______________

Address: ______________________________________________________________________

City: ___________________________State: ________________Zip: _____________________

Cell Phone: ______________________ Home Phone: ______________________

NOK#1

First: ______________________Last: ______________________Middle: ______________

Relation to you: ___________________Email address: ____________________________

Cell Phone: ______________________ Home Phone: ______________________

NOK#2

First: ______________________Last: ______________________Middle: ______________

Relation to you: ___________________Email address: ____________________________

Cell Phone: ______________________ Home Phone: ______________________

NOK#3

First: ______________________Last: ______________________Middle: ______________

Relation to you: ___________________Email address: ____________________________

Cell Phone: ______________________ Home Phone: ______________________

**MAILING ADDRESS**

**19. Do you have a P.O. Box or a mailing address that is different than your home address?**

0 ❑ No
1 ❑ Yes…what is your P.O. Box or mailing address?

Address: _________________________________________________________________

City: ___________________________State: ________________Zip: ________________

**HOUSEHOLD LANGUAGE**

These next questions are about the language you speak most in your home.

**20. How well do you speak English?**
 ❑ Very well
 ❑ Well
 ❑ Not well
 ❑ Not at all

**21. Do you speak a language other than English at home?**
 ❑ Yes
 ❑ No… **Skip** to Question #27

**22. What language(s) do you speak at home? Please select all that apply.** ❑ Spanish
 ❑ Arabic
 ❑ Chinese
 ❑ French
 ❑ French Creole
 ❑ German
 ❑ Italian
 ❑ Korean
 ❑ Polish
 ❑ Russian
 ❑ Tagalog
 ❑ Vietnamese
 ❑ Urdu
 ❑ Punjabi
 ❑ Bengali
 ❑ Farsi
 ❑ Other (please specify):________________________

**23. What is the primary language that you speak at home? By primary, we mean the language you speak most of the time at home.**

❑ English
 ❑ Spanish
 ❑ Arabic
 ❑ Chinese
 ❑ French
 ❑ French Creole
 ❑ German
 ❑ Italian
 ❑ Korean
 ❑ Polish
 ❑ Russian
 ❑ Tagalog
 ❑ Vietnamese
 ❑ Urdu
 ❑ Punjabi
 ❑ Bengali
 ❑ Farsi
 ❑ Other (please specify):____________________________

**24. What is the primary language you read in? By primary, we mean the language you read in most of the time.**

❑ English
 ❑ Spanish
 ❑ Arabic
 ❑ Chinese
 ❑ French
 ❑ French Creole
 ❑ German
 ❑ Italian
 ❑ Korean
 ❑ Polish
 ❑ Russian
 ❑ Tagalog
 ❑ Vietnamese
 ❑ Urdu
 ❑ Punjabi
 ❑ Bengali
 ❑ Farsi
 ❑ Other (please specify):________________________________________

**25. What is the primary language spoken in your home? Here, primary means the language spoken most of the time *by most of the household members*.**

❑ English
 ❑ Spanish
 ❑ Arabic
 ❑ Chinese
 ❑ French
 ❑ French Creole
 ❑ German
 ❑ Italian
 ❑ Korean
 ❑ Polish
 ❑ Russian
 ❑ Tagalog
 ❑ Vietnamese
 ❑ Urdu
 ❑ Punjabi
 ❑ Bengali
 ❑ Farsi
 ❑ Other (please specify):_________________________________

**26. What is the primary language that you or others in your household speak to your child?** ❑ Mostly/All English
 ❑ More English than other language
 ❑ Equally English and other language
 ❑ More other language than English
 ❑ Mostly/All other language

# **ECHO Caregiver Relationship & Family Household Composition DCF**

These next questions are about your relationship to your child, and those who live with your child.

**27.** What is your relationship to the child?

❑ Biological/Birth mother

❑ Biological/Birth father

❑ Stepparent

❑ Adoptive parent

❑ Foster parent

❑ Full Sibling (Same biological mother and father)

❑ Half Sibling (Same biological mother or father)

❑ Non-Biologically Related Sibling (e.g., step sibling, adopted sibling, foster sibling)

❑ Grandparent

❑ Aunt or Uncle

❑ Cousin

❑ Other, please describe:____________________________________

**28.** How much of the caregiving responsibilities do you have for this child?

❑ None or 0%
 ❑A little or about 25%

❑About half or 50%

❑Most or about 75%
 ❑All or 100%

The next set of questions are about all the people living in the child’s home. We are interested in knowing about all children, relatives, a significant partner/spouse, or friends ***who spend at least two nights a week in the house***. If the child lives in two places, please provide as much information about each household as you can.

***29. Excluding you and the child,*** how many people live in your home? Please indicate the total number of children (17years and under) and total number of adults (18years and older).

**Household #1**

- 1. Number of Children:
  2. Number of Adults:  **Household #2:**

1. Number of Children:
2. Number of Adults:

**Household #1**Please answer the following questions for each person living in the child’s home. If the child lives in two places, please complete this section and then continue to the next section regarding household #2.
Please start with the youngest person who lives in the child’s home, and then continue to list each additional person living in the home, up to 9 people.

|  | **Age** | **Gender** | **Relationship to YOU** | **Relationship to CHILD** | **How much of the caregiving responsibilities does this person have for the child?** |
| --- | --- | --- | --- | --- | --- |
|  | *Years, Months* | *1=Male*  *2=Female*  *3=Other*  *4=Prefer not to answer* | *1=Biological Child   2=Step Child   3=Adopted Child  4=Foster Child 5=Spouse/Partner  6=Friend/Roommate   7=Parent   8=Grandparent   9=Sibling   10=Other, please describe* | *1=Biological/Birth mother  2=Biological/Birth father 3=Stepparent 4=Adoptive parent 5=Foster parent 6=Full Sibling (Same biological mother and father) 7=Half Sibling (Same biological mother or father) 8=Non-Biologically Related Sibling (e.g., step sibling, adopted sibling, foster sibling) 9=Grandparent   10=Aunt or Uncle 11=Cousin 12=Other, please describe* | *1= None or 0%  2= A little or about 25%  3= About half or 50%  4= Most or about 75%  5= All or 100%* |
| Person 1 |  |  |  |  |  |
| Person 2 |  |  |  |  |  |
| Person 3 |  |  |  |  |  |
| Person 4 |  |  |  |  |  |
| Person 5 |  |  |  |  |  |
| Person 6 |  |  |  |  |  |
| Person 7 |  |  |  |  |  |
| Person 8 |  |  |  |  |  |
| Person 9 |  |  |  |  |  |

**Household #2**Please answer the following questions for each person living in the child’s household #2.
Please start with the youngest person who lives in the child’s home, and then continue to list each additional person living in the home, up to 9 people.

|  | **Age** | **Gender** | **Relationship to YOU** | **Relationship to CHILD** | **How much of the caregiving responsibilities does this person have for the child?** |
| --- | --- | --- | --- | --- | --- |
|  | *Years, Months* | *1=Male*  *2=Female*  *3=Other*  *4=Prefer not to answer* | *1=Biological Child   2=Step Child   3=Adopted Child  4=Foster Child 5=Spouse/Partner  6=Friend/Roommate   7=Parent   8=Grandparent   9=Sibling   10=Other, please describe* | *1=Biological/Birth mother  2=Biological/Birth father 3=Stepparent 4=Adoptive parent 5=Foster parent 6=Full Sibling (Same biological mother and father) 7=Half Sibling (Same biological mother or father) 8=Non-Biologically Related Sibling (e.g., step sibling, adopted sibling, foster sibling) 9=Grandparent   10=Aunt or Uncle 11=Cousin 12=Other, please describe* | *1= None or 0%  2= A little or about 25%  3= About half or 50%  4= Most or about 75%  5= All or 100%* |
| Person 1 |  |  |  |  |  |
| Person 2 |  |  |  |  |  |
| Person 3 |  |  |  |  |  |
| Person 4 |  |  |  |  |  |
| Person 5 |  |  |  |  |  |
| Person 6 |  |  |  |  |  |
| Person 7 |  |  |  |  |  |
| Person 8 |  |  |  |  |  |
| Person 9 |  |  |  |  |  |

# **Caregiver Occupation and Employment DCF**

**These next questions are about your current occupation and employment status and the current occupation and employment status of your partner or spouse.**

**Section 1: Child’s Primary Caregiver**

**30.** Please select the category that best describes your current work or employment status:

❑Employed by others for wages, full time (30 hours per week or more)

Go to Q31-35

❑Employed by others for wages, part time (29 hours per week or less)

❑Self-employed or family business, for wages

❑Worked without pay in a family business or farm

❑Active Duty in the United States Armed Forces (regular military, National Guard, Military Reserve Unit)

❑Homemaker, not working outside the home

❑Unemployed or laid off, looking for work

❑Unemployed, not currently looking for work …. **Go to question 30b**

❑Prefer not to answer

30b. What is the main reason you have been out of work?
❑On sick or other unpaid leave

❑On layoff

❑Unable to work for health reasons

❑Disabled

❑Retired

❑Taking care of house/family

❑Going to school

❑Other (specify):

❑Prefer not to answer

**31.** What kind of business or industry do you work in? For example, hospital, elementary school, clothing manufacturing, restaurant. If you have more than one job, describe the one at which you work the most hours.
______________________________________________________________________________________________________________________________________

**32.** What kind of work do you do? For example: registered nurse, secretary, teacher, accountant, janitor, auto mechanic. If you had more than one job, describe the one at which you work the most hours.

________________________________________________________________________________________________________________________________________

**33**. During the past 12 months, how many weeks did you work total in **ALL** jobs, including paid vacation time and paid sick leave?
 ❑50 to 52 weeks
 ❑48 – 49 weeks
 ❑40 to 47 weeks
 ❑27 to 39 weeks
 ❑14 to 26 weeks
 ❑13 weeks or less… **Skip to Section 2**

**34**. During the past 12 months in the weeks in which you worked, how many hours per week did you work? _______ (three digit response option, range 1 to 168)

**35**. During the past 12 months in the weeks in which you worked, what hours of the day or shift did you normally work?

❑Daytime (starting in the morning, between 5am and before 12pm)

❑Afternoon/Evening (starting between 12pm and before 9pm)

❑Nighttime (starting 9pm or before 5am)

❑Swing shift

❑Varied hours, rotating shifts, on call

❑Other (specify):

**Section 2:** Partner/Spouse [SKIP IF NO PARTNER/SPOUSE]

Please answer the following questions about your partner/spouse.

**36**. Please select the category that best describes your partner/spouse’s current work or employment status:

❑Employed by others for wages, full time (30 hours per week or more)

❑Employed by others for wages, part time (29 hours per week or less)

Go to Q37-38

❑Self-employed or family business, for wages

❑Worked without pay in a family business or farm

❑Active Duty in the United States Armed Forces (regular military, National Guard, Military Reserve Unit)

❑Homemaker, not working outside the home

❑Unemployed or laid off, looking for work

❑Unemployed, not currently looking for work  **Go to question 36b** ❑Prefer not to answer

**36b.** What is the main reason your partner/spouse has been out of work?

❑On sick or other unpaid leave

❑On layoff

❑Unable to work for health reasons

❑Disabled

❑Retired

❑Taking care of house/family

❑Going to school

❑Other (specify):

❑Prefer not to answer

**37**. What kind of business or industry does your partner/spouse work in? For example, hospital, elementary school, clothing manufacturing, restaurant. If he/she has more than one job, describe the one at which he/she works the most hours.
________________________________________________________________________________________________________________________________________

**38.** What kind of work does your partner/spouse do? For example: registered nurse, secretary, teacher, accountant, janitor, auto mechanic. If he/she had more than one job, describe the one at which he/she works the most hours.

____________________________________________________________________ ____________________________________________________________________

**Caregiver Health Insurance DCF**

The following question is about your current health insurance coverage.

**39.** Which of the following types of health insurance or coverage are you currently covered by? Please select all that apply.
 ❑ Insurance through a current or former employer or union (of yours or another family member’s). This would include COBRA coverage.
 ❑ Insurance purchased directly from an insurance company (by your or another family member).This includes coverage purchased through an exchange or marketplace, such as HealthCare.gov.
 ❑ Medicaid, Medical Assistance (MA), the Children’s Health Insurance Program (CHIP), or any kind of state or government sponsored assistance plan based on income or disability.
 ❑TRICARE or other military healthcare, including VA healthcare.
 ❑Indian Health Service.
 ❑ Any other type of health insurance coverage or health coverage plan (please specify): ________
 ❑ I am not currently covered by health insurance or coverage.
 ❑Don’t know.

**Family Medical History DCF**

The following questions ask about the medical history of your child’s biological family members. This includes his or her birth mother, birth father, grandparents, aunts, uncles, and full siblings (i.e., those who have the same birth mother and birth father).

If you are familiar with the health history of any of the members of the child’s biological or birth family, please answer the following questions about these relatives’ health to the extent that you are able. If you are uncertain of the answer to any question, please select “Don’t Know.”

**40.** Is any information about the ECHO child participant’s biological family medical history known?
 ❑Yes… Continue to Chart
 ❑No… Skip to Question #41
 ❑ Don’t Know

Including living and deceased, have any of the child’s **biological** family members been told by a health care professional that they had any of the following conditions/diseases? If yes to any condition(s) please indicate which family member(s).

| **Condition** | **Yes** | **No** | **Don't Know** | **If yes, please indicate which family member(s):** | | | | | | | | |
| --- | --- | --- | --- | --- | --- | --- | --- | --- | --- | --- | --- | --- |
|  |  |  |  | **Biological Mother** | **Biological Father** | **Biological Sibling** | **Maternal Grand- mother** | **Maternal Grand- father** | **Paternal Grand- mother** | **Paternal Grand- father** | **Maternal Aunt/ Uncle** | **Paternal Aunt/ Uncle** |
| Asthma |  |  |  |  |  |  |  |  |  |  |  |  |
| Eczema (Atopic Dermatitis) |  |  |  |  |  |  |  |  |  |  |  |  |
| Food allergy |  |  |  |  |  |  |  |  |  |  |  |  |
| Hay Fever (Allergic Rhinitis) |  |  |  |  |  |  |  |  |  |  |  |  |
| Anxiety |  |  |  |  |  |  |  |  |  |  |  |  |
| Bipolar Disorder |  |  |  |  |  |  |  |  |  |  |  |  |
| Depression |  |  |  |  |  |  |  |  |  |  |  |  |
| Eating Disorder |  |  |  |  |  |  |  |  |  |  |  |  |
| Schizophrenia |  |  |  |  |  |  |  |  |  |  |  |  |
| Alcoholism or other Substance abuse |  |  |  |  |  |  |  |  |  |  |  |  |
| Attention Deficit/Hyperactivity Disorder (ADHD) |  |  |  |  |  |  |  |  |  |  |  |  |
| Autism Spectrum Disorder (ASD) |  |  |  |  |  |  |  |  |  |  |  |  |
| Learning disability (e.g., dyslexia) |  |  |  |  |  |  |  |  |  |  |  |  |
| Intellectual disability (e.g., mental retardation) |  |  |  |  |  |  |  |  |  |  |  |  |
| Epilepsy or seizure disorder |  |  |  |  |  |  |  |  |  |  |  |  |
| High Cholesterol (Hyperlipidemia) |  |  |  |  |  |  |  |  |  |  |  |  |
| Hypertension/ High Blood Pressure |  |  |  |  |  |  |  |  |  |  |  |  |
| Type 1 Diabetes |  |  |  |  |  |  |  |  |  |  |  |  |
| Type 2 Diabetes |  |  |  |  |  |  |  |  |  |  |  |  |
| Thyroid disorder |  |  |  |  |  |  |  |  |  |  |  |  |
| Coronary artery disease (e.g., heart attack, angina) |  |  |  |  |  |  |  |  |  |  |  |  |
| Any other heart disease |  |  |  |  |  |  |  |  |  |  |  |  |
| Stroke |  |  |  |  |  |  |  |  |  |  |  |  |
| Breast cancer |  |  |  |  |  |  |  |  |  |  |  |  |
| Colon cancer |  |  |  |  |  |  |  |  |  |  |  |  |
| Other Cancer (please specify type) |  |  |  |  |  |  |  |  |  |  |  |  |
| Deceased |  |  |  |  |  |  |  |  |  |  |  |  |

# **ECHO Income, Assistance, Financial Strain Questions**

These next questions are about your family income and any financial assistance you may receive.

**Section 1: Income**
Please think about your **total combined household income** during the last calendar year for all members of your household.

**41**. How many household members are supported by your total combined household income?

____________(Number)
 ❑Don’t know
 ❑Prefer not to answer

**42**. How many of those people are children? Please include anyone under 18 years or anyone older than 18 years and in high school.
 ____________(Number)
 ❑Don’t know
 ❑Prefer not to answer

**43.** At any time DURING THE PAST 12 MONTHS, even for one month, did anyone in your primary household receive income from any of the following sources? Primary household includes all children, relatives, a significant partner/spouse or friends living in your household ***who spend at least two nights a week in the house***. Please select all that apply.

❑Wages, salary, commissions, bonuses, or tips from all jobs

❑Self-employment income from own nonfarm businesses or farm businesses, including proprietorships and partnerships

❑Interest, dividends, net rental income, royalty income, or income from estates and trusts

❑Social Security or Railroad Retirement

❑Supplemental Security Income (SSI)

❑Any public assistance or welfare payments from the state or local welfare office

❑Retirement, survivor, or disability pensions

❑Any other sources of income received regularly such as Veterans’ (VA) payments, unemployment compensation, child support or alimony. Do NOT include lump sum payments such as money from an inheritance or the sale of a home

**44**. At any time DURING THE last calendar year, even for one month, did anyone in your household receive:

| Cash assistance from a government welfare program (e.g., Temporary Assistance for Needy Families [TANF], formerly known as Aid to Families with Dependent Children | ❑ Yes  ❑ No  ❑ Don’t Know  ❑ Prefer not to answer |
| --- | --- |
| Food Stamps or Supplemental Nutrition Assistance Program (SNAP) benefits | ❑ Yes  ❑ No  ❑ Don’t Know  ❑ Prefer not to answer |
| Benefits from the Woman, Infants, and Children (WIC) Program | ❑ Yes  ❑ No  ❑ Don’t Know  ❑ Prefer not to answer |

**45.** Which of the following categories best represents your **total** combined household income *during the last calendar year*? This includes all money earned by primary household members who contribute to household expenses (e.g., spouse/partner, parents). Be sure to include total wages, salaries, self-employment income after expenses, government assistance of any kind, interest and dividends, etc., before taxes.

❑ LESS THAN $4,999
❑$5,000-$9,999

❑$10,000-$19,999
 ❑$20,000-$29,999
 ❑$30,000-$39,999
 ❑$40,000-$49,999
 ❑$50,000-$74,999
 ❑$75,000-$99,999
 ❑$100,000-$199,999
 ❑$200,000 OR MORE
 ❑Don’t know
 ❑Prefer not to answer

**46.** During the last calendar year, how often do you put off buying something you need - such as food, clothing, medical care, or housing - because you don't have money? Would you say...

❑Never

❑ Rarely
❑Occasionally
❑Frequently
❑All the time
❑Prefer not to answer

**47**. During the last calendar year, how much difficulty have you had paying bills? Would you say...
❑ No difficulty at all

❑ A little difficulty
❑ Some difficulty
❑ Quite a bit of difficulty
❑ A great deal of difficulty
❑ Prefer not to answer

**48**. Thinking about the end of each month over the last calendar year, did you generally end up with...
❑ More than enough money left over
❑ Some money left over
❑ Just enough to make ends meet
❑ Not enough to make ends meet
❑ Prefer not to answer

**49**. Have you set aside emergency or rainy day funds that would cover your expenses for 3 months, in case of sickness, job loss, economic downturn, or other emergencies?
 ❑ Yes
 ❑ No
 ❑ Prefer not to answer

**MOTHER’S FAMILY PLANNING AND WEIGHT LOSS**

**50.** Do you intend to have an additional child within the next 12 months?

1 ❑ Yes
 0 ❑ No

**51**. Do you intend to go back onto birth control?

1 ❑ Yes
 0 ❑ No

**52.** How much weight did you gain during your pregnancy?

1 ❑ Under 10 lbs.

2 ❑ 10-20 lbs.

3 ❑ 20-30 lbs.

4 ❑ More than 30 lbs.

9 ❑ Do not know

**53.** How concerned are you with losing your pregnancy weight?

1 ❑ Not at all concerned

2 ❑ Not very concerned

3 ❑ Somewhat concerned

4 ❑ Very concerned

**BABY’S HEALTH**

**54.** When your baby was discharged from the hospital after you gave birth, did your baby have more than a 10% drop in weight?

0 ❑ No
1 ❑ Yes
9 ❑ Don’t Know

**55.** Did your baby go to a doctor for a one-week visit after birth?
 0 ❑ No…**SKIP** to Question 57
 1 ❑ Yes

**56.** Did your baby’s doctor say that your baby was gaining weight regularly and that growth was going according to plan?
1 ❑ Yes
0 ❑ No
9 ❑ Don’t Know

**57**. Did your baby receive any antibiotics since birth?
0 ❑ No
1 ❑ Yes, once
9 ❑ Yes, more than once

**BREASTFEEDING**

**Questions 58-62 Breastfeeding and Infant Feeding**

Fein SB, Labiner-Wolfe J, Shealy KR, Li R, Chen J, Grummer-Strawn LM: **Infant Feeding Practices Study II: study methods**. *Pediatrics* 2008, **122 Suppl 2**:S28-35.

**GLOBAL HEALTH QUESTIONS**

**Questions 63-72 PROMIS® Scale v1.2 – Global Health**

Cella, D., Riley, W., Stone, A., Rothrock, N., Reeve, B., Yount, S., Amtmann, D., Bode, R., Buysse, D. J., Choi, S. W., Cook, K. F., DeVellis, R., DeWalt, D., Fries, J. F., Gershon, R., Hahn, E., Pilkonis, P., Revicki, D., Rose, M., Weinfurt, K., & Hays, R. D. on behalf of the PROMIS Cooperative Group. (2010). Initial item banks and first wave testing of the Patient–Reported Outcomes Measurement Information System (PROMIS) network: 2005–2008. *Journal of Clinical Epidemiology, 63*(11), 1179-94.

**PERCEIVED IMMIGRATION POLICY EFFECTS**

**73. These next questions will ask about how often you have experienced certain occurrences based off your ethnicity or immigration status. Please tell me one answer for each question according to your experiences.**

|  | Never | A little bit | Once in a while | Often | Always |
| --- | --- | --- | --- | --- | --- |
| **A**. Were you treated unfairly at a restaurant or store? |  |  |  |  |  |
| **B**. Were you silenced by others or felt that your opinion did not matter? |  |  |  |  |  |
| **C.** Did others act like they had the right to treat you unfairly or poorly? |  |  |  |  |  |
| **D**. Were you treated like a criminal based on who you are? |  |  |  |  |  |
| **E**. Were treated poorly because you did not speak English? |  |  |  |  |  |
| **F**. Did you feel ignored when seeking help? |  |  |  |  |  |
| **G**. Were you humiliated because of who you are? |  |  |  |  |  |
| **H**. Did you feel that you were being exploited or taken advantage of at work? |  |  |  |  |  |
| **I.** Did you feel that you had no liberty and needed to stay home? |  |  |  |  |  |
| **J.** Did you avoid certain locations like parks and neighborhoods because you did not feel safe? |  |  |  |  |  |
| **K**. Did you fear being deported or detained? |  |  |  |  |  |
| **L**. Did you feel unsafe when leaving your home? |  |  |  |  |  |
| **M.** Did you feel that that you had no rights? |  |  |  |  |  |
| **N.** Did you worry about the impact of these policies on your family? |  |  |  |  |  |
| **O**. Did you fear that you or a family member would be reported to immigration officials? |  |  |  |  |  |
| **P**. Did you worry about family separation due to deportation? |  |  |  |  |  |

**74. Do you have other children?** ❑Yes.. Continue to chart
 ❑ No.. Skip to Question #74

|  | Never | A little bit | Once in a while | Often | Always |
| --- | --- | --- | --- | --- | --- |
| **A**. Have your children been stressed about family members being deported or detained? |  |  |  |  |  |
| **B**. Have your children felt unsafe due to immigration policies? |  |  |  |  |  |
| **C.** Have you been concerned that your children were having emotional problems due to immigration policies? |  |  |  |  |  |
| **D**. Have your children feared authorities due to immigration policies? |  |  |  |  |  |
| **E**. Have your children had difficulties focusing in school due to immigration policies? |  |  |  |  |  |
| **F**. Have your children been treated differently in school due to immigration policies or negative attitudes toward immigrants? |  |  |  |  |  |
| **G**. Have your children been denied other services due to immigration policies or negative attitudes toward immigrants? |  |  |  |  |  |

**SOCIAL SUPPORT QUESTIONS**

**Question 75 Emotional Support, Instrumental Support, and Informational Support PROMIS® Scale**
 Cella, D., Riley, W., Stone, A., Rothrock, N., Reeve, B., Yount, S., Amtmann, D., Bode, R., Buysse, D. J., Choi, S. W., Cook, K. F., DeVellis, R., DeWalt, D., Fries, J. F., Gershon, R., Hahn, E., Pilkonis, P., Revicki, D., Rose, M., Weinfurt, K., & Hays, R. D. on behalf of the PROMIS Cooperative Group. (2010). Initial item banks and first wave testing of the Patient–Reported Outcomes Measurement Information System (PROMIS) network: 2005–2008. *Journal of Clinical Epidemiology, 63*(11), 1179-94.

**POSTPARTUM DISTRESS MEASURE**

**Questions 76-84 Postpartum Distress Measure**
 Allison KC, Wenzel A, Kleiman K, Sarwer DB: **Development of a brief measure of postpartum distress**. *J Womens Health (Larchmt)* 2011, **20**(4):617-623.
