# Supplementary material for: Study Design, Protocol and Profile of the Maternal And Developmental Risks from Environmental and Social Stressors (MADRES) Pregnancy Cohort: a Prospective Cohort Study in Predominantly Low-Income Hispanic Women in Urban Los Angeles
Source: BMC Pregnancy Childbirth. 2019 May 30;19:189. doi: 10.1186/s12884-019-2330-7 (PMC6543670; doi:10.1186/s12884-019-2330-7)
Supplement: Supplementary file 22 — MADRES 1-Month Questionnaire_Spanish. Spanish questionnaire administered during the 1-month study visit. (DOCX 112 kb) [file 12884_2019_2330_MOESM22_ESM.docx]

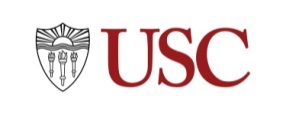
**MADRES Study: One Month Questionnaire**

**Today’s Date:** ________________ **Interviewer Name:** ____________________

**Instrucciones:** Gracias por aceptar participar en el estudio MADRES. Durante esta entrevista le hare preguntas sobre usted y su bebe. Por favor responda a todas las preguntas de este cuestionario, aún si no está completamente segura de la respuesta. Le aseguramos que sus respuestas serán confidenciales**.** Por favor, con toda confianza, siéntase libre de interrumpirme y hacerme cualquier pregunta que tenga.

**INFORMACION DE CONTACTO**

**1. Nombre:** ________________ __________________ __________________ ____________________

Nombre 2do Nombre Apellido 1 Apellido 2

**2. Otros nombres que haya usado** (ej. nombre de soltera) ­­­­­­­­­­­­­­­­­­­­­­­­­:­___________________________

**3**. **Fecha de nacimiento:** _**______/_______/_______**

Mes Día Año

**4**. **Nombre del Bebé:** __________________ ________________ __________________ _________________

Nombre 2do Nombre Apellido 1 Apellido 2

**5. Fecha de nacimiento del Bebé:** **_______/_______/_______**

Mes Día Año

**6**. **Género del Bebé:** □₁ Femenino □₂ Masculino

**7. ¿Cuál es su número de celular?** ____________________________

□₀ No tengo teléfono celular **(Skip to question #9)**

**8.** **¿Es un celular pre pagado o es un número fijo de celular?**

□₀ Pre pagado

□₁ Número fijo

**9.** **¿Cuál es su dirección de domicilio? (la dirección donde usted pasa la mayoría del tiempo):**

Dirección: ________________________________________________________________________

Ciudad: _____________________Estado: ________________Código Postal: ___________________

**9A.** **If moved…¿Cuándo se mudó a su nueva dirección?** _______________________

**10. Por favor dígame los nombres de otros adultos que viven con usted:**

Adult#1 Nombre: ______________________Apellido: ______________________2do Nombre: ______________

Relación: ___________________ Número de Celular: ______________________

Adult#2 Nombre: ______________________Apellido: ______________________2do Nombre: ______________

Relación: ___________________ Número de Celular: ______________________

Adult#3 Nombre: ______________________Apellido: ______________________2do Nombre: ______________

Relación: ___________________ Número de Celular: ______________________

**11. ¿Cuál es el número de teléfono para el domicilio dado en la Pregunta 9?**______________________

□₀ No tengo teléfono de casa

**12. ¿Vive en más de una casa?**

□₁ Sí... *Complete questions 13A, 13B and 13C* □₀No…  *Go to question #14*

**13A. ¿Cuál es la dirección de su segundo domicilio?**

Dirección: _________________________________________________________________________

Ciudad: ______________________Estado: ________________ Código Postal: __________________

**13A2.** **If moved…¿Cuándo se mudó a su nuevo segundo domicilio?** _______________________

**13B. ¿Cuál es el número de teléfono para el domicilio dado en la Pregunta 13A?** _____________________

□₀ No tengo teléfono de casa

**13C. ¿Cuánto tiempo pasa usted en la dirección dada en la pregunta 13A?**

1%-25% del tiempo

26%-50% del tiempo

**14. A. ¿Cuál es su correo electrónico?** _________________________ 0 ❑No tengo correo electrónico

**B. ¿Cuál es su nombre de usuario en Facebook?** ___________________________0 ❑No tengo Facebook

**C. ¿Cuál es su nombre de usuario en Twitter?** @___________________________0 ❑No tengo Twitter

**D. ¿Cuál es su nombre de contacto para Instagram?** ____________________0 ❑No tengo Instagram

**15. A. ¿Cómo prefiere ser contactada?**

Teléfono

Correo electrónico

Texto

Otro: ________________

**B. ¿Cuáles son los mejores días para contactarla?**

lunes

martes

miércoles

jueves

viernes

sábado

domingo

**C. ¿Cuáles son las mejores horas para contactarla (lunes)?**

En la mañana (8am-12pm)

En la tarde (12pm-5pm)

En la noche (5pm-8pm)

Otro: ______________

**D. ¿Cuáles son las mejores horas para contactarla (martes)?**

Mañana (8am-12pm)

Tarde (12pm-5pm)

Noche (5pm-8pm)

Otro: ______________

**E. ¿Cuáles son las mejores horas para contactarla (miércoles)?**

Mañana (8am-12pm)

Tarde (12pm-5pm)

Noche (5pm-8pm)

Otro: ______________

**F. ¿Cuáles son las mejores horas para contactarla (jueves)?**

Mañana (8am-12pm)

Tarde (12pm-5pm)

Noche (5pm-8pm)

Otro: ______________

**G. ¿Cuáles son las mejores horas para contactarla (viernes)?**

Mañana (8am-12pm)

Tarde (12pm-5pm)

Noche (5pm-8pm)

Otro: ______________

**H. ¿Cuáles son las mejores horas para contactarla (sábado)?**

Mañana (8am-12pm)

Tarde (12pm-5pm)

Noche (5pm-8pm)

Otro: ______________

**I . ¿Cuáles son las mejores horas para contactarla (domingo)?**

Mañana (8am-12pm)

Tarde (12pm-5pm)

Noche (5pm-8pm)

Otro: ______________

**16.** **¿Cómo se llama el papa del bebé?** □ No sé

_________________ _______________ ____________________ ____________________

Nombre 2do Nombre Apellido 1 Apellido 2

**17A.** **¿Tiene usted esposo o pareja?** 0 ❑ No…*Go to Question* 18 1 ❑ Sí

**17B. ¿Cómo se llama su esposo/pareja?**

_________________ _______________ ____________________ ____________________

Nombre 2do Nombre Apellido 1 Apellido 2

**18.** **Para poder localizarla en caso de que se mude o cambie su número de teléfono, ¿nos puede dar la información de su madre y tres amigos o familiares que no vivan con usted que nos podrían dar su información nueva?**

INFORMACION DE SU MADRE

Nombre: ______________________Apellido: ______________________2do Nombre: ______________

Dirección: _________________________________________________________________________

Ciudad: ______________________Estado: ________________ Código Postal: __________________

Número de Celular: ______________________ Número de teléfono de Casa: ______________________

NOK#1

Nombre: ______________________Apellido: ______________________2do Nombre: ______________

Relación: ___________________Correo Electrónico: ____________________________

Número de Celular: ______________________Número de teléfono de Casa: ______________________

NOK#2

Nombre: ______________________Apellido: ______________________2do Nombre: ______________

Relación: ___________________Correo Electrónico: ____________________________

Número de Celular: ______________________Número de teléfono de Casa: ______________________

NOK#3

Nombre: ______________________Apellido: ______________________2do Nombre: ______________

Relación: ___________________Correo Electrónico: ____________________________

Número de Celular: ______________________Número de teléfono de Casa: ______________________

**DIRECCIÓN DE ENVIO**

**19. ¿Tiene una dirección postal o postal diferente a la dirección de su domicilio?**

0 ❑ No

1 ❑ Sí… ¿cuál es su dirección postal?

Dirección: _________________________________________________________________________

Ciudad: ______________________Estado: ________________ Código Postal: __________________

**HOUSEHOLD LANGUAGE**

Las siguientes preguntas se tratan del idioma que más habla en su hogar.

**20. ¿Qué tan bien habla inglés?**
 ❑ Muy bien
 ❑ Bien
 ❑ No muy bien
 ❑ Para nada

**21. ¿Habla algún otro idioma que no sea ingles en su hogar?**
 ❑ Sí
 ❑ No… **Skip** to Question #27

**22. ¿Qué idioma(s) habla en su hogar? Por favor seleccione todo lo que corresponda.** ❑ Español
 ❑ Árabe
 ❑ Chino
 ❑ Francés
 ❑ Francés criollo
 ❑ Alemán
 ❑ Italiano
 ❑ Coreano
 ❑ Polaco
 ❑ Ruso
 ❑ Tagalo
 ❑ Vietnamita
 ❑ Urdu
 ❑ Punjabi
 ❑ Bengalí
 ❑ Farsi
 ❑ Otro (especifique): ________________________

**23. ¿Cuál es el idioma principal que habla en casa? Por principal, nos referimos al idioma que habla la mayor parte del tiempo en el hogar.**

❑ Inglés
 ❑ Español
 ❑ Árabe
 ❑ Chino
 ❑ Francés
 ❑ Francés criollo
 ❑ Alemán
 ❑ Italiano
 ❑ Coreano
 ❑ Polaco
 ❑ Ruso
 ❑ Tagalo
 ❑ Vietnamita
 ❑ Urdu
 ❑ Punjabi
 ❑ Bengalí
 ❑ Farsi
 ❑ Otro (especifique): ________________________

**24. ¿Cuál es el idioma principal en que lee? Por principal, nos referimos al idioma en que lee la mayor parte del tiempo.**

❑ Inglés
 ❑ Español
 ❑ Árabe
 ❑ Chino
 ❑ Francés
 ❑ Francés criollo
 ❑ Alemán
 ❑ Italiano
 ❑ Coreano
 ❑ Polaco
 ❑ Ruso
 ❑ Tagalo
 ❑ Vietnamita
 ❑ Urdu
 ❑ Punjabi
 ❑ Bengalí
 ❑ Farsi
 ❑ Otro (especifique): ________________________

**25. ¿Cuál es el idioma principal que se habla en su hogar? Aquí, principal significa el idioma hablado la mayor parte del tiempo *por la mayoría de los miembros del hogar.***

❑ Inglés

❑ Español
 ❑ Árabe
 ❑ Chino
 ❑ Francés
 ❑ Francés criollo
 ❑ Alemán
 ❑ Italiano
 ❑ Coreano
 ❑ Polaco
 ❑ Ruso
 ❑ Tagalo
 ❑ Vietnamita
 ❑ Urdu
 ❑ Punjabi
 ❑ Bengalí
 ❑ Farsi
 ❑ Otro (especifique): ________________________

**26. ¿Cuál es el idioma principal en que usted u otras personas en su hogar le hablan a su hijo/a?** ❑ Mayormente/Todo inglés
 ❑ Mas ingles que otro idioma
 ❑ Igualmente inglés y otro idioma
 ❑ Mas otro idioma que inglés

❑ Mayormente/Todo otro idioma

# **ECHO Caregiver Relationship & Family Household Composition DCF**

Las siguientes preguntas son sobre su parentesco con su hijo/a y con quienes viven con él/ella.

**27.** ¿Cuál es su parentesco con el/la niño/a?

❑ Madre biológica

❑ Padre biológico

❑ Padrastro/madrastra

❑ Padre adoptivo

❑ Padre de crianza / padre “foster”

❑ Hermano/a completo (La misma madre biológica y el mismo padre biológico)

❑ Medio hermano/a (La misma madre biológica o el mismo padre biológico)

❑ Hermanos no relacionados biológicamente (p. ej., hermanastro, hermano adoptivo, hermano de crianza “foster”)

❑ Abuelo/a

❑ Tía o tío

❑ Primo/a

❑ Otro, por favor describa: ____________________________________

**28.** ¿Que cantidad de las responsabilidades de cuidado tiene usted para este niño?

❑ Nada o 0%
 ❑Un poco o casi 25%

❑Casi la mitad o 50%

❑La mayoría o casi 75%
 ❑Toda o 100%

Las siguientes preguntas son sobre las personas que viven en el hogar del niño/a. Estamos interesados en saber de todos los niños, parientes, esposo/pareja significativa o amigos ***que pasan al menos dos noches a la semana en la casa***. Si el niño/a vive en dos lugares, por favor denos la mayor cantidad de información posible sobre cada hogar que usted pueda.

***29. Excluyendo a usted y su niño/a,*** ¿cuántas personas viven en su hogar? Por favor indique el número total de niños (menores de 17 años) y el número total de adultos (mayores de 18 años).

**Hogar #1**

- 1. Número de niños:
  2. Número de adultos:  **Hogar #2:**

1. Número de niños:
2. Número de adultos:

**Hogar #1**Por favor responda las siguientes preguntas para cada persona que vive en el hogar del niño/a. Si el niño/a vive en dos lugares, por favor complete esta sección y luego continúe a la siguiente sección con respecto al hogar #2. Por favor comience con la persona más joven que vive en el hogar del niño/a y luego continúe enumerando cada persona adicional que vive en el hogar, hasta 9 personas.

|  | **Edad** | **Género** | **Parentesco a USTED** | **Parentesco al NIÑO/A** | **¿Qué cantidad de las responsabilidades de cuidado tiene esta persona para este niño/a?** |
| --- | --- | --- | --- | --- | --- |
|  | *Años, Meses* | *1=Masculino*  *2=Femenino*  *3=Otro*  *4=Prefiero no responder* | *1=Hijo biológico   2=Hijastro   3=Hijo adoptivo  4=Hijo de crianza/ hijo “foster” 5=Esposo/Pareja  6=Amigo/Compañero de cuarto  7=Padre   8=Abuelo/a  9=Hermano/a   10=Otro, por favor describa* | *1=* *Madre biológica 2=Padre biológico 3=* *Padrastro/madrastra 4=Padre adoptivo 5=Padre de crianza / padre “foster” 6=Hermano/a completo (La misma madre biológica y el mismo padre biológico) 7* *Medio* *hermano/a (La misma madre biológica o el mismo padre biológico) 8=Hermanos no relacionados biológicamente (p. ej., hermanastro, hermano adoptivo, hermano de crianza “foster”) 9=Abuelo/a   10=Tía o tío 11=Primo/a 12=* *Otro, por favor describa* | *1=Nada o 0% 2 Un poco o casi 25% 3=Casi la mitad o 50% 4=La mayoría o casi 75% 5=Toda o 100%* |
| Persona 1 |  |  |  |  |  |
| Persona 2 |  |  |  |  |  |
| Persona 3 |  |  |  |  |  |
| Persona 4 |  |  |  |  |  |
| Persona 5 |  |  |  |  |  |
| Persona 6 |  |  |  |  |  |
| Persona 7 |  |  |  |  |  |
| Persona 8 |  |  |  |  |  |
| Persona 9 |  |  |  |  |  |

**Hogar #2**Por favor responda las siguientes preguntas para cada persona que vive en el hogar #2 del niño/a.
Por favor comience con la persona más joven que vive en el hogar del niño/a y luego continúe enumerando cada persona adicional que vive en el hogar, hasta 9 personas.

|  | **Edad** | **Género** | **Parentesco a USTED** | **Parentesco al NIÑO/A** | **¿Qué cantidad de las responsabilidades de cuidado tiene esta persona para este niño/a?** |
| --- | --- | --- | --- | --- | --- |
|  | *Años, Meses* | *1=Masculino*  *2=Femenino*  *3=Otro*  *4=Prefiero no responder* | *1=Hijo biológico  2=Hijastro  3=Hijo adoptivo  4=Hijo de crianza/ hijo “foster” 5=Esposo/Pareja  6=Amigo/Compañero de cuarto 7=Padre  8=Abuelo/a 9=Hermano/a  10=Otro, por favor describa* | *1= Madre biológica 2=Padre biológico 3= Padrastro/madrastra 4=Padre adoptivo 5=Padre de crianza / padre “foster” 6=Hermano/a completo (La misma madre biológica y el mismo padre biológico) 7 Medio hermano/a (La misma madre biológica o el mismo padre biológico) 8=Hermanos no relacionados biológicamente (p. ej., hermanastro, hermano adoptivo, hermano de crianza “foster”) 9=Abuelo/a  10=Tía o tío 11=Primo/a 12= Otro, por favor describa* | *1=Nada o 0% 2 Un poco o casi 25% 3=Casi la mitad o 50% 4=La mayoría o casi 75% 5=Toda o 100%* |
| Persona 1 |  |  |  |  |  |
| Persona 2 |  |  |  |  |  |
| Persona 3 |  |  |  |  |  |
| Persona 4 |  |  |  |  |  |
| Persona 5 |  |  |  |  |  |
| Persona 6 |  |  |  |  |  |
| Persona 7 |  |  |  |  |  |
| Persona 8 |  |  |  |  |  |
| Persona 9 |  |  |  |  |  |

# **Caregiver Occupation and Employment DCF**

**Las siguientes preguntas son sobre su ocupación actual y su estado de empleo y la ocupación actual y el estado de empleo de su pareja o esposo.**

**Section 1: Child’s Primary Caregiver**

**30.** Por favor seleccione la categoría que mejor describe su trabajo actual o estado de empleo:

❑Empleado por otros por salario, tiempo completo (30 horas por semana o más)

Go to Q31-35

❑Empleado por otros por salario, tiempo parcial (29 horas por semana o menos)

❑Trabajo por cuenta propia o negocio familiar, por salario

❑Trabaje sin pago en un negocio o granja familiar

❑ Servicio activo en las Fuerzas Armadas de los Estados Unidos (militar regular,

Guardia Nacional, Unidad de Reserva Militar)

❑ Ama/o de casa, no trabajando fuera del hogar

❑Desempleado/a o despedido/a, buscando trabajo

❑Desempleado/a, no buscando trabajo actualmente…. **Go to question 30b**

❑Prefiero no responder

30b. ¿Cuál es la razón principal por la que ha estado sin trabajo?
❑En permiso de enfermedad o permiso sin pago

❑Despedido/a

❑ Incapaz de trabajar por razones de salud

❑ Discapacitado/a

❑Retirado/a

❑Cuidando la casa/familia

❑Yendo a la escuela

❑Otro (especifique):

❑ Prefiero no responder

**31.** ¿En qué tipo de industria o negocio trabaja? Por ejemplo, hospital, escuela primaria, fabricación de ropa, restaurante. Si tiene más de un trabajo, describa aquel en el que trabaja la mayor cantidad de horas.

______________________________________________________________________________________________________________________________________

**32.** ¿Qué tipo de trabajo hace usted? Por ejemplo: enfermera registrada, secretaria, maestro, contador, conserje, mecánico de automóviles. Si tenía más de un trabajo, describa aquel en el que trabaja la mayor cantidad de horas.

________________________________________________________________________________________________________________________________________

**33**. Durante los últimos 12 meses, ¿cuántas semanas trabajó en total en TODOS los trabajos, incluyendo el tiempo de vacaciones pagadas y en permiso por enfermedad con sueldo?
 ❑50 a 52 semanas
 ❑48 – 49 semanas
 ❑40 a 47 semanas
 ❑27 a 39 semanas
 ❑14 a 26 semanas
 ❑13 semanas o menos… **Skip to Section 2**

**34**. Durante los últimos 12 meses en las semanas en que trabajó, ¿cuántas horas por semana trabajó?_______ (three digit response option, range 1 to 168)

**35**. Durante los últimos 12 meses en las semanas en las que trabajó, ¿qué horas del día o turno normalmente trabajó?

❑ Durante el día (comenzando en la mañana, entre las 5 a.m. y antes de las 12 p.m.)

❑Tarde (comenzando entre las 12pm y antes de las 9pm)

❑Noche (comenzando 9pm o antes de las 5am)

❑Segundo turno

❑Horas variadas, turnos rotativos, en guardia

❑Otro (especifique):

**Section 2:** Partner/Spouse [SKIP IF NO PARTNER/SPOUSE]

Por favor responda las siguientes preguntas sobre su pareja/esposo.

**36**. Seleccione la categoría que mejor describa el trabajo actual o el estado laboral de su pareja/esposo:

❑Empleado por otros por salario, tiempo completo (30 horas por semana o más)

Go to Q31-35

❑Empleado por otros por salario, tiempo parcial (29 horas por semana o menos)

❑Trabajo por cuenta propia o negocio familiar, por salario

❑Trabaje sin pago en un negocio o granja familiar

❑ Servicio activo en las Fuerzas Armadas de los Estados Unidos (militar regular,

Guardia Nacional, Unidad de Reserva Militar)

❑ Ama/o de casa, no trabajando fuera del hogar

❑Desempleado/a o despedido/a, buscando trabajo

❑Desempleado/a, no buscando trabajo actualmente…. **Go to question 36b**

❑Prefiero no responder

**36b.** ¿Cuál es la razón principal por la que su pareja/esposo ha estado sin trabajo?

❑En permiso de enfermedad o permiso sin pago

❑Despedido/a

❑ Incapaz de trabajar por razones de salud

❑ Discapacitado/a

❑Retirado/a

❑Cuidando la casa/familia

❑Yendo a la escuela

❑Otro (especifique):

❑ Prefiero no responder

**37**. ¿En qué tipo de industria o negocio trabaja su pareja/esposo? Por ejemplo, hospital, escuela primaria, fabricación de ropa, restaurante. Si su pareja/esposo tiene más de un trabajo, describa aquel en el que trabaja la mayor cantidad de horas.

_______________________________________________________________________________________________________________________________________

**38.** ¿Qué tipo de trabajo hace su pareja/esposo? Por ejemplo: enfermera/o registrada/o, secretario/a, maestro/a, contador/a, conserje, mecánico de automóviles. Si su pareja/esposo tenía más de un trabajo, describa aquel en el que trabaja la mayor cantidad de horas.

____________________________________________________________________ ____________________________________________________________________

**Caregiver Health Insurance DCF**

La siguiente pregunta es sobre su cobertura actual de seguro médico.

**39.** ¿Por cuáles de los siguientes tipos de seguro médico o coberturas de salud está usted cubierta actualmente? Seleccione todo lo que aplique.

❑ Seguro a través de un empleador o sindicato actual o anterior (del suyo o de otro miembro de la familia). Esto incluiría la cobertura COBRA

❑Seguro adquirido directamente de una compañía de seguros (por usted u otro miembro de la familia). Esto incluye la cobertura comprada a través de un mercado o un intercambio, como HealthCare.gov.

❑ Medicaid, Asistencia Médica (MA), el Programa de Seguro de Salud para Niños (CHIP), o cualquier tipo de plan de asistencia patrocinado por el estado o gobierno basado en ingresos o discapacidad.

❑ TRICARE u otra asistencia médica militar, incluida la atención médica de VA
 ❑ Servicio de Salud Indígena (Indian Health Service)
 ❑ Cualquier otro tipo de cobertura de seguro de salud o plan de cobertura de salud (por favor especifique):

__________________________________
❑ Actualmente no estoy cubierto por seguro médico o cobertura de salud
❑ No sé

**Family Medical History DCF**

Las siguientes preguntas son sobre el historial médico de los miembros de la familia biológica de su hijo. Esto incluye a su madre biológica, padre biológico, abuelos, tías, tíos y hermanos completos (es decir, aquellos que tienen la misma madre biológica y el mismo padre biológico).

Si está familiarizado con el historial de salud de cualquiera de los miembros de la familia biológica del niño, responda las siguientes preguntas sobre la salud de estos parientes en la medida de sus posibilidades. Si no está seguro de la respuesta a alguna pregunta, seleccione "No sé".

**40**¿Se conoce alguna información sobre el historial médico de la familia biológica del niño participante de ECHO?
 ❑Sí… Continue to Chart
 ❑No… Skip to Question #41
 ❑ No sé

Incluyendo vivos y fallecidos, ¿le ha dicho un profesional de la salud a alguno de los familiares biológicos del niño que tenía alguna de las siguientes afecciones / enfermedades? En caso afirmativo a alguna condición, indique qué miembro(s) de la familia.

| **Condición** | **Sí** | **No** | **No sé** | **En caso afirmativo, indique qué miembro(s) de la familia:** | | | | | | | | |
| --- | --- | --- | --- | --- | --- | --- | --- | --- | --- | --- | --- | --- |
|  |  |  |  | **Madre**  **Biológica** | **Padre**  **Biológico** | **Hermano**  **Biológico** | **Abuela**  **Materna** | **Abuelo**  **Materno** | **Abuela**  **Paterna** | **Abuelo**  **Paterno** | **Tía/tío**  **Materno** | **Tía/tío**  **Paterno** |
| Asma |  |  |  |  |  |  |  |  |  |  |  |  |
| Eczema (Dermatitis atópica)  ) |  |  |  |  |  |  |  |  |  |  |  |  |
| Alergia a los alimentos |  |  |  |  |  |  |  |  |  |  |  |  |
| Fiebre del heno (rinitis alérgica) |  |  |  |  |  |  |  |  |  |  |  |  |
| Ansiedad |  |  |  |  |  |  |  |  |  |  |  |  |
| Desorden bipolar |  |  |  |  |  |  |  |  |  |  |  |  |
| Depresión |  |  |  |  |  |  |  |  |  |  |  |  |
| Desorden alimenticio |  |  |  |  |  |  |  |  |  |  |  |  |
| Esquizofrenia |  |  |  |  |  |  |  |  |  |  |  |  |
| Alcoholismo u otro abuso de sustancias |  |  |  |  |  |  |  |  |  |  |  |  |
| Trastorno de déficit de atención e hiperactividad (TDAH) |  |  |  |  |  |  |  |  |  |  |  |  |
| Trastorno del espectro autista (TEA)  ) |  |  |  |  |  |  |  |  |  |  |  |  |
| Discapacidad de aprendizaje (p. ej., dislexia) |  |  |  |  |  |  |  |  |  |  |  |  |
| Discapacidad intelectual (p. ej., retraso mental) |  |  |  |  |  |  |  |  |  |  |  |  |
| Epilepsia o trastorno convulsivo |  |  |  |  |  |  |  |  |  |  |  |  |
| Colesterol alto (hiperlipidemia) |  |  |  |  |  |  |  |  |  |  |  |  |
| Hipertensión / presión arterial alta |  |  |  |  |  |  |  |  |  |  |  |  |
| Diabetes tipo 1 |  |  |  |  |  |  |  |  |  |  |  |  |
| Diabetes tipo 2 |  |  |  |  |  |  |  |  |  |  |  |  |
| Trastorno de la tiroides |  |  |  |  |  |  |  |  |  |  |  |  |
| Enfermedad de la arteria coronaria (p. ej., ataque cardíaco, angina) |  |  |  |  |  |  |  |  |  |  |  |  |
| Cualquier otra enfermedad cardíaca |  |  |  |  |  |  |  |  |  |  |  |  |
| Derrame cerebral |  |  |  |  |  |  |  |  |  |  |  |  |
| Cáncer de mama |  |  |  |  |  |  |  |  |  |  |  |  |
| Cáncer de colon |  |  |  |  |  |  |  |  |  |  |  |  |
| Otro cáncer (especifique el tipo) |  |  |  |  |  |  |  |  |  |  |  |  |
| Fallecido |  |  |  |  |  |  |  |  |  |  |  |  |

# **ECHO Income, Assistance, Financial Strain Questions**

Las siguientes preguntas son sobre los ingresos de su familia y cualquier asistencia financiera que recibe.

**Section 1: Income**
Por favor, piense en el **total de los ingresos combinados de su hogar** durante el último año calendario para todos los miembros de su hogar.

**41**. ¿Cuántos miembros del hogar son mantenidos con el total de ingresos combinados de su hogar?

____________(Number)
 ❑ No sé
 ❑ Prefiero no responder

**42**. ¿Cuántas de esas personas son niños? Incluya a cualquier persona menor de 18 años o cualquier persona mayor de 18 años y en la escuela secundaria.

____________(Number)
 ❑ No sé
 ❑ Prefiero no responder

**43.** En cualquier momento DURANTE LOS ÚLTIMOS 12 MESES, incluso durante solo un mes, ¿alguien en su hogar primario recibió ingresos de alguna de las siguientes fuentes? El hogar primario incluye a todos los niños, parientes, esposo/pareja significativa o amigos que viven en su hogar y pasan ***al menos dos noches a la semana en la casa***. Por favor seleccione todas las respuestas válidas.

❑ Sueldo, salarios, comisiones, bonos o propinas de todos los trabajos

❑Ingresos del trabajo por cuenta propia de negocios propios no agrícolas o empresas agrícolas, incluidas las empresas y asociaciones

❑Intereses, dividendos, ingresos netos por alquileres, ingresos por regalías o ingresos provenientes de propiedades y fideicomisos

❑ Seguridad social o jubilación ferroviaria

❑ Seguridad de Ingreso Suplementario (SSI)

❑ Cualquier asistencia pública o pagos de asistencia social de la oficina de bienestar estatal o local

❑ Pensión de jubilación, sobreviviente o de invalidez

❑ Cualquier otra fuente de ingresos recibida regularmente, como pagos de Veteranos (VA), compensación por desempleo, manutención de los hijos o pensión alimenticia. NO incluya pagos a suma al contado, como el dinero de una herencia o la venta de una casa

**44**. En cualquier momento DURANTE EL último año calendario, incluso durante un mes, ¿alguien en su hogar recibió:

| Asistencia en efectivo de un programa de bienestar del gobierno (por ejemplo, Asistencia Temporal para Familias Necesitadas [TANF], anteriormente conocida como Ayuda a Familias con Hijos Dependientes) | ❑ Sí  ❑ No  ❑ No sé  ❑ Prefiero no responder |
| --- | --- |
| Estampillas para comida o beneficios del Programa de Asistencia de Nutrición Suplementaria (SNAP) | ❑ Sí  ❑ No  ❑ No sé  ❑ Prefiero no responder |
| Beneficios del programa Mujer, bebés y niños (WIC) | ❑ Sí  ❑ No  ❑ No sé  ❑ Prefiero no responder |

**45.** ¿Cuál de las siguientes categorías representa mejor el **total** de ingresos combinados de su hogar durante el *último año calendario*? Esto incluye todo el dinero ganado por los miembros primarios del hogar que contribuyen a los gastos del hogar (por ejemplo, pareja/esposo, padres). Asegúrese de incluir el total de sueldos, salarios, ingresos de autoempleo después de gastos, asistencia gubernamental de cualquier tipo, intereses y dividendos, etc., antes de impuestos.

❑ MENOS DE $4,999
❑$5,000-$9,999

❑$10,000-$19,999
 ❑$20,000-$29,999
 ❑$30,000-$39,999
 ❑$40,000-$49,999
 ❑$50,000-$74,999
 ❑$75,000-$99,999
 ❑$100,000-$199,999
 ❑$200,000 O MÁS
 ❑ No sé
 ❑ Prefiero no responder

**46.** Durante el último año calendario, ¿con qué frecuencia pospone la compra de algo que necesita, como alimentos, ropa, atención médica o vivienda, porque no tiene dinero? Diría...

❑Nunca

❑ Raramente
❑Ocasionalmente
❑Frecuentemente
❑Todo el tiempo
❑ Prefiero no responder

**47**. Durante el último año calendario, ¿cuánta dificultad ha tenido al pagar las cuentas? Diría...

❑ Ninguna dificultad en absoluto

❑ Un poco de dificultad

❑ Alguna dificultad

❑ Bastante dificultad
❑ Una gran dificultad
❑ Prefiero no responder

**48**. Pensando en el final de cada mes durante el último año calendario, generalmente termino con…
❑ Más que suficiente dinero sobrante

❑ Algo de dinero sobrante

❑ Solo lo suficiente para llegar a fin de mes
❑ No suficiente para llegar a fin de mes
❑ Prefiero no responder

**49**. ¿Ha reservado fondos de emergencia que cubrirían sus gastos durante 3 meses, en caso de enfermedad, pérdida de trabajo, recesión económica u otras emergencias?

❑ Sí
 ❑ No
 ❑ Prefiero no responder

**MOTHER’S FAMILY PLANNING AND WEIGHT LOSS**

**50.** ¿Tiene la intención de tener un hijo adicional dentro de los próximos 12 meses?

1 ❑ Sí
 0 ❑ No

**51**. ¿Tiene la intención de volver a un método anticonceptivo?

1 ❑ Sí
 0 ❑ No

**52.** ¿Cuánto peso aumento durante su embarazo?

1 ❑ Menos de10 lbs.

2 ❑ 10-20 lbs.

3 ❑ 20-30 lbs.

4 ❑ Más de 30 lbs.

9 ❑ No sé

**53.** ¿Cuánto le preocupa perder el peso del embarazo?

**BABY’S HEALTH**

**54.**, Cuando su bebé fue dado de alta del hospital después que usted dio a luz, ¿tuvo su bebé más de un 10% de disminución de peso?

0 ❑ No
1 ❑ Sí
9 ❑ No sé

**55.** ¿Su bebé fue al médico para una visita de una semana después del nacimiento?
 0 ❑ No…**SKIP** to Question 57
 1 ❑ Sí

**56.** ¿Dijo el médico de su bebé que su bebé estaba subiendo de peso regularmente y que el crecimiento iba de acuerdo con el plan?
1 ❑ Sí
0 ❑ No
9 ❑ No sé

**57**. ¿Recibió su bebé antibióticos desde su nacimiento?
0 ❑ No
1 ❑ Sí, un vez
9 ❑ Sí, más de una vez

**BREASTFEEDING**

**Questions 58-62 Breastfeeding and Infant Feeding**

Fein SB, Labiner-Wolfe J, Shealy KR, Li R, Chen J, Grummer-Strawn LM: **Infant Feeding Practices Study II: study methods**. *Pediatrics* 2008, **122 Suppl 2**:S28-35.

**GLOBAL HEALTH QUESTIONS**

**Questions 63-72 PROMIS® Scale v1.2 – Global Health**

Cella, D., Riley, W., Stone, A., Rothrock, N., Reeve, B., Yount, S., Amtmann, D., Bode, R., Buysse, D. J., Choi, S. W., Cook, K. F., DeVellis, R., DeWalt, D., Fries, J. F., Gershon, R., Hahn, E., Pilkonis, P., Revicki, D., Rose, M., Weinfurt, K., & Hays, R. D. on behalf of the PROMIS Cooperative Group. (2010). Initial item banks and first wave testing of the Patient–Reported Outcomes Measurement Information System (PROMIS) network: 2005–2008. *Journal of Clinical Epidemiology, 63*(11), 1179-94.

**PERCEIVED IMMIGRATION POLICY EFFECTS**

**73. Las siguientes preguntas le preguntarán con qué frecuencia ha vivido ciertas ocurrencias basadas en su origen étnico o estado migratorio. Por favor dígame una respuesta para cada pregunta de acuerdo a sus experiencias.**

|  | Nunca | Un poco | A veces | A menudo | Siempre |
| --- | --- | --- | --- | --- | --- |
| **A**. ¿Fue tratada injustamente en un restaurante o tienda? |  |  |  |  |  |
| **B**. ¿Fue callada por otros o sintió que su opinión no importaba? |  |  |  |  |  |
| **C.** ¿Actuaron los demás como si tuvieran el derecho de tratarla injustamente o mal? |  |  |  |  |  |
| **D**. ¿Fue tratada como un criminal basado en quién es usted? |  |  |  |  |  |
| **E**. ¿La trataron mal por no hablar inglés? |  |  |  |  |  |
| **F**. ¿Se sintió ignorada cuando buscaba ayuda? |  |  |  |  |  |
| **G**. ¿Fue humillada por ser quién es? |  |  |  |  |  |
| **H**. ¿Sintió que estaba siendo explotada o estaban tomando ventaja de usted en el trabajo? |  |  |  |  |  |
| **I.** ¿Sintió que no tenía libertad y necesitaba quedarse en casa? |  |  |  |  |  |
| **J.** ¿Evitó ciertos lugares como parques y vecindarios porque no se sentía segura? |  |  |  |  |  |
| **K**. ¿Temía ser deportada o detenida? |  |  |  |  |  |
| **L**. ¿Se sintió insegura al salir de tu casa? |  |  |  |  |  |
| **M.** ¿Sintió que no tenía derechos? |  |  |  |  |  |
| **N.** ¿Se preocupaba por el impacto de estas políticas en tu familia? |  |  |  |  |  |
| **O**. ¿Temía que usted o un miembro de su familia fuera denunciado a los funcionarios de inmigración? |  |  |  |  |  |
| **P**. ¿Se preocupaba por la separación de la familia debido a la deportación? |  |  |  |  |  |

**74. ¿Tiene otros hijos?** ❑ Sí.. Continue to chart
 ❑ No.. Skip to Question #74

|  | Nunca | Un poco | A veces | A menudo | Siempre |
| --- | --- | --- | --- | --- | --- |
| **A**. ¿Han estado estresados ​​sus hijos de que miembros de la familia sean deportados o detenidos? |  |  |  |  |  |
| **B**. ¿Se han sentido inseguros sus hijos debido a las políticas de inmigración? |  |  |  |  |  |
| **C.** ¿Le ha preocupado que sus hijos hayan tenido problemas emocionales debido a las políticas de inmigración? |  |  |  |  |  |
| **D**. ¿Han temido sus hijos a las autoridades debido a las políticas de inmigración? |  |  |  |  |  |
| **E**. ¿Han tenido sus hijos dificultades para concentrarse en la escuela debido a las políticas de inmigración? |  |  |  |  |  |
| **F**. ¿Han tratado a sus hijos de manera diferente en la escuela debido a políticas de inmigración o actitudes negativas hacia los inmigrantes? |  |  |  |  |  |
| **G**. ¿Le han negado a sus hijos otros servicios debido a políticas de inmigración o actitudes negativas hacia los inmigrantes? |  |  |  |  |  |

**SOCIAL SUPPORT QUESTIONS**

**Question 75 Emotional Support, Instrumental Support, and Informational Support PROMIS® Scale**
Cella, D., Riley, W., Stone, A., Rothrock, N., Reeve, B., Yount, S., Amtmann, D., Bode, R., Buysse, D. J., Choi, S. W., Cook, K. F., DeVellis, R., DeWalt, D., Fries, J. F., Gershon, R., Hahn, E., Pilkonis, P., Revicki, D., Rose, M., Weinfurt, K., & Hays, R. D. on behalf of the PROMIS Cooperative Group. (2010). Initial item banks and first wave testing of the Patient–Reported Outcomes Measurement Information System (PROMIS) network: 2005–2008. *Journal of Clinical Epidemiology, 63*(11), 1179-94.

**POSTPARTUM DISTRESS MEASURE**

**Questions 76-84 Postpartum Distress Measure**
 Allison KC, Wenzel A, Kleiman K, Sarwer DB: **Development of a brief measure of postpartum distress**. *J Womens Health (Larchmt)* 2011, **20**(4):617-623
