# Supplementary material for: Study Design, Protocol and Profile of the Maternal And Developmental Risks from Environmental and Social Stressors (MADRES) Pregnancy Cohort: a Prospective Cohort Study in Predominantly Low-Income Hispanic Women in Urban Los Angeles
Source: BMC Pregnancy Childbirth. 2019 May 30;19:189. doi: 10.1186/s12884-019-2330-7 (PMC6543670; doi:10.1186/s12884-019-2330-7)
Supplement: Supplementary file 24 — Three Month Post Birth Questionnaire_Spanish. Spanish questionnaire administered 3 months after child participant is born. (DOCX 160 kb) [file 12884_2019_2330_MOESM24_ESM.docx]

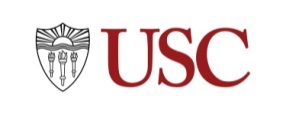
**MADRES Study: Three Month Questionnaire**

**Today’s Date:** _____________________ **Interviewer Name:** ____________________

**Instrucciones:** Gracias por aceptar participar en el estudio MADRES. Durante esta entrevista le hare preguntas sobre usted y su bebe. Por favor responda a todas las preguntas de este cuestionario, aún si no está completamente segura de la respuesta. Le aseguramos que sus respuestas serán confidenciales**.** Por favor, con toda confianza, siéntase libre de interrumpirme y hacerme cualquier pregunta que tenga.

**INFORMACION DE CONTACTO**

**1. Nombre:** ________________ __________________ __________________ ____________________

Nombre 2do Nombre Apellido 1 Apellido 2

**2. Otros nombres que haya usado** (ej. nombre de soltera) ­­­­­­­­­­­­­­­­­­­­­­­­­:­___________________________

**3**. **Fecha de nacimiento:** _**______/_______/_______**

Mes Día Año

**4**. **Nombre del Bebé:** __________________ ________________ __________________ _________________

Nombre 2do Nombre Apellido 1 Apellido 2

**5. Fecha de nacimiento del Bebé:** **_______/_______/_______**

Mes Día Año

**6**. **Género del Bebé:** □₁ Femenino □₂ Masculino

**7. ¿Cuál es su número de celular?** ____________________________

□₀ No tengo teléfono celular **(Skip to question #9)**

**8.** **¿Es un celular pre pagado o es un número fijo de celular?**

□₀ Pre pagado

□₁ Número fijo

**9.** **¿Cuál es su dirección? (la dirección donde usted pasa la mayoría del tiempo):**

Dirección: ________________________________________________________________________

Ciudad: _____________________Estado: ________________Código Postal: ___________________

**9A.** **If moved…¿Cuándo se mudó a su nueva dirección?** _______________________

**10. Por favor dígame los nombres de otros adultos que viven con usted:**

Adult#1 Nombre: ______________________Apellido: ______________________2do Nombre: ______________

Relación: ___________________ Número de Celular: ______________________

Adult#2 Nombre: ______________________Apellido: ______________________2do Nombre: ______________

Relación: ___________________ Número de Celular: ______________________

Adult#3 Nombre: ______________________Apellido: ______________________2do Nombre: ______________

Relación: ___________________ Número de Celular: ______________________

**11. ¿Cuál es el número de teléfono para el domicilio dado en la Pregunta 9?**______________________

□₀ No tengo teléfono de casa

**12. ¿Vive en más de una casa?**

□₁ Sí... *Complete questions 13A, 13B and 13C* □₀No…  *Go to question #14*

**13A. ¿Cuál es la dirección de su segundo domicilio?**

Dirección: _________________________________________________________________________

Ciudad: ______________________Estado: ________________ Código Postal: __________________

**13A2.** **If moved…¿Cuándo se mudó a su nuevo segundo domicilio?** _______________________

**13B. ¿Cuál es el número de teléfono para el domicilio dado en la Pregunta 13A?** _____________________

□₀ No tengo teléfono de casa

**13C. ¿Cuánto tiempo pasa usted en la dirección dada en la pregunta 13A?**

 1%-25% del tiempo

 26%-50% del tiempo

**14. A. ¿Cuál es su correo electrónico?** _________________________ 0 ❑No tengo correo electrónico

**B. ¿Cuál es su nombre de usuario en Facebook?** ___________________________0 ❑No tengo Facebook

**C. ¿Cuál es su nombre de usuario en Twitter?** @___________________________0 ❑No tengo Twitter

**D. ¿Cuál es su nombre de contacto para Instagram?** ____________________0 ❑No tengo Instagram

**15. A. ¿Cómo prefiere ser contactada?**

 Teléfono

 Correo electrónico

 Texto

 Otro: ________________

**B. ¿Cuáles son los mejores días para contactarla?**

 lunes

 martes

 miércoles

 jueves

 viernes

 sábado

 domingo

**C. ¿Cuáles son las mejores horas para contactarla (lunes)?**

 En la añana (8am-12pm)

 En la tarde (12pm-5pm)

 En la noche (5pm-8pm)

 Otro: ______________

**D. ¿Cuáles son las mejores horas para contactarla (martes)?**

 Mañana (8am-12pm)

 Tarde (12pm-5pm)

 Noche (5pm-8pm)

 Otro: ______________

**E. ¿Cuáles son las mejores horas para contactarla (miércoles)?**

 Mañana (8am-12pm)

 Tarde (12pm-5pm)

 Noche (5pm-8pm)

 Otro: ______________

**F. ¿Cuáles son las mejores horas para contactarla (jueves)?**

 Mañana (8am-12pm)

 Tarde (12pm-5pm)

 Noche (5pm-8pm)

 Otro: ______________

**G. ¿Cuáles son las mejores horas para contactarla (viernes)?**

 Mañana (8am-12pm)

 Tarde (12pm-5pm)

 Noche (5pm-8pm)

 Otro: ______________

**H. ¿Cuáles son las mejores horas para contactarla (sábado)?**

 Mañana (8am-12pm)

 Tarde (12pm-5pm)

 Noche (5pm-8pm)

 Otro: ______________

**I . ¿Cuáles son las mejores horas para contactarla (domingo)?**

 Mañana (8am-12pm)

 Tarde (12pm-5pm)

 Noche (5pm-8pm)

 Otro: ______________

**16.** **¿Cómo se llama el papa del bebé?** □ No sé

_________________ _______________ ____________________ ____________________

Nombre 2do Nombre Apellido 1 Apellido 2

**17A.** **¿Tiene usted esposo o pareja?** 0 ❑ No…*Go to Question* 18 1 ❑ Sí

**17B. ¿Cómo se llama su esposo/pareja?**

_________________ _______________ ____________________ ____________________

Nombre 2do Nombre Apellido 1 Apellido 2

**18.** **Para poder localizarla en caso de que se mude o cambie su número de teléfono, ¿nos puede dar la información de su madre y tres amigos o familiares que no vivan con usted que nos podrían dar su información nueva?**

INFORMACION DE SU MADRE

Nombre: ______________________Apellido: ______________________2do Nombre: ______________

Dirección: _________________________________________________________________________

Ciudad: ______________________Estado: ________________ Código Postal: __________________

Número de Celular: ______________________ Número de teléfono de Casa: ______________________

NOK#1

Nombre: ______________________Apellido: ______________________2do Nombre: ______________

Relación: ___________________Correo Electrónico: ____________________________

Número de Celular: ______________________Número de teléfono de Casa: ______________________

NOK#2

Nombre: ______________________Apellido: ______________________2do Nombre: ______________

Relación: ___________________Correo Electrónico: ____________________________

Número de Celular: ______________________Número de teléfono de Casa: ______________________

NOK#3

Nombre: ______________________Apellido: ______________________2do Nombre: ______________

Relación: ___________________Correo Electrónico: ____________________________

Número de Celular: ______________________Número de teléfono de Casa: ______________________

**DIRECCIÓN DE ENVIO**

**19. ¿Tienes una dirección postal o postal diferente a la dirección de su domicilio?**

0 ❑ No

1 ❑ Sí… ¿cual es su dirección postal?

Dirección: _________________________________________________________________________

Ciudad: ______________________Estado: ________________ Código Postal: __________________

**HISTORIAL CL**Í**NICA DEL BEBÉ**

**20A.** ¿Su nuevo bebé ha sido visto por un médico, una enfermera u otro profesional de la salud para un chequeo de rutina?

0 ❑ No…**SKIP** to Question 21

1 ❑ Sí

**20B.** ¿Cuántas veces ha sido visto su bebé por un médico, una enfermera u otro profesional de la salud para un chequeo de rutina?

______________VECES

**21.** ¿Fue su bebé visto por un médico, una enfermera u otro profesional de la salud para su chequeo de rutina una semana después de que él o ella nació?

0 ❑ No

1 ❑ Sí

**22.** ¿Su bebé ha ido tantas veces como usted ha querido a los chequeos de rutina?

0 ❑ No

1 ❑ Sí

**23**. ¿Alguna de estas cosas evito que su bebé tuviera un chequeo de salud de rutina? **Select all that apply**

1 ❑ No tuve suficiente dinero ni seguro médico para pagar por ello

2 ❑ No tenía manera para llevar a mi hijo/a a la clínica u oficina

3 ❑ No tenía alguien para cuidar de mis otros hijos

4 ❑ No pude conseguir una cita

5 ❑ Mi bebé estaba muy enfermo para ir al cuidado de rutina

6 ❑ Otros: Por favor, explique:____________________

7 ❑ NA (No se aplica)

**24.** ¿Ha tenido su bebe alguna vacuna de niño sano u otras vacunas en el tiempo desde que nació su bebé? No cuente inyecciones o vacunas administradas en el hospital inmediatamente después del nacimiento.

0 ❑ No

1 ❑ Sí

**25.** ¿Su hijo/a ha sido vacunado con la vacuna Tdap (tos ferina o la vacuna contra la tos ferina)?

0 ❑ No

1 ❑ Sí

9 ❑ No sé

**26.** ¿Su hijo/a ha recibido la vacuna contra la gripe o la vacuna nasal contra la gripe?

0 ❑ No

1 ❑ Vacuna contra la gripe

2❑ FluMist nasal

3 ❑ Ambas

9 ❑ No sé

**27A.** ¿Tiene su bebé un pediatra o médico de cabecera?

□₀ No… *Continúe a la Pregunta 26B.* □₁ Sí…Por favor provea la información del pediatra.

Nombre del doctor: _________________________________________________________________

Nombre de la clínica: ____________________________________________________________________

Dirección de la clínica: ________________________________________________________________­­___

Ciudad: _____________________________Estado: ___________________Código postal:_____________

Número de teléfono: ________________________________________________________________­­__

Fechas: De_____________________*(mes/año*) a _________________________*(mes/año*)

**27B**. **¿**Ha tenido su bebé previamente otros pediatras o médicos de cabecera?

□₀ No… *Continúe a la Pregunta 27* □₁ Sí…Por favor provea la información del pediatra.

**Pediatra Previo #1:**

Nombre del doctor: _________________________________________________________________

Nombre de la clínica: ____________________________________________________________________

Dirección de la clínica: ________________________________________________________________­­___

Ciudad: _____________________________Estado: ___________________Código postal:_____________

Número de teléfono: ________________________________________________________________­­__

Fechas: De_____________________*(mes/año*) a _________________________*(mes/año*)

**Pediatra Previo #2:**

Nombre del doctor: _________________________________________________________________

Nombre de la clínica: ____________________________________________________________________

Dirección de la clínica: ________________________________________________________________­­___

Ciudad: _____________________________Estado: ___________________Código postal:_____________

Número de teléfono: ________________________________________________________________­­__

Fechas: De_____________________*(mes/año*) a _________________________*(mes/año*)

**EVALUACION DEL ESTRES**

**Questions 28-37 Perceived Stress Scale**

Cohen S, Kamarck T, Mermelstein R: **A global measure of perceived stress**. *J Health Soc Behav* 1983, **24**(4):385-396.

**MEDIDA DE ANGUSTIA POSTPARTO**

**Questions 38-46 Postpartum Distress Measure**
 Allison KC, Wenzel A, Kleiman K, Sarwer DB: **Development of a brief measure of postpartum distress**. *J Womens Health (Larchmt)* 2011, **20**(4):617-623.

**PESO DE LA MADRE**

**47.** ¿Sabe usted su peso actual o la mejor estimación de su peso actual?

9 ❑ No sé

1 ❑ Sí…

**A.** ¿Cuál es su peso actual o su mejor estimación de su peso actual (en libras)?

_______ lbs

**48.** ¿Cuánto peso aumentó durante su embarazo?

1 ❑ Menos de 10 libras

2 ❑ 10-20 libras

3 ❑ 20-30 libras

4 ❑ Más de 30 libras

9 ❑ No sé

**49.** ¿Ha perdido algo de peso del embarazo?

0 ❑ No (*Skip to Quetsion #51*)

1 ❑ Sí (*Continue*)

**50.** ¿Ha vuelto a su peso de antes del embarazo?

0 ❑ No

1 ❑ Sí

**51.** ¿Cuánto le preocupa perder el peso del embarazo?

0 ❑ N/A He perdido todo el peso del embarazo

1 ❑ No está preocupada en absoluto

2 ❑ No está muy preocupada

3 ❑ Está un poco preocupada

4 ❑ Está muy preocupada

**HISTORIA OCUPACIONAL**

**52**. ¿Cuál es su actual estado de empleo? [SELECT ALL THAT APPLY]

1 ❑ Ama de casa

2 ❑ Estudiante

3 ❑ Empleada

4 ❑ En permiso médico temporal

5 ❑ Desempleada

6 ❑ Otro: (Explique) ____________________

**53.** ¿Ha estado trabajando **desde que nació su bebé, el/en (dar fecha)**?

0 ❑ No (**SKIP TO #58**)

1 ❑ Sí

**54.** ¿Qué edad tenía su bebé cuando regresó al trabajo?

0 ❑ Menos de 1 semana de edad

1 ❑ 1-6 semanas de edad

2 ❑ 6-12 semanas de edad

3 ❑ Más de 12 semanas de edad

**55.** ¿Cuántas horas a la semana trabaja?

1 ❑ Menos de 10 horas/semana

2 ❑ 10-20 horas/semana

3 ❑ 21-30 horas/semana

4 ❑ 31-40 horas/semana

5 ❑ Más de 40 horas/semana

**56**. Durante una semana de trabajo regular, ¿cuantos días a la semana le toca viajar de ida y de regreso del trabajo?

0 ❑ 0 día

1 ❑ 1 día

2 ❑ 2 días

3 ❑ 3 días

4 ❑ 4 días

5 ❑ 5 días

6 ❑ 6 días

7 ❑ 7 días

**57.** Piensa acerca de tu viaje típico de ida al trabajo, ¿qué formas de transporte utilizas y por cuánto tiempo? (**Select all that apply.)**

|  | **1-10**  **Minutos** | **11-20**  **Minutos** | **21-30**  **Minutos** | **31-59**  **Minutos** | **60-90**  **Minutos** | **91-120**  **Minutos** | **2 Horas o mas** | **N/A** |
| --- | --- | --- | --- | --- | --- | --- | --- | --- |
| Coche/carro |  |  |  |  |  |  |  |  |
| Bus o  Tranvía |  |  |  |  |  |  |  |  |
| Tren o Metro |  |  |  |  |  |  |  |  |
| Motocicleta |  |  |  |  |  |  |  |  |
| Bicicleta |  |  |  |  |  |  |  |  |
| A pie |  |  |  |  |  |  |  |  |

**PREGUNTAS SOBRE EL USO DEL CIGARRILLO**

**58.** Sin incluir cigarrillos eléctricos, ¿Ha fumado usted cigarrillos, cigarros o pipas alguna vez en su vida?

0 ❑ No (**SKIP TO #61**)

1 ❑ Sí

**59.** **Desde que nació su bebé**, **el/en (dar fecha),** sin incluir cigarrillos eléctricos, ¿ha fumado usted cigarrillos, cigarros o pipas?

0 ❑ No (**SKIP TO # 61**)

1 ❑ Sí

**60.** ¿Ha fumado usted cigarrillos, cigarros o pipas en los últimos 5 días?

0 ❑ No:

**A.** Si NO fuma actualmente**,** ¿cuándo dejo de fumar? [**SELECT ONE**]

1 ❑ Hace menos de 2 semanas

2 ❑ 2 a 4 semanas atrás

3 ❑ Más de 4 semanas atrás

4 ❑ No recuerdo

**B.** Si NO fuma actualmente**,** ¿Cuántos cigarrillos fumaba al día?

1 ❑ 1- 5 3 ❑ 11-20

2 ❑ 6-10 4 ❑ Más de 20

1 ❑ Sí:

**A.** ¿Cuántos cigarrillos fuma al día?

1 ❑ 1- 5 3 ❑ 11-20

2 ❑ 6-10 4 ❑ Más de 20

**61.** ¿Ha fumado usted cigarrillos electrónicos o algún otro sistema electrónico de administración de nicotina (e-hookah, e-cigars, etc.)?

0 ❑ No (**SKIP TO #64**)

1 ❑ Sí

**62.** **Desde que nació su bebé**, ¿Ha fumado usted cigarrillos electrónicos o algún otro sistema electrónico de administración de nicotina (e-hookah, e-cigars, etc.)?

0 ❑ No **(SKIP TO #64)**

1 ❑ Sí

**63.** ¿Ha fumado usted cigarrillos electrónicos o algún otro sistema electrónico de administración de nicotina (e-hookah, e-cigars, etc.) en los últimos 5 días?

0 ❑ No:

**A.** Si NO fuma actualmente**,** ¿cuándo dejo de fumar? [**SELECT ONE**]

1 ❑ Hace menos de 2 semanas

2 ❑ 2 a 4 semanas atrás

3 ❑ Más de 4 semanas atrás

4 ❑ No recuerdo

**B.** Si NO fuma actualmente**,** ¿Qué tan seguido fumaba cigarrillos electrónicos o algún otro sistema electrónico de administración de nicotina (e-hookah, e-cigars, etc.)?

1❑ Todos los días

2❑ Cada dos o tres días

3❑ Una vez a la semana

4❑ Aproximadamente una vez al mes

5❑ Cada cuantos meses

1 ❑ Sí:

**A.** ¿Qué tan seguido fuma cigarrillos electrónicos o algún otro sistema electrónico de administración de nicotina (e-hookah, e-cigars, etc.)?

1❑ Todos los días

2❑ Cada dos o tres días

3❑ Una vez a la semana

4❑ Aproximadamente una vez al mes

5❑ Cada cuantos meses

**64. Desde que nació su bebé**, **el/en (dar fecha),** sin incluir cigarrillos electrónicos, ¿Alguna otra persona que vive en su casa ha fumado cigarrillos, cigarros o pipas dentro de la casa?

0 ❑ No (**SKIP TO #67)**

1 ❑ Sí

**65.** **Desde que nació su bebé**, **el/en (dar fecha),** ¿Quién más en su casa ha fumado cigarrillos, cigarros o pipas?  **(SELECT ALL THAT APPLY)**

1 ❑ Padre del bebe

2 ❑ Otras personas

**66. Desde que nació su bebé**, **el/en (dar fecha),** sin incluirse a usted misma ¿Cuántas personas viviendo en su casa fuman cigarrillos, cigarros o pipas?

1 ❑ 1

2 ❑ 2

3 ❑ 3

4 ❑ 4 o más

**67.** **Desde que nació su bebé**, **el/en (dar fecha),** *en un promedio*, ¿Cuántas horas al día está expuesta usted al humo de cigarrillos, cigarros o pipas fumados por otras personas?

1 ❑ 0-1 hora

2 ❑ 1-2 horas

4 ❑ 3-4 horas

5 ❑ Más de 4 horas

3 ❑ 2-3 horas

**68.** **Desde que nació su bebé**, **el/en (dar fecha),** *en un promedio*, ¿Cuántas horas al día está expuesto/a **su** **bebé** al humo de cigarrillos, cigarros o pipas fumados por otras personas?

1 ❑ 0-1 hora

2 ❑ 1-2 horas

4 ❑ 3-4 horas

5 ❑ Más de 4 horas

3 ❑ 2-3 horas

**AMAMANTAMIENTO**

**Questions 69-82 Infant Feeding Practices**

Fein SB, Labiner-Wolfe J, Shealy KR, Li R, Chen J, Grummer-Strawn LM: **Infant Feeding Practices Study II: study methods**. *Pediatrics* 2008, **122 Suppl 2**:S28-35.

**CUIDADO DEL BEBÉ**

**83.**¿Qué tipo de arreglo de alojamiento / custodia tiene usted con su hijo/a?

_1_❑ Su hijo/a vive con ambos padres en la misma casa.

_2_❑ Su hijo/a vive con ambos padres en casas separadas, dividiendo el tiempo casi por igual.

_3_❑ Su hijo/a vive con usted la mayor parte del tiempo en su casa.

_4_❑ Su hijo/a vive con el otro padre la mayor parte del tiempo en una casa diferente a la suya.

_5_❑ Su hijo/a vive en la misma casa todo momento, pero los padres entran y salen.

_6_❑ Otro (Por favor especifique): _________________________________________________________

**84.** **Desde que nació su bebé,** ¿su bebé ha pasado tiempo en una guardería o sido cuidado por alguien que no sea usted?

□₀ No… (**Skip to #89**)

□₁ Sí

**85.** La mayor parte del tiempo, ¿en qué lugar fue cuidado su bebé?

_1_❑ En su propia casa

_2_❑ En la casa de otra persona

_3_❑ En un programa formal de guardería

**86**. Durante una semana típica, ¿cuántos días por semana cuidan estas personas / programas / centros a su bebé? (Incluyendo tardes, noches, y fines de semana)

_1_❑ 5 o más días

_2_❑ 3 - 4 días

_3_❑ 1- 2 días

_4_❑ Menos de 1 día

**87**. En los días en que su bebé fue cuidado por alguien más en una semana típica, ¿cuántas horas por día pasa su bebé con estas personas / programas / centros?

_1_❑ 5 o más horas

_2_❑ 3 - 4 horas

_3_❑ 1- 2 horas

_4_❑ Menos de 1 hora

**88.** Además de usted, ¿cuáles son sus actuales servicios de guardería (Por favor, díganos todos los que son aplicables)?

|  | **¿Quién cuida de su bebé?** | **¿Cuántos días a la semana?** | **¿Cuántas horas al día?** | **¿Dónde cuidan de su bebé? (e.g., casa de la abuela, servicio de guardería)** | **¿Cuál es la dirección?** |
| --- | --- | --- | --- | --- | --- |
| **Arreglo de cuidado de niños 1** |  |  |  |  |  |
| **Arreglo de cuidado de niños 2** |  |  |  |  |  |
| **Arreglo de cuidado de niños 3** |  |  |  |  |  |

**SALUD DEL BEBÉ**

**89.** **¿Ha tenido alguna de las siguientes condiciones desde que nació su bebé?**

|  | | **No** | **Sí, pero no vio médico** | **Sí y vio a un médico** | **Sí, vio un médico y recibió medicina prescrita** |
| --- | --- | --- | --- | --- | --- |
| a. | Diarrea | □_0_ | □_1_ | □_2_ | □_3_ |
| b. | Sangre en el excremento | □_0_ | □_1_ | □_2_ | □_3_ |
| c. | Vomito | □_0_ | □_1_ | □_2_ | □_3_ |
| d. | Toz | □_0_ | □_1_ | □_2_ | □_3_ |
| e. | Alta temperatura (más de 101° F/38° C) | □_0_ | □_1_ | □_2_ | □_3_ |
| f. | Nariz que moquea | □_0_ | □_1_ | □_2_ | □_3_ |
| g. | Resfriado | □_0_ | □_1_ | □_2_ | □_3_ |
| h. | Dolor de oído | □_0_ | □_1_ | □_2_ | □_3_ |
| i. | Descarga del oído (pus no cera) | □_0_ | □_1_ | □_2_ | □_3_ |
| j. | Convulsiones | □_0_ | □_1_ | □_2_ | □_3_ |
| k. | Cólico | □_0_ | □_1_ | □_2_ | □_3_ |
| l. | Inquietud o irritable | □_0_ | □_1_ | □_2_ | □_3_ |
| m. | Reflujo | □_0_ | □_1_ | □_2_ | □_3_ |
| n. | Salpullido | □_0_ | □_1_ | □_2_ | □_3_ |
| o. | Manchas rojas que pican o escamosos en las mejillas, el cuero cabelludo, los codos o las rodillas, como con eczema | □_0_ | □_1_ | □_2_ | □_3_ |
| p. | Una profunda tos seca, acompañada de silbidos o chisporroteo en el pecho, como con infecciones respiratorias como el virus sincitial respiratorio (VSR), bronquitis o neumonía | □_0_ | □_1_ | □_2_ | □_3_ |
| q. | Un accidente / lesión. Por favor describa: | □_0_ | □_1_ | □_2_ | □_3_ |
| r. | Otro. Por favor describa: | □_0_ | □_1_ | □_2_ | □_3_ |

**90A.** ¿Su bebé ha tenido silbidos en el pecho alguna vez en el pasado?

□₀ No…*Skip to Question #91* □₁ Sí

**90B**. ¿ Cuáles son las edades en las que su bebé tuvo silbidos en el pecho? (*Marque todo lo que corresponda*)

□₁ Nacimiento a un mes

□₂ 1 mes a 2 meses

□₃ 2 meses a 3 meses

□₄ 3 meses a 4 meses

**90C**. ¿Cuántos ataques de sibilancias o dificultad para respirar ha tenido su bebé desde que nació?

0 ❑ 1

1 ❑ 2

2 ❑ 3

3 ❑ 4 o más

**91.** ¿Ha recibido su bebé alguno de los siguientes medicamentos desde su nacimiento?

|  | **NO_0_** | **Sí,**  **una vez_1_** | **Sí, más de una vez_2_** |
| --- | --- | --- | --- |
| Antibióticos |  |  |  |
| Otros medicamentos recetados |  |  |  |
| Medicamentos sin receta |  |  |  |

**92.** ¿Ha diagnosticado un médico a su bebé con una alergia a los alimentos?

□₀ No (**Skip** to #96A)

□₁ Sí

**93.** ¿Qué alimento/s es su bebé alérgico a: *(Select all that apply)*

□₁ Cualquier tipo de nueces de árbol...

¿A qué tipos de nueces de árbol es su bebé alérgico? (*Select all that apply*):

□₁ Almendras

□_2_ Nueces de Brasil

□_3_ Anacardos

□_4_ Castañas

□_5_ Avellanas

□_6_ Nueces de macadamia

□_7_ Nueces pecanas

□_8_ Pistachos

□_9_ Nueces

□_2_ Cacahuates

□_3_ Leche

¿A qué tipo de leche es su bebé alérgico? (*Select all that apply* ):

□₁ Leche de vaca

□_2_ Leche materna

□_3_ Leche de cabra

□_4_ Huevos

□_5_ Semilla de sésamo

□_6_ Otro, por favor especifique: ___________________________________

**94.** ¿Ha tenido su hijo/a una reacción alérgica por la cual ha necesitado un medicamento epinefrina inyectable para su alergia a un alimento?

□₀ No

□₁ Sí

**95.** ¿Cuándo fue su bebe diagnosticado con una alergia alimentaria por un doctor?

Edad diagnosticada: ____________ Meses ____________ Semanas

**96A.** ¿Ha llevado a su hijo/a al doctor/cuidado urgente/sala de emergencia por que él/ella tenía un problema que a usted le preocupaba?

□₀ No… *Go to Question #96.* □₁ Sí … *Go to question #95B*

**96B.** ¿Cuántas veces? _________

**96C. Por favor dígame las fechas aproximadas de cada visita al médico / atención de urgencia / a la sala de emergencia y la razón acompañada por la visita.**

|  | ¿Cuáles fueron las fechas aproximadas? |  | ¿Cuál era / eran el problema (s) por las cuales estabas preocupada? |
| --- | --- | --- | --- |
| 1.Mes/Día/Año: | __________________________ |  | __________________________________ |
| 2. Mes/Día/Año: | __________________________ |  | __________________________________ |
| 3. Mes/Día/Año: | __________________________ |  | __________________________________ |
| 4. Mes/Día/Año: | __________________________ |  | __________________________________ |

**96D.** ¿Fue su hijo/a admitido/a al hospital?

□₀No... *Go to Question 90.* □₁ Sí … *Go to question 89d*

**96E.** Por favor describa cada admisión:

|  | Edad del niño/a (semanas) |  | Motivo de admisión |  | Número de noches que el/la niño/a permaneció hospitalizado/a |
| --- | --- | --- | --- | --- | --- |
| 1. |  |  |  |  |  |
| 2. |  |  |  |  |  |
| 3. |  |  |  |  |  |
| 4. |  |  |  |  |  |

**97.** ¿Su bebé tiene algún problema médico grave de largo plazo?

0 ❑ No

1 ❑ Sí

Por favor, explique brevemente: __________________________________________________

**CARACTERISTICAS DEL HOGAR**

******For Administrator Only (Do not ask participant):**

Did participant move since the baby was born (give date)?

0 ❑ No… Ask 100,101, 103, 104-112, 114-115

1 ❑ Yes… Ask 98-115

**98**. **¿Cuál opción describe mejor la casa en la cual reside actualmente la mayor parte del tiempo?** *Seleccione una sola respuesta.*

1 ❑ Una casa (que no está unida a otras casas)

2 ❑ Un edificio de 2-4 departamentos unidos, townhome, condominio, dúplex o triplex

3 ❑ Un edificio de 5-10 departamentos unidos, townhome, condominio, etc.

4 ❑ Un edificio de más de 10 departamentos unidos, townhome, condominio, etc.

5 ❑ Una casa móvil (“mobile home”) o en un tráiler

6 ❑ Otro, por favor explique: ___________________________________________________

**99**. **Aproximadamente, ¿cuándo fue esta vivienda originalmente construida? (cuando se construyó por primera vez, no cuando pudo haber sido remodelada o modificada).** *[Select one]*

1❑ 2000s o más reciente

2❑ 1980s-1990s

3❑ 1960s-1970s

4❑ 1940s-1950s

5❑ Antes de 1940

**100.** **Desde el nacimiento de su bebé, el/en (dar fecha),** ¿Cuáles de las siguientes mascotas ha tenido/tiene dentro de su casa? **(SELECT ALL THAT APPLY)**

1 ❑ No tengo mascotas

2 ❑ Perro(s)

3 ❑ Gato(s)

4 ❑ Otras mascotas (Explique: ____________)

**101.** **Desde el nacimiento de su bebé, el/en (dar fecha),** ¿Ha tenido Usted alguna de las siguientes infestaciones en su casa? **(SELECT ALL THAT APPLY)**

1 ❑ Ratas

2 ❑ Ratones

3 ❑ Cucarachas

4 ❑ Otras infestaciones (Explique:____________)

5 ❑ No sé

6 ❑ Ningún problema con infestaciones

**102**. ¿Tiene en su casa una estufa u horno de GAS?

0 ❑ No

1 ❑ Sí:

**A.** ¿Con que frecuencia usa la estufa u horno mientras que usted está en casa? *Select one.*

1 ❑ Nunca **(SKIP to #103)**

2 ❑ Menos de una vez por semana

3 ❑ 1-3 veces por semana

4 ❑ 4-7 veces por semana

5 ❑ 8-14 veces por semana

6 ❑ Más de 14 veces por semana

**B.** En promedio, ¿Por cuánto tiempo se usa la estufa u horno durante el día mientras que usted está en casa?

1 ❑ Menos de 15 minutos

2 ❑ 15 minutos a menos de 30 minutos

3 ❑ 30 minutos a menos de 1 hora

4 ❑ 1 hora o más

**103.** **Desde el nacimiento de su bebé, el/en (dar fecha),** en promedio, ¿cuántas veces a la semana cocina usted (usando la estufa /horno, no incluyendo el uso del microondas)?

_1_❑ Nunca _3_❑ 4 – 5 veces por semana

_2_❑ 1 – 3 veces por semana _4_❑ Todos los días

**104.** ¿Su casa tiene sistema de calefacción o calentón?

0 ❑ No **(SKIP to #104)**

1 ❑ Sí:

**A.** ¿Cuál es el principal combustible utilizado para calentar la casa? *Select one.*

1 ❑ Gas (podrá ver una llama azul o el piloto encendido dentro de la unidad)

2 ❑ Eléctrico (podrá ver un alambre o metal ardiente dentro de la unidad)

3 ❑ Un tanque de gas (un tanque o cilindro fuera de la casa que se puede llenar de gas)

4 ❑ Leña

5 ❑ Otro, por favor explique: ________________________

9 ❑ No sé cómo se calienta

**105.** ¿Cuál es el principal sistema de calefacción en su casa? *Select one.*

1 ❑ Aire forzado

2 ❑ Unidad eléctrica pegada a la pared

3 ❑ Calentón ubicado en la pared

4 ❑ Calentón ubicado en el piso

5 ❑ Calentador portátil…**¿Qué tipo?**

1 ❑ Gas

2 ❑ Eléctrico

3 ❑ No sé

6 ❑ Otro, por favor explique: ______________________________

9 ❑ No sé cómo se calienta

**106.** ¿Usa usted aire acondicionado en su casa?

0 ❑ No **(SKIP to #109)**

1 ❑ Sí:

**A.** ¿Cuál es el tipo principal de aire acondicionado que se utiliza? *Select one.*

1 ❑ Aire acondicionado de ventana o pared (caja que sale de la ventana o pared)

**a.** ¿Cuántas unidades de ventana/pared tiene usted en su casa?

_1_ ❑ Uno

_2_ ❑ Dos

_3_ ❑ Tres

_4_ ❑ Cuatro o más

_5_ ❑ No sé

2 ❑ Central (escape de aire en las recamaras)

3 ❑ Enfriador de vapor (“swamp cooler”)

9 ❑ No sé qué tipo sea

**107**. **Durante el último mes,** ¿con que frecuencia uso el aire acondicionado estando en casa?

1 ❑ Nunca

2 ❑ Menos de 5 días

3 ❑ 5-15 días

4 ❑ 16-30 días

9 ❑ No sé

**108.** En un día cualquiera, ¿cuánto tiempo uso usted el aire acondicionado en su casa?

_1_ ❑ Nunca

_2_ ❑ Un par de horas al día

_3_ ❑ La mitad del tiempo

_4_ ❑ La mayor parte del tiempo

_5_ ❑ Todo el tiempo

_9_ ❑ No sé

**109.** **Durante el último mes,** ¿usó usted un ventilador de ventana o algún otro ventilador que puso en la ventana o en el ático para enfriar su casa?

0 ❑ No

1 ❑ Sí

**110.** **Desde el nacimiento de su bebé, el/en (dar fecha),** ¿ha habido algún tipo de daño causado por agua o inundación en su casa?

0 ❑ No

1 ❑ Sí:

**A.** ¿Inundó áreas alfombradas?

0 ❑ No

1 ❑ Sí

9 ❑ No sé

**111.** **Desde el nacimiento de su bebé, el/en (dar fecha),**¿se ha formado alguna vez moho en las paredes, techos, o pisos en su casa?

0 ❑ No

1 ❑ Sí:

**A.** ¿Qué cuartos quedaron afectados? *Select all that apply.*

1 ❑ El cuarto donde duerme

2 ❑ Baño (s)

3 ❑ Sótano

4 ❑ Otro

9 ❑ No sé

**112.** ¿Se ha utilizado un humificador o vaporizador en su casa? (Incluyendo el humificador que puede tener dentro el sistema de calefacción.)

0 ❑ No

1 ❑ Sí:

**A.** ¿Qué tipo es? *Select all that apply.*

1 ❑ Viene dentro el sistema de calefacción

2 ❑ Una unidad portátil

**B.** ¿Ha utilizado este aparato para tratar alguna enfermedad respiratoria?

0 ❑No

1 ❑Sí

**C.**  ¿Calienta el aire el humificador o vaporizador?

0 ❑No

1 ❑ Sí

9 ❑ No sé

**113**. ¿Hay alfombra en su casa?

0 ❑ No

1 ❑ Sí:

**A.** ¿En qué cuartos? *Select all that apply.*

1 ❑ Toda la casa (excluyendo la cocina y baño)

2 ❑ Recamara donde duerme

3 ❑ Otras recamara(s)

4 ❑ Otros cuarto(s)

**114.** Recordando un día típico entresemana de la **semana pasada**, aproximadamente cuantas horas (de 24 horas en total) estuvo…

**A.** Afuera: ________________

**B.** Adentro de la casa (Incluyendo la noche/durmiendo):____________________

**115.** En promedio, ¿cuánto tiempo mantuvo las ventanas abiertas durante esta última **semana**?

_1_ ❑ Nunca

_2_ ❑ Un par de horas al día

_3_ ❑ La mitad del tiempo

_4_ ❑ La mayor parte del tiempo

_5_ ❑ Todo el tiempo

_9_ ❑ No

**USO DE PESTICIDAS**

Me gustaría hacerle algunas preguntas sobre pesticidas o insecticidas que se han utilizado en los alrededores de (cualquiera de) la casa (s) en las cuales su bebé ha vivido. Los pesticidas pueden venir en forma de aerosoles, bombas, pastillas de veneno o polvo, tiza, moteles de cucarachas, trampas, o estacas de hormigas.

**116.** **Desde que nació su bebé,** ¿se han utilizado pesticidas o insecticidas en o alrededor de su casa para matar roedores o insectos (por ejemplo, ratones, ratas, hormigas, cucarachas o arañas)?

0 ❑ No… Go to Question #117

_9_ ❑ No sé … Go to Question #117

1 ❑ Sí:

**A.** ¿Cuál fue la forma de este pesticida? (Select all that apply)

1 ❑ Gránulos de veneno

2 ❑ Carnadas o trampas

3 ❑ Aerosoles

4 ❑ Bombas

5 ❑ Veneno en forma de polvo

6 ❑ Otro: _________________________________________________

_9_ ❑ No sé

**B.** ¿Se utilizaron estos pesticidas en el interior o fuera del hogar?

_1_ ❑ En el interior

_2_ ❑ Por afuera

_3_ ❑ Ambos

_9_ ❑ No sé

**C.** Desde que nació su bebé, ¿con qué frecuencia se han aplicado estos pesticidas?

1 ❑ Menos de una vez al mes

2 ❑ Una vez al mes

3 ❑ Cada dos semanas

4 ❑ Cada semana

5 ❑ Más de una vez por semana

**D.** ¿Aplico usted personalmente estos pesticidas?

0 ❑ No

1 ❑ Sí

**117.** Desde que nació su bebé, ¿se han utilizado pesticidas o insecticidas en o alrededor de su casa para matar pulgas, incluyendo tratamientos a los animales domésticos?

0 ❑ No… Go to Question #118

_9_ ❑ No sé … Go to Question #118

1 ❑ Sí:

**A.** ¿Cuál fue la forma de este pesticida? (Select all that apply)

1 ❑ Aerosoles

2 ❑ Bombas

3 ❑ Veneno en forma de polvo

4 ❑ Collar antipulgas

5 ❑ Gotas líquidas

6 ❑ Otro: _________________________________________________

_9_ ❑ No sé

**B.** ¿Se utilizaron estos pesticidas en el interior o fuera del hogar?

_1_ ❑ En el interior

_2_ ❑ Por afuera

_3_ ❑ Ambos

_9_ ❑ No sé

**C.** Desde que nació su bebé, ¿con qué frecuencia se han aplicado estos pesticidas?

1 ❑ Menos de una vez al mes

2 ❑ Una vez al mes

3 ❑ Cada dos semanas

4 ❑ Cada semana

5 ❑ Más de una vez por semana

**D.** ¿Aplico usted personalmente estos pesticidas?

0 ❑ No

1 ❑ Sí

**118.** Desde que nació su bebé, ¿se han utilizado pesticidas, herbicidas, insecticidas en o alrededor de su casa para matar hongos, malas hierbas, o babosas?

0 ❑ No… Go to Question #119

_9_ ❑ No sé … Go to Question #119

1 ❑ Sí:

**A.** ¿Cuál fue la forma de este pesticida? (Select all that apply)

1 ❑ Gránulos de veneno

2 ❑ Carnadas o trampas

3 ❑ Aerosoles

4 ❑ Bombas

5 ❑ Veneno en forma de polvo

6 ❑ Otro: _________________________________________________

_9_ ❑ No sé

**B.** ¿Se utilizaron estos pesticidas en el interior o fuera del hogar?

_1_ ❑ En el interior

_2_ ❑ Por afuera

_3_ ❑ Ambos

_9_ ❑ No sé

**C.** Desde que nació su bebé, ¿con qué frecuencia se han aplicado estos pesticidas?

1 ❑ Menos de una vez al mes

2 ❑ Una vez al mes

3 ❑ Cada dos semanas

4 ❑ Cada semana

5 ❑ Más de una vez por semana

**D.** ¿Aplico usted personalmente estos pesticidas?

0 ❑ No

1 ❑ Sí

**PREGUNTAS SOBRE COMO DUERME**

**A continuación, vamos a preguntarle acerca de sus patrones y hábitos de dormir durante el último mes (30 días). Piense en el último mes (30 días).**

**119. En el último mes,** ¿cuántas horas de sueño consiguió normalmente en una semana típica (el domingo - jueves)?

_1_ ❑ Menos de 4 horas por noche _5_ ❑ 8 horas por noche

_2_ ❑ 5 horas por noche _6_ ❑ 9 horas por noche

_3_ ❑ 6 horas por noche _7_ ❑ Más de 10 horas por noche

_4_ ❑ 7 horas por noche

**120.** **En el último mes,** ¿cuántas horas de sueño consiguió normalmente en una típica noche de fin de semana (viernes a sábado)?

_1_ ❑ Menos de 4 horas por noche _5_ ❑ 8 horas por noche

_2_ ❑ 5 horas por noche _6_ ❑ 9 horas por noche

_3_ ❑ 6 horas por noche _7_ ❑ Más de 10 horas por noche

_4_ ❑ 7 horas por noche

**Questions 121-133 POSTPARTUM SLEEP QUALITY SCALE (PSQS-14)**

Yang, Chiu-Ling & Yu, Chen-Hsiang & Chen, Chung-Hey. (2013). Development and Validation of the Postpartum Sleep Quality Scale. The journal of nursing research : JNR. 21. 148-54. 10.1097/jnr.0b013e3182921f80.

**COBERTURA DE SALUD**

**134.** ¿Su hijo/a tiene algún tipo de cobertura de cuidado de salud, incluyendo seguro médico, planes prepagados como HMO o planes del gobierno tales como Medicaid? (Medicaid se refiere a un programa de asistencia médica que ofrece cobertura de seguro médico a personas de bajos ingresos y personas con discapacidad. El programa Medicaid es un programa federal-estatal en conjunto que es administrado por los estados. El HMO es una Organización de Mantenimiento de Salud.)

0 ❑ No

1 ❑ Sí

**135.** ¿Su hijo/a está asegurado por Medicaid o el Programa de Seguro Médico Infantil del Estado o S-CHIP?

0 ❑ No

1 ❑ Sí
