# Supplementary material for: Study Design, Protocol and Profile of the Maternal And Developmental Risks from Environmental and Social Stressors (MADRES) Pregnancy Cohort: a Prospective Cohort Study in Predominantly Low-Income Hispanic Women in Urban Los Angeles
Source: BMC Pregnancy Childbirth. 2019 May 30;19:189. doi: 10.1186/s12884-019-2330-7 (PMC6543670; doi:10.1186/s12884-019-2330-7)
Supplement: Supplementary file 25 — Six Month Post Birth Questionnaire. Questionnaire administered 3 months after child participant is born. (DOCX 159 kb) [file 12884_2019_2330_MOESM25_ESM.docx]

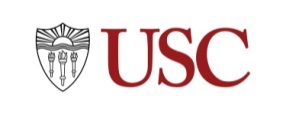
**MADRES Study: Six-Month Questionnaire**

**Today’s Date:** _____________________ **Interviewer Name:** ____________________

**Instructions:** Thank you for participating in the MADRES study. In this interview, I will be asking some questions about you and your baby. Please answer all questions as best as you can, even if you are not completely certain. Be assured that your answers are confidential. Please feel free to interrupt me and ask about anything that is not clear**.**

**CONTACT INFORMATION**

**1. Name:** _________________ _______________ ____________________ ____________________

First Middle Last 1 Last 2

**2. Other names used** (e.g. Maiden name) ­­­­­­­­­­­­­­­­­­­­­­­­­:­___________________________

### **3**. **Your Date of Birth:** **_______/_______/_______**

Month Day Year

**4**. **Baby’s Name:** _________________ ______________ ____________________ _________________

First Middle Last 1 Last 2

### **5**. **Baby’s DOB:** **_______/_______/_______**

Month Day Year

**6**. **Baby’s Gender:** □₁ Female □₂ Male

**7. What is your cell phone number?** ____________________________

□₀ Don’t have a cell phone **(Skip to question #9)**

**8.** **Is this a prepaid cell phone or a permanent phone number?**

□₀ prepaid

□₁ permanent number

**9.** **What is your HOME address (the address at which you spend the most time)?**

Address: ______________________________________________________________________

City: ___________________________State: ________________Zip: _____________________

**9A.** **If moved…When did you move into your new home address?** _______________________

**10. Please tell me the names of other adults living with you:**

Adult#1 First: ______________________Last: ______________________Middle: ______________

Relation to you: ___________________ Cell Phone: ______________________

Adult#2 First: ______________________Last: ______________________Middle: ______________

Relation to you: ___________________ Cell Phone: ______________________

Adult#3 First: ______________________Last: ______________________Middle: ______________

Relation to you: ___________________ Cell Phone: ______________________

**11. What is the phone number for the HOME listed in Question 9?**____________________________

□₀ Don’t have a home phone

**12. Do you live at more than one home?**

□₁ Yes... *Complete questions 13A, 13B and 13C* □₀No… *Go to Question #14*

**13A. What is your second HOME address?**

Address: ______________________________________________________________________

City: ___________________________State: ________________Zip: _____________________

**13A2.** **If moved…When did you move into your new second home address?**  ___________________

**13B. What is the phone number for the HOME listed in Question 13A?** _______________________

□₀ Don’t have a home phone

**13C. How much time do you spend at the address listed in 13A?**

1 ❑ 1%-25% of the time

2 ❑ 26%-50% of the time

**14. A. What is your email address?** _________________________ 0 ❑Don’t have an email address

**B. What is your Facebook username?** ___________________________0 ❑Don’t have Facebook

**C. What is your Twitter handle?** @________________________________0 ❑Don’t have Twitter

**D. What is your Instagram contact name?** _______________________0 ❑Don’t have Instagram

**15.** **A. How do you prefer to be contacted?**

❑ Phone

❑ Email

❑ Text

❑ Other __________

**B. What are the best days to reach you?**

❑ Monday

❑ Tuesday

❑ Wednesday

❑ Thursday

❑ Friday

❑ Saturday

❑ Sunday

**C. What are the best times to reach you (Monday)?**

❑ Mornings (8am-12pm)

❑ Afternoons (12pm-5pm)

❑ Evenings (5pm-8pm)

❑ Other _____________

**D. What are the best times to reach you (Tuesday)?**

❑ Mornings (8am-12pm)

❑ Afternoons (12pm-5pm)

❑ Evenings (5pm-8pm)

❑ Other _____________

**E. What are the best times to reach you (Wednesday)?**

❑ Mornings (8am-12pm)

❑ Afternoons (12pm-5pm)

❑ Evenings (5pm-8pm)

❑ Other _____________

**F. What are the best times to reach you (Thursday)?**

❑ Mornings (8am-12pm)

❑ Afternoons (12pm-5pm)

❑ Evenings (5pm-8pm)

❑ Other _____________

**G. What are the best times to reach you (Friday)?**

❑ Mornings (8am-12pm)

❑ Afternoons (12pm-5pm)

❑ Evenings (5pm-8pm)

❑ Other _____________

**H. What are the best times to reach you (Saturday)?**

❑ Mornings (8am-12pm)

❑ Afternoons (12pm-5pm)

❑ Evenings (5pm-8pm)

❑ Other _____________

**I. What are the best times to reach you (Sunday)?**

❑ Mornings (8am-12pm)

❑ Afternoons (12pm-5pm)

❑ Evenings (5pm-8pm)

❑ Other _____________

**16.** **What is the baby’s father’s name?** □ Don’t know

_________________ _______________ ____________________ ____________________

First Middle Last 1 Last 2

**17A. Do you have a spouse/partner?** 0 ❑ No…*Go to Question 18* 1 ❑ Yes

**17B. What is the name of your spouse/partner**? □ Same as above □ No spouse/partner

_________________ _______________ ____________________ ____________________

First Middle Last 1 Last 2

**18.** **In order to help locate you in case you move and/or change your phone number can you provide us with contact information for your mother and three friends/family members not living with you who would be able to provide us with your new contact information?**

MOTHER’S INFORMATION

First: ______________________Last: ______________________Middle: ______________

Address: ______________________________________________________________________

City: ___________________________State: ________________Zip: _____________________

Cell Phone: ______________________ Home Phone: ______________________

NOK#1

First: ______________________Last: ______________________Middle: ______________

Relation to you: ___________________Email address: ____________________________

Cell Phone: ______________________ Home Phone: ______________________

NOK#2

First: ______________________Last: ______________________Middle: ______________

Relation to you: ___________________Email address: ____________________________

Cell Phone: ______________________ Home Phone: ______________________

NOK#3

First: ______________________Last: ______________________Middle: ______________

Relation to you: ___________________Email address: ____________________________

Cell Phone: ______________________ Home Phone: ______________________

**MAILING ADDRESS**

**19. Do you have a P.O. Box or a mailing address that is different than your home address?**

0 ❑ No

1 ❑ Yes…what is your P.O. Box or mailing address?

Address: _________________________________________________________________

City: ___________________________State: ________________Zip: ________________

**BABY’S HEALTH CARE**

**20A.** Has your baby been to a doctor, or nurse, or other health care worker for a well-baby checkup in the past three months?

0 ❑ No…**SKIP** to Question 21

1 ❑ Yes

**20B.** How many times has your baby been to a doctor, or nurse, or other health care worker for a well-baby checkup in the past three months?

______________TIMES

**21.** Has your baby gone as many times as you wanted for a well-baby checkup?

0 ❑ No

1 ❑ Yes

**22**. Did any of these things keep your baby from having a well-baby checkup? (Select all that apply)

1 ❑ I didn’t have enough money or insurance to pay for it

2 ❑ I had no way to get my baby to the clinic or office

3 ❑ I didn’t have anyone to take care of my other children

4 ❑ I couldn’t get an appointment

5 ❑ My baby was too sick to go for routine care

6 ❑ Other: Please explain: ____________________

7 ❑ NA (Does Not Apply)

**23.** Did your baby have any well-baby shots or vaccinations in the past three months? Do not count shots or vaccinations given in the hospital right after birth.

0 ❑ No

1 ❑ Yes

**24.** Has your child been vaccinated with Tdap (Pertussis or Whooping cough vaccine)?

0 ❑ No

1 ❑ Yes

9 ❑ Don’t know

**25.** Has your child received the flu shot or the nasal flu vaccine?

0 ❑ No

1 ❑ Flu shot

2❑ Nasal FluMist

3 ❑ Both

9 ❑ Don’t know

**26A.** Does your baby currently have a pediatrician or family doctor?

□₀ No… *Go to Question 26B.* □₁ Yes… Please provide doctor’s information.

Physician Name: _________________________________________________________________

Clinic Name: ____________________________________________________________________

Clinic Address: ________________________________________________________________­­___

City: ______________________________State: ____________________Zip: ________________

Phone Number: ________________________________________________________________­­__

Dates: From_____________________*(month/year*) to _________________________*(month/year*)

**26B.** Did your baby have previous pediatricians or family doctors at other clinics?

□₀ No... *Go to Question 27.* □₁ Yes… Please provide doctor’s information.

**Previous Pediatrician #1:**

Physician Name: _________________________________________________________________

Clinic Name: ____________________________________________________________________

Clinic Address: ________________________________________________________________­­___

City: ­­­­­­­­­­­­­­­______________________________State: ____________________Zip: ________________

Phone Number: ________________________________________________________________­­__

Dates: From_____________________*(month/year*) to _________________________*(month/year*)

**Previous Pediatrician #2:**

Physician Name: _________________________________________________________________

Clinic Name: ____________________________________________________________________

Clinic Address: ________________________________________________________________­­___

City: ­­­­­­­­­­­­­­­______________________________State: ____________________Zip: ________________

Phone Number: ________________________________________________________________­­__

Dates: From_____________________*(month/year*) to _________________________*(month/year*)

**STRESS EVALUATION**

**Questions 27-36 Perceived Stress Scale**

Cohen S, Kamarck T, Mermelstein R: **A global measure of perceived stress**. *J Health Soc Behav* 1983, **24**(4):385-396.

**POSTPARTUM DISTRESS MEASURE**

**Questions 37-45 Postpartum Distress Measure**
 Allison KC, Wenzel A, Kleiman K, Sarwer DB: **Development of a brief measure of postpartum distress**. *J Womens Health (Larchmt)* 2011, **20**(4):617-623.

**MOTHER’S WEIGHT**

**46.** Do you know what your current weight is or have a best estimate of your current weight?

9 ❑ Do not know

1 ❑ Yes...

1. What is your current weight or your best estimate of your current weight (in pounds)? _______lbs

**47.** How concerned are you with losing your pregnancy weight?

0 ❑ N/A I have lost all of my pregnancy weight

1 ❑ Not at all concerned

2 ❑ Not very concerned

3 ❑ Somewhat concerned

4 ❑ Very concerned

**OCCUPATIONAL HISTORY**

**48**. What is your current employment status? (**SELECT ALL THAT APPLY**)

1 ❑ Homemaker

2 ❑ Student

3 ❑ Employed

4 ❑ Temporary medical leave

5 ❑ Unemployed

6 ❑ Other: Explain: ____________________

**49.** Have you been working in the past three months?

0 ❑ No (**SKIP TO #54**)

1 ❑ Yes

**50.** How old was the baby when you returned to work?

0 ❑ Less than 1 week old

1 ❑ 1-6 weeks old

2 ❑ 6-12 weeks old

3 ❑ 13-18 weeks old

4 ❑ 19-27 weeks old

**51.** How many hours do you work per week?

1 ❑ Less than 10 hours/week

2 ❑ 10-20 hours/week

3 ❑ 21-30 hours/week

4 ❑ 31-40 hours/week

5 ❑ More than 40 hours/week

**52**. During a regular work week, how many days do you commute to and from your work location?

0 ❑ 0 days

1 ❑ 1 day

2 ❑ 2 days

3 ❑ 3 days

4 ❑ 4 days

5 ❑ 5 days

6 ❑ 6 days

7 ❑ 7 days

**53.** Thinking about your typical commute **TO** your job, which forms of transportation do you use and for how long? (select all that apply.)

|  | **1-10**  **Minutes** | **11-20**  **Minutes** | **21-30**  **Minutes** | **31-59**  **Minutes** | **60-90**  **Minutes** | **91-120**  **Minutes** | **2 Hours or More** | **N/A** |
| --- | --- | --- | --- | --- | --- | --- | --- | --- |
| Car |  |  |  |  |  |  |  |  |
| Bus or  Tram |  |  |  |  |  |  |  |  |
| Train or Metro |  |  |  |  |  |  |  |  |
| By motorcycle |  |  |  |  |  |  |  |  |
| By bike |  |  |  |  |  |  |  |  |
| On foot |  |  |  |  |  |  |  |  |

**SMOKING QUESTIONS**

**54.** Excluding e-cigarettes, have you ever smoked cigarettes, cigars or pipes?

0 ❑ No (**SKIP TO #57**)

1 ❑ Yes

**55.** **In the past three months**, excluding e-cigarettes, have you smoked cigarettes, cigars or pipes?

0 ❑ No (**SKIP TO # 57**)

1 ❑ Yes

**56.** Have you smoked cigarettes, cigars, or pipes in the last 5 days?

0 ❑ No:

**A.** If you are NOT currently smoking, when did you stop smoking? [**SELECT ONE**]

1 ❑ Less than 2 weeks ago

2 ❑ 2 to 4 weeks ago

3 ❑ More than 4 weeks ago

9 ❑ Don't remember

**B.** If you are NOT currently smoking, how many cigarettes did you usually smoke *per day*?

1 ❑ 1- 5

2 ❑ 6-10

3 ❑ 11-20

4 ❑ More than 20

1 ❑ Yes:

**A.** How many cigarettes did you usually smoke *per day*?

1 ❑ 1- 5

2 ❑ 6-10

3 ❑ 11-20

4 ❑ More than 20

**57.** Have you **ever** smoked electronic cigarettes/e-cigarettes or other electronic nicotine device (e-hookah, e-cigars, etc.)?

0 ❑ No (**SKIP TO #60**)

1 ❑ Yes

**58.** **In the past three months,** have you smoked electronic cigarettes/e-cigarettes or other electronic nicotine device (e-hookah, e-cigars, etc.)?

0 ❑ No **(SKIP TO #60)**

1 ❑ Yes

**59.** Have you smoked electronic cigarettes/e-cigarettes or other electronic nicotine device (e-hookah, e-cigars, etc.) in the last 5 days?

0 ❑ No:

**A.** If you are NOT currently smoking, when did you stop smoking? [**SELECT ONE**]

1 ❑ Less than 2 weeks ago 3 ❑ More than 4 weeks ago

2 ❑ 2 to 4 weeks ago 9 ❑ Don't remember

1. If you are NOT currently smoking, how often did you smoke electronic cigarettes/e-cigarettes or other electronic nicotine devices (e-hookah, e-cigars, etc.)?

1❑ Every day 4❑ About once a month

2❑ Every few days 5❑ Every few months

3❑ Once a week

1 ❑ Yes:

**A.** How often do you smoke electronic cigarettes/e-cigarettes or other electronic nicotine devices (e-hookah, e-cigars, etc.)?

1❑ Every day

2❑ Every few days

3❑ Once a week

4❑ About once a month

5❑ Every few months

**60. In the past three months**, excluding e-cigarettes, has anyone living in your home smoked cigarettes, cigars or pipes inside the house?

0 ❑ No (**SKIP TO #63)**

1 ❑ Yes

**61.In the past three months,** who else in your home has smoked cigarettes, cigars or pipes**? (SELECT ALL THAT APPLY)**

1 ❑ Baby's Father

2 ❑ Others

**62.In the past three months,** not including yourself, how many people living in your home smoke cigarettes, cigars or pipes?

1 ❑ 1

2 ❑ 2

3 ❑ 3

4 ❑ 4 or more

**63. In the past three months,** *on average*, how many hours per day have you been *exposed* to cigarette, cigar or pipe smoke because of smoking by others?

1 ❑ 0-1 hour

2 ❑ 1-2 hours

3 ❑ 2-3 hours

4 ❑ 3-4 hours

5 ❑ More than 4 hours

**64. In the past three months,** *on average*, how many hours per day has **your baby** been *exposed* to cigarette, cigar or pipe smoke because of smoking by others?

1 ❑ 0-1 hour

2 ❑ 1-2 hours

3 ❑ 2-3 hours

4 ❑ 3-4 hours

5 ❑ More than 4 hours

**BREASTFEEDING**

**Questions 65-77 Infant Feeding Practices**

Fein SB, Labiner-Wolfe J, Shealy KR, Li R, Chen J, Grummer-Strawn LM: **Infant Feeding Practices Study II: study methods**. *Pediatrics* 2008, **122 Suppl 2**:S28-35.

**CHILD CARE**

**78.**What type of living/custody arrangement do you have with your child?

_1_❑ Your child lives with both parents in the same home.

_2_❑ Your child lives with both parents in separate homes, splitting time about equally.

_3_❑ Your child lives with you most of the time in your home.

_4_❑ Your child lives with the other parent most of the time in a different home from yours.

_5_❑ Your child lives in the same home at all times, but the parents move in and out.

_6_❑ Other (please specify): ____________________________________________________________

**79.In the past three months,** has your baby spent time in a daycare or been taken care of by someone other than yourself**?**

□₀ No… (Skip to #84)

□₁ Yes

**80.** Most of the time, where was the child care provided?

_1_❑ In your own home

_2_❑ In someone else’s home

_3_❑ In a formal daycare program

**81**. During a typical week, how many days per week did these people/programs/centers care for your child? (Including evenings, nights, and weekends)

_1_❑ 5 or more days

_2_❑ 3 – 4 days

_3_❑ 1 – 2 days

_4_❑ Less than 1 day

**82**. On the days when your child was cared for during a typical week, how many hours per day did your child spend with these people/programs/centers?

_1_❑ 5 or more hours

_2_❑ 3 – 4 hours

_3_❑ 1 – 2 hours

_4_❑ Less than 1 hour

**83.** Besides you, what are your current childcare arrangements (Please fill in all that are applicable)?

|  | **Who takes care of your baby?** | **How many days per week?** | **How many hours per day?** | **Where do they take care of your baby? (e.g., grandma’s house, daycare facility)** | **What is the address?** |
| --- | --- | --- | --- | --- | --- |
| **Childcare Arrangement 1** |  |  |  |  |  |
| **Childcare Arrangement 2** |  |  |  |  |  |
| **Childcare Arrangement 3** |  |  |  |  |  |

**BABY’S HEALTH**

**84.** **Has your baby had any of the following conditions over the last three months?**

|  | | **No** | **Yes but did not see doctor** | **Yes and saw a doctor** | **Yes, saw a doctor and received prescription medication** |
| --- | --- | --- | --- | --- | --- |
| a. | Diarrhea | □_0_ | □_1_ | □_2_ | □_3_ |
| b. | Blood in the stool | □_0_ | □_1_ | □_2_ | □_3_ |
| c. | Vomiting | □_0_ | □_1_ | □_2_ | □_3_ |
| d. | Cough | □_0_ | □_1_ | □_2_ | □_3_ |
| e. | High temperature (above 101° F/38° C) | □_0_ | □_1_ | □_2_ | □_3_ |
| f. | Runny or stuffed nose | □_0_ | □_1_ | □_2_ | □_3_ |
| g. | Cold | □_0_ | □_1_ | □_2_ | □_3_ |
| h. | Earache | □_0_ | □_1_ | □_2_ | □_3_ |
| i. | Ear infection or ear discharge (pus not wax) | □_0_ | □_1_ | □_2_ | □_3_ |
| j. | Convulsions/seizures | □_0_ | □_1_ | □_2_ | □_3_ |
| k. | Colic | □_0_ | □_1_ | □_2_ | □_3_ |
| l. | Fussy or irritable | □_0_ | □_1_ | □_2_ | □_3_ |
| m. | Reflux | □_0_ | □_1_ | □_2_ | □_3_ |
| n. | Rash | □_0_ | □_1_ | □_2_ | □_3_ |
| o. | Red itchy or scaly patches on cheeks, scalp, elbows or knees, such as with Eczema | □_0_ | □_1_ | □_2_ | □_3_ |
| p. | A deep croupy or hacking cough, accompanied by wheezing or crackling in the chest, such as with lower respiratory infections like Respiratory Syncytial Virus (RSV), bronchitis or pneumonia | □_0_ | □_1_ | □_2_ | □_3_ |
| q. | An accident/injury. Please describe: | □_0_ | □_1_ | □_2_ | □_3_ |
| r. | Other, please describe: | □_0_ | □_1_ | □_2_ | □_3_ |

**85A.** Has your baby ever had wheezing or whistling in the chest at any time in the past?

□₀ No…*Skip to Question #86* □₁ Yes

**85B**. Tell me the ages when your baby had wheezing or whistling in the chest? (*Select all that apply*)

□₁ Birth to 1 month

□₂ 1 month to 2 months

□₃ 2 months to 3 months

□₄ 3 months to 4 months

□5 4 months to 5 months ❑6 5 months to 6 months

**85C**. How many attacks of wheezing or breathing difficulty has your child had in the past three months?

0 ❑ 1

1 ❑ 2

2 ❑ 3

3 ❑ 4 or more

**86.** Did your baby receive any of the following medicines in the past three months?

|  | **NO_0_** | **YES,**  **Once_1_** | **YES, more than once_2_** |
| --- | --- | --- | --- |
| Antibiotics |  |  |  |
| Other prescription medicines |  |  |  |
| Non-prescription medicines |  |  |  |

**87**. Did [CHILD] receive all prescription medications {he/she} needed?

1 ❑ Yes (**Skip** to Question #88)

2 ❑ No

**[IF NO]** Why did [CHILD] not get medical care or prescription medications {he/she} needed?

1 ❑ Cost Too Much

2 ❑ Health Plan Problem

3 ❑ Not Available in Area or Transport Problem

4 ❑ Not Convenient Time

5 ❑ Doctor Did Not Know How to Treat or Provide Care

6 ❑ Other _____________

**88A.** Has your baby been taken to the doctor/urgent care/emergency room because he/she had any problem you were worried about?

□₀ No… *Go to Question #89.* □₁ Yes… … *Go to question #88B*

**88B.** How many times? _________

**88C.** Please tell me the approximate dates of each doctor/urgent care/emergency room visit and the accompanied reason for the visit.

|  | What were the approximate dates? |  | What was/were the problem(s) you were worried about? |
| --- | --- | --- | --- |
| 1. Month/Day/Year: | __________________________ |  | __________________________________ |
| 2. Month/Day/Year: | __________________________ |  | __________________________________ |
| 3. Month/Day/Year: | __________________________ |  | __________________________________ |
| 4. Month/Day/Year: | __________________________ |  | __________________________________ |

**88D.** Was your baby admitted to the hospital?

□₀No... *Go to Question #89.* □₁ Yes… *Go to question #88E*

**88E.** Please describe each admission**:**

|  | Age of baby (weeks) |  | Reason for admission |  | No. of nights baby stayed overnight |
| --- | --- | --- | --- | --- | --- |
| 1. |  |  |  |  |  |
| 2. |  |  |  |  |  |
| 3. |  |  |  |  |  |
| 4. |  |  |  |  |  |

**89.** Does your baby have any serious, long-term medical problems?

0 ❑ No

1 ❑ Yes

Please Explain Briefly: _______________________

**HOME CHARACTERISTICS**

******For Administrator Only (Do not ask participant):**

Did participant move since last seen on (give date)?

0 ❑ No… Ask 92, 93, 95, 98-104, 106-107

1 ❑ Yes…Ask 90-107

**90**. **Which best describes the home in which you and your baby currently live most of the time**? *Select one.*

1 ❑ A house (not connected to other homes)

2 ❑ A building with 2-4 attached apartments, town houses, condos, a duplex or a triplex

3 ❑ A building with 5-10 attached apartments, town houses, condos, etc.

4 ❑ A building with more than 10 attached apartments, town houses, condos, etc.

5 ❑ Mobile home or trailer

6 ❑ Other, please be specific: ___________________________________________________

**91**. **About when was this structure originally built**? (When it was first constructed, not when it may have been remodeled, added to, or converted.) *Select one.*

1❑ 2000s or later

2❑ 1980s-1990s

3❑ 1960s-1970s

4❑ 1940s-1950s

5❑ Before 1940

**92.** In the past three months, which of the following pets do you keep inside your home? **(SELECT ALL THAT APPLY)**

1 ❑ No Pets

2 ❑ Dog(s)

3 ❑ Cat(s)

4 ❑ Other pets (Explain: ____________)

**93.** **In the past three months**, have you had any of the following pests in your home? **(SELECT ALL THAT APPLY)**

1 ❑ Rats

2 ❑ Mice

3 ❑ Cockroaches

4 ❑ Other pests (Specify: ________________)

5 ❑ Don’t know

6 ❑ No pests

**94**. Is there a cooking stove, range, or oven in your home that uses GAS**?**

0 ❑ No

1 ❑ Yes:

**A.** How often is the gas stove, range or oven used while you are at home? *Select one.*

1 ❑ Never **(SKIP to #95)**

2 ❑ Less than once a week

3 ❑ 1-3 times per week

4 ❑ 4-7 times per week

5 ❑ 8-14 times per week

6 ❑ More than 14 times per week

**B.** About how long is the gas stove, range or oven used on an average day while you are at home?

1 ❑ Less than 15 minutes

2 ❑ 15 minutes to less than 30 minutes

3 ❑ 30 minutes to less than 1 hour

4 ❑ 1 hour or more

**95.** **In the past three months,** on average, how many times a week do you cook (using the stove/range/oven, not microwave)?

_1_ ❑ Never

_2_ ❑ 1 – 3 times a week

_3_ ❑ 4 – 5 times a week

_4_ ❑ Every day of the week

**96.** Does your home have heating?

0 ❑ No **(SKIP to #98)**

1 ❑ Yes:

**A.** What is the main fuel used to heat it? *Select one.*

1 ❑ Gas (you may be able to see a blue flame or pilot light in the unit)

2 ❑ Electricity (you may be able to see a red-hot glowing wire in the unit)

3 ❑ Bottles, tank or L.P./liquefied petroleum gas (a tank outside that a truck may fill with gas)

4 ❑ Firewood

5 ❑ Other, please be specific: ________________________

9 ❑ Don’t know how it is heated

**97.** What is the one main heating system in your home? *Select one.*

1 ❑ Forced air

2 ❑ Built-in electric unit

3 ❑ Wall heater

4 ❑ Floor heater

5 ❑ Portable space heater… **Which type?**

1 ❑ Gas

2 ❑ Electric

3 ❑ Don’t Know

6 ❑ Other, please be specific: ______________________________

9 ❑ Don’t know how it is heated

**98.** Do you use air conditioning in your home?

0 ❑ No **(SKIP to #101)**

1 ❑ Yes:

**A.** What is the main kind of air conditioning that is used? *Select one.*

1 ❑ Wall or window unit (box that sticks out of window or wall)

**a.** How many wall/window units do you have in your home?

_1_ ❑ One

_2_ ❑ Two

_3_ ❑ Three

_4_ ❑ Four or more

_5_ ❑ Don’t know

2 ❑ Central (vents in the room)

3 ❑ Swamp/desert/evaporative cooler

9 ❑ Don’t know what kind it is

**99**. **During the last month,** about how often did you use air conditioning when you were at home?

1 ❑ Never

2 ❑ Less than 5 days

3 ❑ 5-15 days

4 ❑ 16-30 days

9 ❑ Don’t know

**100.** On any given day, how much of the time did you use the air conditioner at home?

_1_ ❑ None of the time

_2_ ❑ A couple of hours a day

_3_ ❑ Half of the time

_4_ ❑ Most of the time

_5_ ❑ All of the time

_9_ ❑ Don’t know

**101.** During the last month, did you use a window fan or other fan that you placed in the window or an attic fan to cool your home?

0 ❑ No

1 ❑ Yes

**102.** Has there been water damage OR flooding in your home **in the past three months?**

0 ❑ No

1 ❑ Yes:

**A.** Did it flood carpeted areas?

0 ❑ No

1 ❑ Yes

9 ❑ Don’t know

**103.** Has there ever been mold or mildew on the walls, ceilings, or floors inside your home **in the past three months?**

0 ❑ No

1 ❑ Yes:

**A.** Which rooms were affected? *Select all that apply.*

1 ❑ The room where you sleep

2 ❑ Bathroom(s)

3 ❑ Basement

4 ❑ Other

9 ❑ Don’t know

**104.** Is a humidifier or vaporizer ever used in your home? (Include humidifier built into heating system.)

0 ❑ No

1 ❑ Yes:

**A.** What type is it? *Select all that apply.*

1 ❑Built into heating system

2 ❑Free standing or portable unit

**B.** Have you used this unit for treating a respiratory illness?

0 ❑No

1 ❑Yes

**C.**  Does the humidifier or vaporizer heat the air?

0 ❑No

1 ❑Yes

9 ❑Don’t know

9 ❑ Don’t know

**105**. Is there carpeting in your home?

0 ❑ No

1 ❑ Yes:

**A.** In what rooms? *Select all that apply.*

1 ❑ Whole house (excluding kitchen and bath)

2 ❑ Room where you sleep

3 ❑ Other bedroom(s)

4 ❑ Other room(s)

**106.** Thinking back to a typical *weekday* in this past **week**, approximately how many hours (out of 24 hours in total) did you spend…

**A.** Outdoors: ________________

**B.** Indoors at home (Include nighttime/sleeping):____________________

**107.** On average, how much of the time were the windows open in your home this past **week**?

_1_ ❑ None of the time

_2_ ❑ A couple of hours a day

_3_ ❑ Half of the time

_4_ ❑ Most of the time

_5_ ❑ All of the time

_9_ ❑ Don’t know

**SLEEP QUESTIONS**

**Next, we are going to ask you about your sleeping patterns and habits during the past month (30 days). Think about over the past month (30 days).**

**108. In the past thirty days**, how many hours of sleep did you usually get on a typical weeknight (Sunday - Thursday)?

_1_ ❑ Less than 4 hours per night _5_ ❑ 8 hours per night

_2_ ❑ 5 hours per night _6_ ❑ 9 hours per night

_3_ ❑ 6 hours per night _7_ ❑ More than 10 hours per night

_4_ ❑ 7 hours per night

**109. In the past thirty days**, how many hours of sleep did you usually get on a typical weekend night (Friday or Saturday)?

_1_ ❑ Less than 4 hours per night _5_ ❑ 8 hours per night

_2_ ❑ 5 hours per night _6_ ❑ 9 hours per night

_3_ ❑ 6 hours per night _7_ ❑ More than 10 hours per night

_4_ ❑ 7 hours per night

**NEIGHBORHOOD COHESION**

**Questions 110-115 Neighborhood Safety**

Sampson RJ, Raudenbush SW, Earls F. Neighborhoods and violent crime: a multilevel study of collective efficacy. Science. 1997 Aug 15;277(5328):918‐24.

**FINANCIAL STRESS**

**Questions 116-122 Economic Hardship**

Feather NT. Reported changes in behaviour after job loss in a sample of older unemployed men. Australian Journal of Psychology. 1989;41(2):175–185

**FOOD INSECURITY**

**Questions 123-124**

Food Security in the U.S. Measurement USDA <https://www.ers.usda.gov/topics/food-nutrition-assistance/food-security-in-the-us/measurement.aspx#survey>

**AGES AND STAGES**

**Questions 125-160 Ages and Stages 6 months**

J. Squires, D. Bricker **Ages & Stages Questionnaires®, Third Edition (ASQ- 3™). A parent-completed child-monitoring system** Paul H. Brookes Publishing Co., Baltimore(2009)
