# Supplementary material for: Study Design, Protocol and Profile of the Maternal And Developmental Risks from Environmental and Social Stressors (MADRES) Pregnancy Cohort: a Prospective Cohort Study in Predominantly Low-Income Hispanic Women in Urban Los Angeles
Source: BMC Pregnancy Childbirth. 2019 May 30;19:189. doi: 10.1186/s12884-019-2330-7 (PMC6543670; doi:10.1186/s12884-019-2330-7)
Supplement: Supplementary file 26 — Six Month Post Birth Questionnaire_Spanish. Spanish questionnaire administered 6 months after child participant is born. (DOCX 163 kb) [file 12884_2019_2330_MOESM26_ESM.docx]

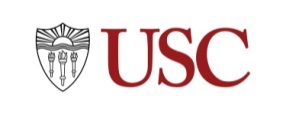
**MADRES Study: Six Month Questionnaire**

**Today’s Date:** _____________________ **Interviewer Name:** ____________________

**Instrucciones:** Gracias por aceptar participar en el estudio MADRES. Durante esta entrevista le hare preguntas sobre usted y su bebe. Por favor responda a todas las preguntas de este cuestionario, aún si no está completamente segura de la respuesta. Le aseguramos que sus respuestas serán confidenciales**.** Por favor, con toda confianza, siéntase libre de interrumpirme y hacerme cualquier pregunta que tenga.

**INFORMACION DE CONTACTO**

**1. Nombre:** ________________ __________________ __________________ ____________________

Nombre 2do Nombre Apellido 1 Apellido 2

**2. Otros nombres que haya usado** (ej. nombre de soltera) ­­­­­­­­­­­­­­­­­­­­­­­­­:­___________________________

**3**. **Fecha de nacimiento:** _**______/_______/_______**

Mes Día Año

**4**. **Nombre del Bebé:** __________________ ________________ __________________ _________________

Nombre 2do Nombre Apellido 1 Apellido 2

**5. Fecha de nacimiento del Bebé:** **_______/_______/_______**

Mes Día Año

**6**. **Género del Bebé:** □₁ Femenino □₂ Masculino

**7. ¿Cuál es su número de celular?** ____________________________

□₀ No tengo teléfono celular **(Skip to question #9)**

**8.** **¿Es un celular pre pagado o es un número fijo de celular?**

□₀ Pre pagado

□₁ Número fijo

**9.** **¿Cuál es su dirección? (la dirección donde usted pasa la mayoría del tiempo):**

Dirección: ________________________________________________________________________

Ciudad: _____________________Estado: ________________Código Postal: ___________________

**9A.** **If moved…¿Cuándo se mudó a su nueva dirección?** _______________________

**10. Por favor dígame los nombres de otros adultos que viven con usted:**

Adult#1 Nombre: ______________________Apellido: ______________________2do Nombre: ______________

Relación: ___________________ Número de Celular: ______________________

Adult#2 Nombre: ______________________Apellido: ______________________2do Nombre: ______________

Relación: ___________________ Número de Celular: ______________________

Adult#3 Nombre: ______________________Apellido: ______________________2do Nombre: ______________

Relación: ___________________ Número de Celular: ______________________

**11. ¿Cuál es el número de teléfono para el domicilio dado en la Pregunta 9?**______________________

□₀ No tengo teléfono de casa

**12. ¿Vive en más de una casa?**

□₁ Sí... *Complete questions 13A, 13B and 13C* □₀No…  *Go to question #14*

**13A. ¿Cuál es la dirección de su segundo domicilio?**

Dirección: _________________________________________________________________________

Ciudad: ______________________Estado: ________________ Código Postal: __________________

**13A2.** **If moved…¿Cuándo se mudó a su nuevo segundo domicilio?** _______________________

**13B. ¿Cuál es el número de teléfono para el domicilio dado en la Pregunta 13A?** _____________________

□₀ No tengo teléfono de casa

**13C. ¿Cuánto tiempo pasa usted en la dirección dada en la pregunta 13A?**

 1%-25% del tiempo

 26%-50% del tiempo

**14. A. ¿Cuál es su correo electrónico?** _________________________ 0 ❑No tengo correo electrónico

**B. ¿Cuál es su nombre de usuario en Facebook?** ___________________________0 ❑No tengo Facebook

**C. ¿Cuál es su nombre de usuario en Twitter?** @___________________________0 ❑No tengo Twitter

**D. ¿Cuál es su nombre de contacto para Instagram?** ____________________0 ❑No tengo Instagram

**15. A. ¿Cómo prefiere ser contactada?**

 Teléfono

 Correo electrónico

 Texto

 Otro: ________________

**B. ¿Cuáles son los mejores días para contactarla?**

 lunes

 martes

 miércoles

 jueves

 viernes

 sábado

 domingo

**C. ¿Cuáles son las mejores horas para contactarla (lunes)?**

 En la mañana (8am-12pm)

 En la tarde (12pm-5pm)

 En la noche (5pm-8pm)

 Otro: ______________

**D. ¿Cuáles son las mejores horas para contactarla (martes)?**

 Mañana (8am-12pm)

 Tarde (12pm-5pm)

 Noche (5pm-8pm)

 Otro: ______________

**E. ¿Cuáles son las mejores horas para contactarla (miércoles)?**

 Mañana (8am-12pm)

 Tarde (12pm-5pm)

 Noche (5pm-8pm)

 Otro: ______________

**F. ¿Cuáles son las mejores horas para contactarla (jueves)?**

 Mañana (8am-12pm)

 Tarde (12pm-5pm)

 Noche (5pm-8pm)

 Otro: ______________

**G. ¿Cuáles son las mejores horas para contactarla (viernes)?**

 Mañana (8am-12pm)

 Tarde (12pm-5pm)

 Noche (5pm-8pm)

 Otro: ______________

**H. ¿Cuáles son las mejores horas para contactarla (sábado)?**

 Mañana (8am-12pm)

 Tarde (12pm-5pm)

 Noche (5pm-8pm)

 Otro: ______________

**I . ¿Cuáles son las mejores horas para contactarla (domingo)?**

 Mañana (8am-12pm)

 Tarde (12pm-5pm)

 Noche (5pm-8pm)

 Otro: ______________

**16.** **¿Cómo se llama el papa del bebé?** □ No sé

_________________ _______________ ____________________ ____________________

Nombre 2do Nombre Apellido 1 Apellido 2

**17A.** **¿Tiene usted esposo o pareja?** 0 ❑ No…*Go to Question* 18 1 ❑ Sí

**17B. ¿Cómo se llama su esposo/pareja?**

_________________ _______________ ____________________ ____________________

Nombre 2do Nombre Apellido 1 Apellido 2

**18.** **Para poder localizarla en caso de que se mude o cambie su número de teléfono, ¿nos puede dar la información de su madre y tres amigos o familiares que no vivan con usted que nos podrían dar su información nueva?**

INFORMACION DE SU MADRE

Nombre: ______________________Apellido: ______________________2do Nombre: ______________

Dirección: _________________________________________________________________________

Ciudad: ______________________Estado: ________________ Código Postal: __________________

Número de Celular: ______________________ Número de teléfono de Casa: ______________________

NOK#1

Nombre: ______________________Apellido: ______________________2do Nombre: ______________

Relación: ___________________Correo Electrónico: ____________________________

Número de Celular: ______________________Número de teléfono de Casa: ______________________

NOK#2

Nombre: ______________________Apellido: ______________________2do Nombre: ______________

Relación: ___________________Correo Electrónico: ____________________________

Número de Celular: ______________________Número de teléfono de Casa: ______________________

NOK#3

Nombre: ______________________Apellido: ______________________2do Nombre: ______________

Relación: ___________________Correo Electrónico: ____________________________

Número de Celular: ______________________Número de teléfono de Casa: ______________________

**DIRECCIÓN DE ENVIO**

**19. ¿Tiene una dirección postal o postal diferente a la dirección de su domicilio?**

0 ❑ No

1 ❑ Sí… ¿cual es su dirección postal?

Dirección: _________________________________________________________________________

Ciudad: ______________________Estado: ________________ Código Postal: __________________

**CUIDADO DE SALUD DEL BEBÉ**

**20A.** ¿Su bebé ha sido visto por un médico, una enfermera u otro profesional de la salud para un chequeo de rutina en los últimos tres meses?

0 ❑ No…**SKIP** to Question 21

1 ❑ Sí

**20B.** ¿Cuántas veces ha sido visto su bebé por un médico, una enfermera u otro profesional de la salud para un chequeo de rutina en los últimos tres meses?

______________VECES

**21.** ¿Su bebé ha ido tantas veces como usted ha querido a los chequeos de rutina?

0 ❑ No

1 ❑ Sí

**22.** ¿Alguna de estas cosas evito que su bebé tuviera un chequeo de salud de rutina? **Select all that apply**

1 ❑ No tuve suficiente dinero ni seguro médico para pagar por ello

2 ❑ No tenía manera para llevar a mi hijo/a a la clínica u oficina

3 ❑ No tenía alguien para cuidar de mis otros hijos

4 ❑ No pude conseguir una cita

5 ❑ Mi bebé estaba muy enfermo para ir al cuidado de rutina

6 ❑ Otros: Por favor, explique:____________________

7 ❑ NA (No se aplica)

**23.** ¿Ha tenido su bebe alguna vacuna de niño sano u otras vacunas en los últimos tres meses? No cuente inyecciones o vacunas administradas en el hospital inmediatamente después del nacimiento.

0 ❑ No

1 ❑ Sí

**24.** ¿Su hijo/a ha sido vacunado con la vacuna Tdap (tos ferina o la vacuna contra la tos ferina)?

0 ❑ No

1 ❑ Sí

9 ❑ No sé

**25.** ¿Su hijo/a ha recibido la vacuna contra la gripe o la vacuna nasal contra la gripe?

0 ❑ No

1 ❑ Vacuna contra la gripe

2❑ FluMist nasal

3 ❑ Ambas

9 ❑ No sé

**26A.** ¿Tiene su bebé un pediatra o médico de cabecera?

□₀ No… *Continúe a la Pregunta 26B.* □₁ Sí…Por favor provea la información del pediatra.

Nombre del doctor: _________________________________________________________________

Nombre de la clínica: ____________________________________________________________________

Dirección de la clínica: ________________________________________________________________­­___

Ciudad: _____________________________Estado: ___________________Código postal:_____________

Número de teléfono: ________________________________________________________________­­__

Fechas: De____________________*(mes/año*) a _________________________*(mes/año*)

**26B**. **¿**Ha tenido su bebé previamente otros pediatras o médicos de cabecera?

□₀ No… *Continúe a la Pregunta 27* □₁ Sí…Por favor provea la información del pediatra.

**Pediatra Previo #1:**

Nombre del doctor: _________________________________________________________________

Nombre de la clínica: ____________________________________________________________________

Dirección de la clínica: ________________________________________________________________­­___

Ciudad: _____________________________Estado: ___________________Código postal:_____________

Número de teléfono: ________________________________________________________________­­__

Fechas: De_____________________*(mes/año*) a _________________________*(mes/año*)

**Pediatra Previo #2:**

Nombre del doctor: _________________________________________________________________

Nombre de la clínica: ____________________________________________________________________

Dirección de la clínica: ________________________________________________________________­­___

Ciudad: _____________________________Estado: ___________________Código postal:_____________

Número de teléfono: ________________________________________________________________­­__

Fechas: De_____________________*(mes/año*) a _________________________*(mes/año*)

**EVALUACION DEL ESTRES**

**Questions 27-36 Perceived Stress Scale**

Cohen S, Kamarck T, Mermelstein R: **A global measure of perceived stress**. *J Health Soc Behav* 1983, **24**(4):385-396.

**POSTPARTUM DISTRESS MEASURE**

**Questions 37-45 Postpartum Distress Measure**
 Allison KC, Wenzel A, Kleiman K, Sarwer DB: **Development of a brief measure of postpartum distress**. *J Womens Health (Larchmt)* 2011, **20**(4):617-623.

**PESO DE LA MADRE**

**46.** ¿Sabe usted su peso actual o la mejor estimación de su peso actual?

9 ❑ No sé

1 ❑ Sí…

**A.** ¿Cuál es su peso actual o su mejor estimación de su peso actual (en libras)?

_______ lbs

**47.** ¿Cuánto le preocupa perder el peso del embarazo?

0 ❑ N/A He perdido todo el peso del embarazo

1 ❑ No está preocupada en absoluto

2 ❑ No está muy preocupada

3 ❑ Está un poco preocupada

4 ❑ Está muy preocupada

**HISTORIA OCUPACIONAL**

**48**. ¿Cuál es su actual estado de empleo? [SELECT ALL THAT APPLY]

1 ❑ Ama de casa 4 ❑ En permiso médico tempora

2 ❑ Estudiante 5 ❑ Desempleada

3 ❑ Empleada 6 ❑ Otro: (Explique)

**49.** ¿Ha estado trabajando en los últimos tres meses?

0 ❑ No (**SKIP TO #54**)

1 ❑ Sí

**50.** ¿Qué edad tenía su bebé cuando regresó al trabajo?

0 ❑ Menos de 1 semana de edad

1 ❑ 1-6 semanas de edad

2 ❑ 6-12 semanas de edad

3 ❑ 13-18 semanas de edad

4 ❑ 19-27 semanas de edad

**51.** ¿Cuántas horas a la semana trabaja?

1 ❑ Menos de 10 horas/semana

2 ❑ 10-20 horas/semana

3 ❑ 21-30 horas/semana

4 ❑ 31-40 horas/semana

5 ❑ Más de 40 horas/semana

**52**. Durante una semana de trabajo regular, ¿cuantos días a la semana le toca viajar de ida y de regreso del trabajo?

0 ❑ 0 día

1 ❑ 1 día

2 ❑ 2 días

3 ❑ 3 días

4 ❑ 4 días

5 ❑ 5 días

6 ❑ 6 días

7 ❑ 7 días

**53.** Piensa acerca de tu viaje típico de ida al trabajo, ¿qué formas de transporte utilizas y por cuánto tiempo? (**Select all that apply.)**

|  | **1-10**  **Minutos** | **11-20**  **Minutos** | **21-30**  **Minutos** | **31-59**  **Minutos** | **60-90**  **Minutos** | **91-120**  **Minutos** | **2 Horas o mas** | **N/A** |
| --- | --- | --- | --- | --- | --- | --- | --- | --- |
| Coche/carro |  |  |  |  |  |  |  |  |
| Bus o  Tranvía |  |  |  |  |  |  |  |  |
| Tren o Metro |  |  |  |  |  |  |  |  |
| Motocicleta |  |  |  |  |  |  |  |  |
| Bicicleta |  |  |  |  |  |  |  |  |
| A pie |  |  |  |  |  |  |  |  |

**PREGUNTAS SOBRE EL USO DEL CIGARRILLO**

**54.** Sin incluir cigarrillos eléctricos, ¿Ha fumado usted cigarrillos, cigarros o pipas alguna vez en su vida?

0 ❑ No (**SKIP TO #57**)

1 ❑ Sí

**55.** **En los últimos tres meses,** sin incluir cigarrillos eléctricos, ¿ha fumado usted cigarrillos, cigarros o pipas?

0 ❑ No (**SKIP TO # 57**)

1 ❑ Sí

**56.** ¿Ha fumado usted cigarrillos, cigarros o pipas en los últimos 5 días?

0 ❑ No:

**A.** Si NO fuma actualmente**,** ¿cuándo dejo de fumar? [**SELECT ONE**]

1 ❑ Hace menos de 2 semanas

2 ❑ 2 a 4 semanas atrás

3 ❑ Más de 4 semanas atrás

4 ❑ No recuerdo

**B.** Si NO fuma actualmente**,** ¿Cuántos cigarrillos fumaba al día?

1 ❑ 1- 5 3 ❑ 11-20

2 ❑ 6-10 4 ❑ Más de 20

1 ❑ Sí:

**A.** ¿Cuántos cigarrillos fuma al día?

1 ❑ 1- 5 3 ❑ 11-20

2 ❑ 6-10 4 ❑ Más de 20

**57.** ¿Ha fumado usted cigarrillos electrónicos o algún otro sistema electrónico de administración de nicotina (e-hookah, e-cigars, etc.)?

0 ❑ No (**SKIP TO #60**)

1 ❑ Sí

**58.** **En los últimos tres meses**, ¿Ha fumado usted cigarrillos electrónicos o algún otro sistema electrónico de administración de nicotina (e-hookah, e-cigars, etc.)?

0 ❑ No **(SKIP TO #60)**

1 ❑ Sí

**59.** ¿Ha fumado usted cigarrillos electrónicos o algún otro sistema electrónico de administración de nicotina (e-hookah, e-cigars, etc.) en los últimos 5 días?

0 ❑ No:

**A.** Si NO fuma actualmente**,** ¿cuándo dejo de fumar? [**SELECT ONE**]

1 ❑ Hace menos de 2 semanas

2 ❑ 2 a 4 semanas atrás

3 ❑ Más de 4 semanas atrás

4 ❑ No recuerdo

**B.** Si NO fuma actualmente**,** ¿Qué tan seguido fumaba cigarrillos electrónicos o algún otro sistema electrónico de administración de nicotina (e-hookah, e-cigars, etc.)?

1❑ Todos los días

2❑ Cada dos o tres días

3❑ Una vez a la semana

4❑ Aproximadamente una vez al mes

5❑ Cada cuantos meses

1 ❑ Sí:

**A.** ¿Qué tan seguido fuma cigarrillos electrónicos o algún otro sistema electrónico de administración de nicotina (e-hookah, e-cigars, etc.)?

1❑ Todos los días

2❑ Cada dos o tres días

3❑ Una vez a la semana

4❑ Aproximadamente una vez al mes

5❑ Cada cuantos meses

**60. En los últimos tres meses,** sin incluir cigarrillos electrónicos, ¿Alguna otra persona que vive en su casa ha fumado cigarrillos, cigarros o pipas dentro de la casa?

0 ❑ No (**SKIP TO #63)**

1 ❑ Sí

**61.** **En los últimos tres meses,** ¿Quién más en su casa ha fumado cigarrillos, cigarros o pipas?  **(SELECT ALL THAT APPLY)**

1 ❑ Padre del bebe

2 ❑ Otras personas

**62. En los últimos tres meses,** sin incluirse a usted misma ¿Cuántas personas viviendo en su casa fuman cigarrillos, cigarros o pipas?

1 ❑ 1

2 ❑ 2

3 ❑ 3

4 ❑ 4 o más

**63.** **En los últimos tres meses,** *en un promedio*, ¿Cuántas horas al día está expuesta usted al humo de cigarrillos, cigarros o pipas fumados por otras personas?

1 ❑ 0-1 hora

2 ❑ 1-2 horas

4 ❑ 3-4 horas

5 ❑ Más de 4 horas

3 ❑ 2-3 horas

**64.** **En los últimos tres meses,** *en un promedio*, ¿Cuántas horas al día está expuesto/a **su** **bebé** al humo de cigarrillos, cigarros o pipas fumados por otras personas?

1 ❑ 0-1 hora

2 ❑ 1-2 horas

4 ❑ 3-4 horas

5 ❑ Más de 4 horas

3 ❑ 2-3 horas

**AMAMANTAMIENTO**

**Questions 65-77 Infant Feeding Practices**

Fein SB, Labiner-Wolfe J, Shealy KR, Li R, Chen J, Grummer-Strawn LM: **Infant Feeding Practices Study II: study methods**. *Pediatrics* 2008, **122 Suppl 2**:S28-35.

**CUIDADO DEL BEBÉ**

**78.**¿Qué tipo de arreglo de alojamiento / custodia tiene usted con su hijo/a?

_1_❑ Su hijo/a vive con ambos padres en la misma casa.

_2_❑ Su hijo/a vive con ambos padres en casas separadas, dividiendo el tiempo casi por igual.

_3_❑ Su hijo/a vive con usted la mayor parte del tiempo en su casa.

_4_❑ Su hijo/a vive con el otro padre la mayor parte del tiempo en una casa diferente a la suya.

_5_❑ Su hijo/a vive en la misma casa todo momento, pero los padres entran y salen.

_6_❑ Otro (Por favor especifique): _________________________________________________________

**79.** **Durante los últimos tres meses,** ¿su bebé ha pasado tiempo en una guardería o sido cuidado por alguien que no sea usted?

□₀ No… (**Skip to #84**)

□₁ Sí

**80.** La mayor parte del tiempo, ¿en qué lugar fue cuidado su bebé?

_1_❑ En su propia casa

_2_❑ En la casa de otra persona

_3_❑ En un programa formal de guardería

**81**. Durante una semana típica, ¿cuántos días por semana cuidan estas personas / programas / centros a su bebé? (Incluyendo tardes, noches, y fines de semana)

_1_❑ 5 o más días

_2_❑ 3 - 4 días

_3_❑ 1- 2 días

_4_❑ Menos de 1 día

**82**. En los días en que su bebé fue cuidado por alguien más en una semana típica, ¿cuántas horas por día pasa su bebé con estas personas / programas / centros?

_1_❑ 5 o más horas

_2_❑ 3 - 4 horas

_3_❑ 1- 2 horas

_4_❑ Menos de 1 hora

**83.** Además de usted, ¿cuáles son sus actuales servicios de guardería (Por favor, díganos todos los que son aplicables)?

|  | **¿Quién cuida de su bebé?** | **¿Cuántos días a la semana?** | **¿Cuántas horas al día?** | **¿Dónde cuidan de su bebé? (e.g., casa de la abuela, servicio de guardería)** | **¿Cuál es la dirección?** |
| --- | --- | --- | --- | --- | --- |
| **Arreglo de cuidado de niños 1** |  |  |  |  |  |
| **Arreglo de cuidado de niños 2** |  |  |  |  |  |
| **Arreglo de cuidado de niños 3** |  |  |  |  |  |

**SALUD DEL BEBÉ**

**84.** **¿Ha tenido su bebé alguna de las siguientes condiciones en los últimos tres meses?**

|  | | **No** | **Sí, pero no vio médico** | **Sí y vio a un médico** | **Sí, vio un médico y recibió medicina prescrita** |
| --- | --- | --- | --- | --- | --- |
| a. | Diarrea | □_0_ | □_1_ | □_2_ | □_3_ |
| b. | Sangre en el excremento | □_0_ | □_1_ | □_2_ | □_3_ |
| c. | Vomito | □_0_ | □_1_ | □_2_ | □_3_ |
| d. | Toz | □_0_ | □_1_ | □_2_ | □_3_ |
| e. | Alta temperatura (más de 101° F/38° C) | □_0_ | □_1_ | □_2_ | □_3_ |
| f. | Nariz que moquea | □_0_ | □_1_ | □_2_ | □_3_ |
| g. | Resfriado | □_0_ | □_1_ | □_2_ | □_3_ |
| h. | Dolor de oído | □_0_ | □_1_ | □_2_ | □_3_ |
| i. | Descarga del oído (pus no cera) | □_0_ | □_1_ | □_2_ | □_3_ |
| j. | Convulsiones | □_0_ | □_1_ | □_2_ | □_3_ |
| k. | Cólico | □_0_ | □_1_ | □_2_ | □_3_ |
| l. | Inquietud o irritable | □_0_ | □_1_ | □_2_ | □_3_ |
| m. | Reflujo | □_0_ | □_1_ | □_2_ | □_3_ |
| n. | Salpullido | □_0_ | □_1_ | □_2_ | □_3_ |
| o. | Manchas rojas que pican o escamosos en las mejillas, el cuero cabelludo, los codos o las rodillas, como con eczema | □_0_ | □_1_ | □_2_ | □_3_ |
| p. | Una profunda tos seca, acompañada de silbidos o chisporroteo en el pecho, como con infecciones respiratorias como el virus sincitial respiratorio (VSR), bronquitis o neumonía | □_0_ | □_1_ | □_2_ | □_3_ |
| q. | Un accidente / lesión. Por favor describa: | □_0_ | □_1_ | □_2_ | □_3_ |
| r. | Otro. Por favor describa: | □_0_ | □_1_ | □_2_ | □_3_ |

**85A.** ¿Su bebé ha tenido silbidos en el pecho alguna vez en el pasado?

□₀ No…*Skip to Question #86* □₁ Sí

**85B**. ¿ Cuáles son las edades en las que su bebé tuvo silbidos en el pecho? (*Marque todo lo que corresponda*)

□₁ Nacimiento a un mes □_5_ 4 meses a 5 meses

□₂ 1 mes a 2 meses □_6_ 5 meses a 6 meses

□₃ 2 meses a 3 meses

□₄ 3 meses a 4 meses

**85C**. ¿Cuántos ataques de sibilancias o dificultad para respirar ha tenido su bebé en los últimos tres meses?

0 ❑ 1

1 ❑ 2

2 ❑ 3

3 ❑ 4 o más

**86.** ¿Ha recibido su bebé alguno de los siguientes medicamentos en los últimos tres meses?

|  | **NO_0_** | **Sí,**  **una vez_1_** | **Sí, más de una vez_2_** |
| --- | --- | --- | --- |
| Antibióticos |  |  |  |
| Otros medicamentos recetados |  |  |  |
| Medicamentos sin receta |  |  |  |

**87.** ¿Recibió su hijo/a todos los medicamentos recetados que él/ella necesitaba?

□_1_ Sí (**Skip** to Question #88)

□_2_ No

**[IF NO]** ¿Por qué su hijo/a no recibió la atención médica o medicamentos recetados que él/ella necesitaba?

□₁ Cuesta demasiado

□_2_ Problemas con el plan de salud

□_3_ No disponible en la zona o problemas de transporte

□_4_ Tiempos no convenientes

□_5_ El médico no sabía cómo tratar o proporcionar atención

□_6_ Otra razón ______________________

**88A.** ¿Ha llevado a su hijo/a al doctor/cuidado urgente/sala de emergencia por que él/ella tenía un problema que a usted le preocupaba?

□₀ No… *Go to Question #89.* □₁ Sí … *Go to question #88B*

**88B.** ¿Cuántas veces? _________

**88C. Por favor dígame las fechas aproximadas de cada visita al médico / atención de urgencia / a la sala de emergencia y la razón acompañada por la visita.**

|  | ¿Cuáles fueron las fechas aproximadas? |  | ¿Cuál era / eran el problema (s) por las cuales estabas preocupada? |
| --- | --- | --- | --- |
| 1.Mes/Día/Año: | __________________________ |  | __________________________________ |
| 2. Mes/Día/Año: | __________________________ |  | __________________________________ |
| 3. Mes/Día/Año: | __________________________ |  | __________________________________ |
| 4. Mes/Día/Año: | __________________________ |  | __________________________________ |

**88D.** ¿Fue su hijo/a admitido/a al hospital?

□₀No... *Go to Question 91.* □₁ Sí … *Go to question 90d*

**88E.** Por favor describa cada admisión:

|  | Edad del niño/a (semanas) |  | Motivo de admisión |  | Número de noches que el/la niño/a permaneció hospitalizado/a |
| --- | --- | --- | --- | --- | --- |
| 1. |  |  |  |  |  |
| 2. |  |  |  |  |  |
| 3. |  |  |  |  |  |
| 4. |  |  |  |  |  |

**89.** ¿Su bebé tiene algún problema médico grave de largo plazo?

0 ❑ No

1 ❑ Sí

Por favor, explique brevemente: __________________________________________________

**CARACTERISTICAS DEL HOGAR**

******For Administrator Only (Do not ask participant):**

Did participant move since the baby was born (give date)?

0 ❑ No… Ask 92, 93, 95, 98-104, 106-107

1 ❑ Yes… Ask 90-107

**90**. **¿Cuál opción describe mejor la casa en la cual reside actualmente la mayor parte del tiempo?** *Seleccione una sola respuesta.*

1 ❑ Una casa (que no está unida a otras casas)

2 ❑ Un edificio de 2-4 departamentos unidos, townhome, condominio, dúplex o triplex

3 ❑ Un edificio de 5-10 departamentos unidos, townhome, condominio, etc.

4 ❑ Un edificio de más de 10 departamentos unidos, townhome, condominio, etc.

5 ❑ Una casa móvil (“mobile home”) o en un tráiler

6 ❑ Otro, por favor explique: ___________________________________________________

**91**. **Aproximadamente, ¿cuándo fue esta vivienda originalmente construida?** (cuando se construyó por primera vez, no cuando pudo haber sido remodelada o modificada). *[Select one]*

1❑ 2000s o más reciente

2❑ 1980s-1990s

3❑ 1960s-1970s

4❑ 1940s-1950s

5❑ Antes de 1940

**92.** **En los últimos tres meses,** ¿Cuáles de las siguientes mascotas ha tenido/tiene dentro de su casa? **(SELECT ALL THAT APPLY)**

1 ❑ No tengo mascotas

2 ❑ Perro(s)

3 ❑ Gato(s)

4 ❑ Otras mascotas (Explique: ____________)

**93.** **En los últimos tres meses,** ¿Ha tenido Usted alguna de las siguientes infestaciones en su casa? **(SELECT ALL THAT APPLY)**

1 ❑ Ratas

2 ❑ Ratones

3 ❑ Cucarachas

4 ❑ Otras infestaciones (Explique:____________)

5 ❑ No sé

6 ❑ Ningún problema con infestaciones

**94**. ¿Tiene en su casa una estufa u horno de GAS?

0 ❑ No

1 ❑ Sí:

**A.** ¿Con que frecuencia usa la estufa u horno mientras que usted está en casa? *Select one.*

1 ❑ Nunca **(SKIP to #95)**

2 ❑ Menos de una vez por semana

3 ❑ 1-3 veces por semana

4 ❑ 4-7 veces por semana

5 ❑ 8-14 veces por semana

6 ❑ Más de 14 veces por semana

**B.** En promedio, ¿Por cuánto tiempo se usa la estufa u horno durante el día mientras que usted está en casa?

1 ❑ Menos de 15 minutos

2 ❑ 15 minutos a menos de 30 minutos

3 ❑ 30 minutos a menos de 1 hora

4 ❑ 1 hora o más

**95.** **En los últimos tres meses,** en promedio, ¿cuántas veces a la semana cocina usted (usando la estufa /horno, no incluyendo el uso del microondas)?

_1_❑ Nunca _3_❑ 4 – 5 veces por semana

_2_❑ 1 – 3 veces por semana _4_❑ Todos los días

**96.** ¿Su casa tiene sistema de calefacción o calentón?

0 ❑ No **(SKIP to #98)**

1 ❑ Sí:

**A.** ¿Cuál es el principal combustible utilizado para calentar la casa? *Select one.*

1 ❑ Gas (podrá ver una llama azul o el piloto encendido dentro de la unidad)

2 ❑ Eléctrico (podrá ver un alambre o metal ardiente dentro de la unidad)

3 ❑ Un tanque de gas (un tanque o cilindro fuera de la casa que se puede llenar de gas)

4 ❑ Leña

5 ❑ Otro, por favor explique: ________________________

9 ❑ No sé cómo se calienta

**97.** ¿Cuál es el principal sistema de calefacción en su casa? *Select one.*

1 ❑ Aire forzado

2 ❑ Unidad eléctrica pegada a la pared

3 ❑ Calentón ubicado en la pared

4 ❑ Calentón ubicado en el piso

5 ❑ Calentador portátil…**¿Qué tipo?**

1 ❑ Gas

2 ❑ Eléctrico

3 ❑ No sé

6 ❑ Otro, por favor explique: ______________________________

9 ❑ No sé cómo se calienta

**98.** ¿Usa usted aire acondicionado en su casa?

0 ❑ No **(SKIP to #101)**

1 ❑ Sí:

**A.** ¿Cuál es el tipo principal de aire acondicionado que se utiliza? *Select one.*

1 ❑ Aire acondicionado de ventana o pared (caja que sale de la ventana o pared)

**a.** ¿Cuántas unidades de ventana/pared tiene usted en su casa?

_1_ ❑ Uno

_2_ ❑ Dos

_3_ ❑ Tres

_4_ ❑ Cuatro o más

_5_ ❑ No sé

2 ❑ Central (escape de aire en las recamaras)

3 ❑ Enfriador de vapor (“swamp cooler”)

9 ❑ No sé qué tipo sea

**99**. **Durante el último mes,** ¿con que frecuencia uso el aire acondicionado estando en casa?

1 ❑ Nunca

2 ❑ Menos de 5 días

3 ❑ 5-15 días

4 ❑ 16-30 días

9 ❑ No sé

**100.** En un día cualquiera, ¿cuánto tiempo uso usted el aire acondicionado en su casa?

_1_ ❑ Nunca

_2_ ❑ Un par de horas al día

_3_ ❑ La mitad del tiempo

_4_ ❑ La mayor parte del tiempo

_5_ ❑ Todo el tiempo

_9_ ❑ No sé

**101.** **Durante el último mes,** ¿usó usted un ventilador de ventana o algún otro ventilador que puso en la ventana o en el ático para enfriar su casa?

0 ❑ No

1 ❑ Sí

**102.** ¿Ha habido daños por agua o inundaciones en su casa en **los últimos tres meses**?

0 ❑ No

1 ❑ Sí:

**A.** ¿Inundó áreas alfombradas?

0 ❑ No

1 ❑ Sí

9 ❑ No sé

**103.** ¿Ha habido alguna vez moho en las paredes, techos, o pisos dentro de su casa en **los últimos tres meses**?

0 ❑ No

1 ❑ Sí:

**A.** ¿Qué cuartos quedaron afectados? *Select all that apply.*

1 ❑ El cuarto donde duerme

2 ❑ Baño (s)

3 ❑ Sótano

4 ❑ Otro

9 ❑ No sé

**104.** ¿Se ha utilizado un humificador o vaporizador en su casa? (Incluyendo el humificador que puede tener dentro el sistema de calefacción.)

0 ❑ No

1 ❑ Sí:

**A.** ¿Qué tipo es? *Select all that apply.*

1 ❑ Viene dentro el sistema de calefacción

2 ❑ Una unidad portátil

**B.** ¿Ha utilizado este aparato para tratar alguna enfermedad respiratoria?

0 ❑No

1 ❑Sí

**C.**  ¿Calienta el aire el humificador o vaporizador?

0 ❑No

1 ❑ Sí

9 ❑ No sé

**105**. ¿Hay alfombra en su casa?

0 ❑ No

1 ❑ Sí:

**A.** ¿En qué cuartos? *Select all that apply.*

1 ❑ Toda la casa (excluyendo la cocina y baño)

2 ❑ Recamara donde duerme

3 ❑ Otras recamara(s)

4 ❑ Otros cuarto(s)

**106.** Recordando un día típico entresemana de la **semana pasada**, aproximadamente cuantas horas (de 24 horas en total) estuvo…

**A.** Afuera: ________________

**B.** Adentro de la casa (Incluyendo la noche/durmiendo):____________________

**107.** En promedio, ¿cuánto tiempo mantuvo las ventanas abiertas durante esta última **semana**?

_1_ ❑ Nunca

_2_ ❑ Un par de horas al día

_3_ ❑ La mitad del tiempo

_4_ ❑ La mayor parte del tiempo

_5_ ❑ Todo el tiempo

_9_ ❑ No sé

**PREGUNTAS SOBRE COMO DUERME**

**A continuación, vamos a preguntarle acerca de sus patrones y hábitos de dormir durante el último mes (30 días). Piense en el último mes (30 días).**

**108. En el último mes,** ¿cuántas horas de sueño consiguió normalmente en una semana típica (el domingo - jueves)?

_1_ ❑ Menos de 4 horas por noche _5_ ❑ 8 horas por noche

_2_ ❑ 5 horas por noche _6_ ❑ 9 horas por noche

_3_ ❑ 6 horas por noche _7_ ❑ Más de 10 horas por noche

_4_ ❑ 7 horas por noche

**109.** **En el último mes,** ¿cuántas horas de sueño consiguió normalmente en una típica noche de fin de semana (viernes a sábado)?

_1_ ❑ Menos de 4 horas por noche _5_ ❑ 8 horas por noche

_2_ ❑ 5 horas por noche _6_ ❑ 9 horas por noche

_3_ ❑ 6 horas por noche _7_ ❑ Más de 10 horas por noche

_4_ ❑ 7 horas por noche

**COHESION DEL VECINDARIO**

**Questions 110-115 Neighborhood Safety**

Sampson RJ, Raudenbush SW, Earls F. Neighborhoods and violent crime: a multilevel study of collective efficacy. Science. 1997 Aug 15;277(5328):918‐24.

**ESTRÉS FINANCIERO**

**Questions 116-122 Economic Hardship**

Feather NT. Reported changes in behaviour after job loss in a sample of older unemployed men. Australian Journal of Psychology. 1989;41(2):175–185

**INSEGURIDAD ALIMENTARIA**

**Questions 123-124**

Food Security in the U.S. Measurement USDA [https://www.ers.usda.gov/topics/food-nutrition-assistance/food-security-in-the-us/measurement.aspx#survey](https://www.ers.usda.gov/topics/food-nutrition-assistance/food-security-in-the-us/measurement.aspx#survey)

**EDADES Y ETAPAS**

**Questions 125-160 Ages and Stages 6 months**

J. Squires, D. Bricker **Ages & Stages Questionnaires®, Third Edition (ASQ- 3™). A parent-completed child-monitoring system** Paul H. Brookes Publishing Co., Baltimore(2009)
